# Correction: Structural characterization of NrnC identifies unifying features of dinucleases

**DOI:** 10.7554/eLife.77092

**Published:** 2022-01-28

**Authors:** Justin D Lormand, Soo-Kyoung Kim, George A Walters-Marrah, Bryce A Brownfield, J Christopher Fromme, Wade C Winkler, Jonathan R Goodson, Vincent T Lee, Holger Sondermann

**Keywords:** Other

Lormand JD, Kim SK, Walters-Marrah GA, Brownfield BA Fromme JC, Winkler WC, Lee VT, Sondermann H. 2021. Structural characterization of NrnC identifies unifying features of dinucleases. *eLife* **10**:e70146. doi: 10.7554/eLife.70146

Published 17, September 2021

After publication, we realized that the designations ‘nucleotidase’ (used as ‘diribonucleotidase’ or ‘dinucleotidase’ in the manuscript) have been reserved for a different enzymatic reaction than the one described in the manuscript. The term ‘nucleotidase’ usually refers to enzymes that hydrolyze nucleotides into nucleosides and phosphate. However, the enzymes under investigation in this study cleave a phosphodiester bond, converting dinucleotides into mononucleotides, which would be appropriately described as ‘nuclease’ activity, or more precisely, ‘diribonuclease’ or ‘dinuclease’ activity. This change in terminology has no impact on any of the results, their interpretation, or discussions reported in the paper; on the contrary, the correction will prevent any potential confusion regarding the chemical reaction we report on.

We have corrected the manuscript to refer to the appropriate enzyme classification by replacing all instances of ‘diribonucleotidase’ and ‘dinucleotidase’ with ‘diribonuclease’ and ‘dinuclease’, respectively.

