# Supplementary figures and images for: Structural characterization of NrnC identifies unifying features of dinucleases
Source: eLife. 2021 Sep 17;10:e70146. doi: 10.7554/eLife.70146 (PMC8492067; doi:10.7554/eLife.70146)

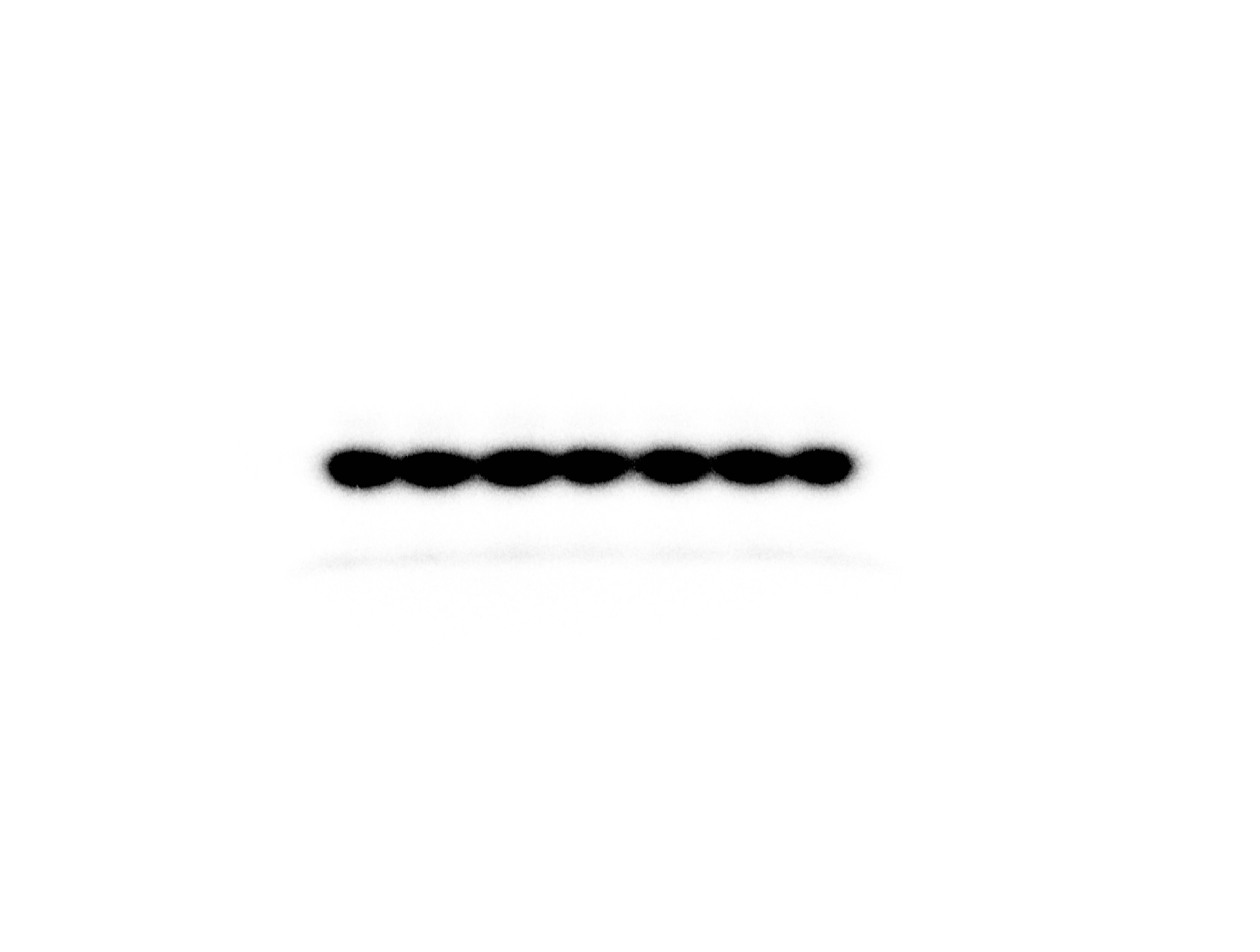

Supplement: Figure 2—source data 1. — Original, unedited images, labeled overview, and quantification of wild-type and mutant NrnC activity using pGG as the substrate from three replicates. [file elife-70146-fig2-data1.zip › Figure2_source_data_1/Figure 2A-source data 3 (D25A).tif]

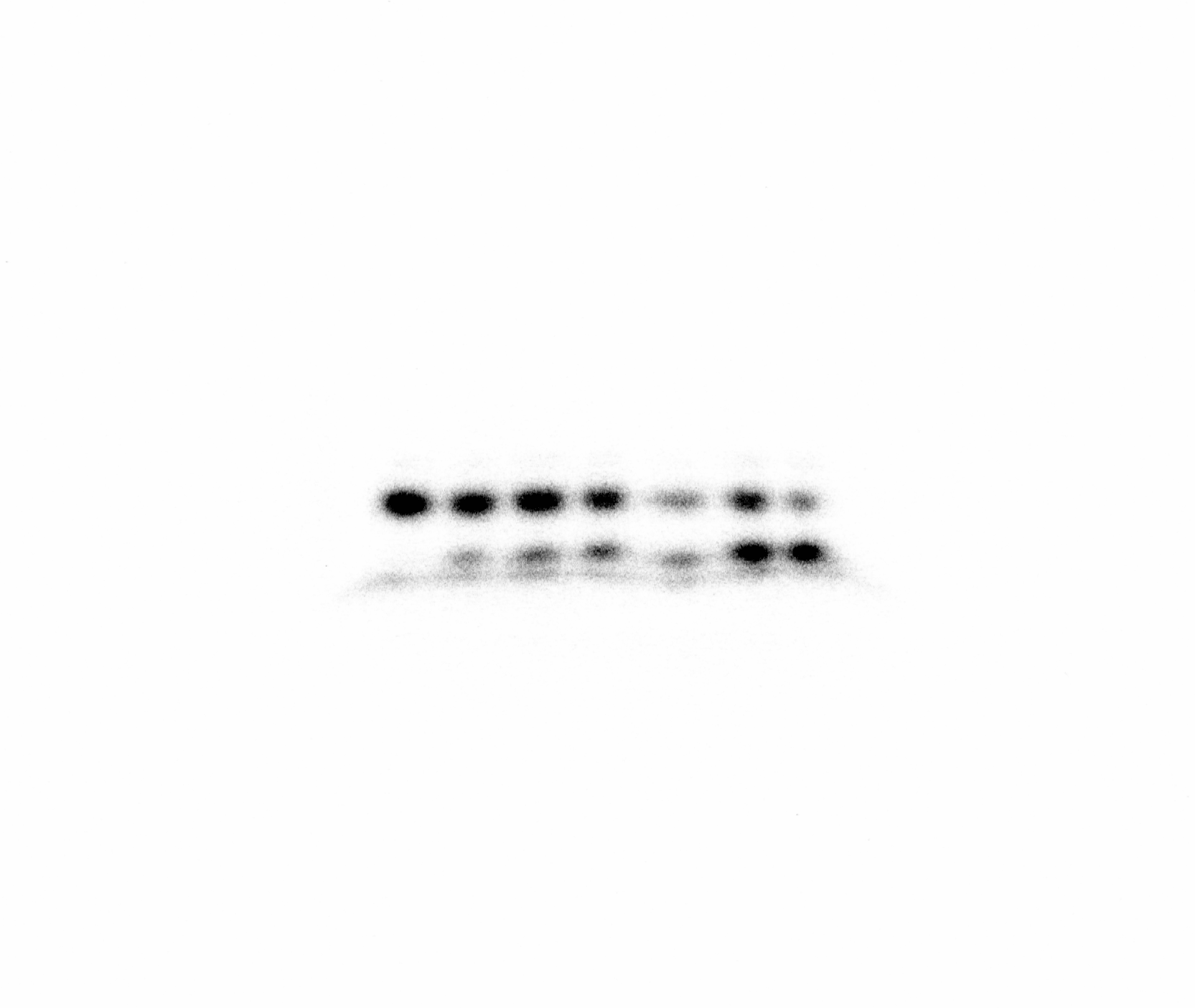

Supplement: Figure 2—source data 1. — Original, unedited images, labeled overview, and quantification of wild-type and mutant NrnC activity using pGG as the substrate from three replicates. [file elife-70146-fig2-data1.zip › Figure2_source_data_1/Figure 2A-source data 2 (H205A).tif]

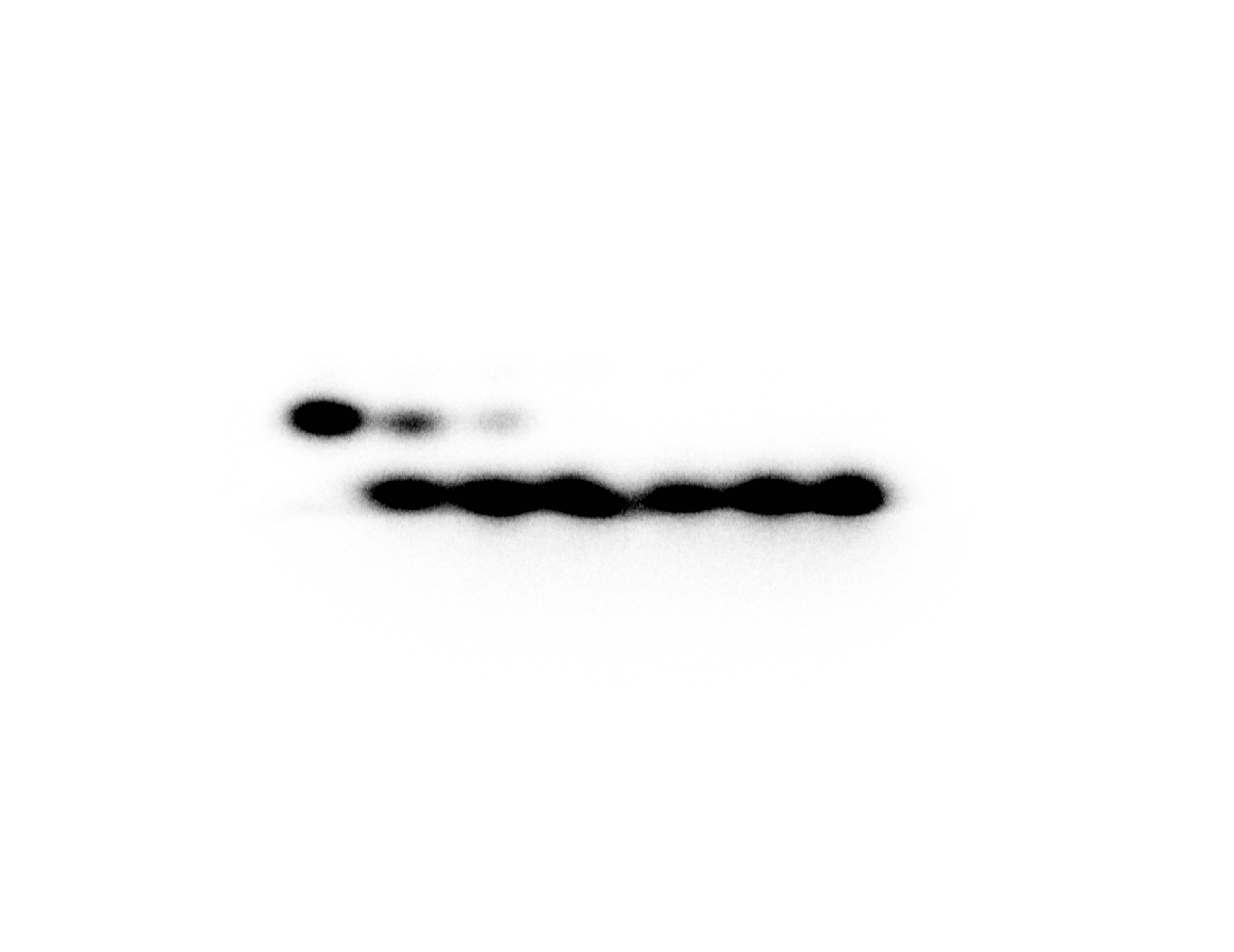

Supplement: Figure 2—source data 1. — Original, unedited images, labeled overview, and quantification of wild-type and mutant NrnC activity using pGG as the substrate from three replicates. [file elife-70146-fig2-data1.zip › Figure2_source_data_1/Figure 2A-source data 3 (WT).tif]

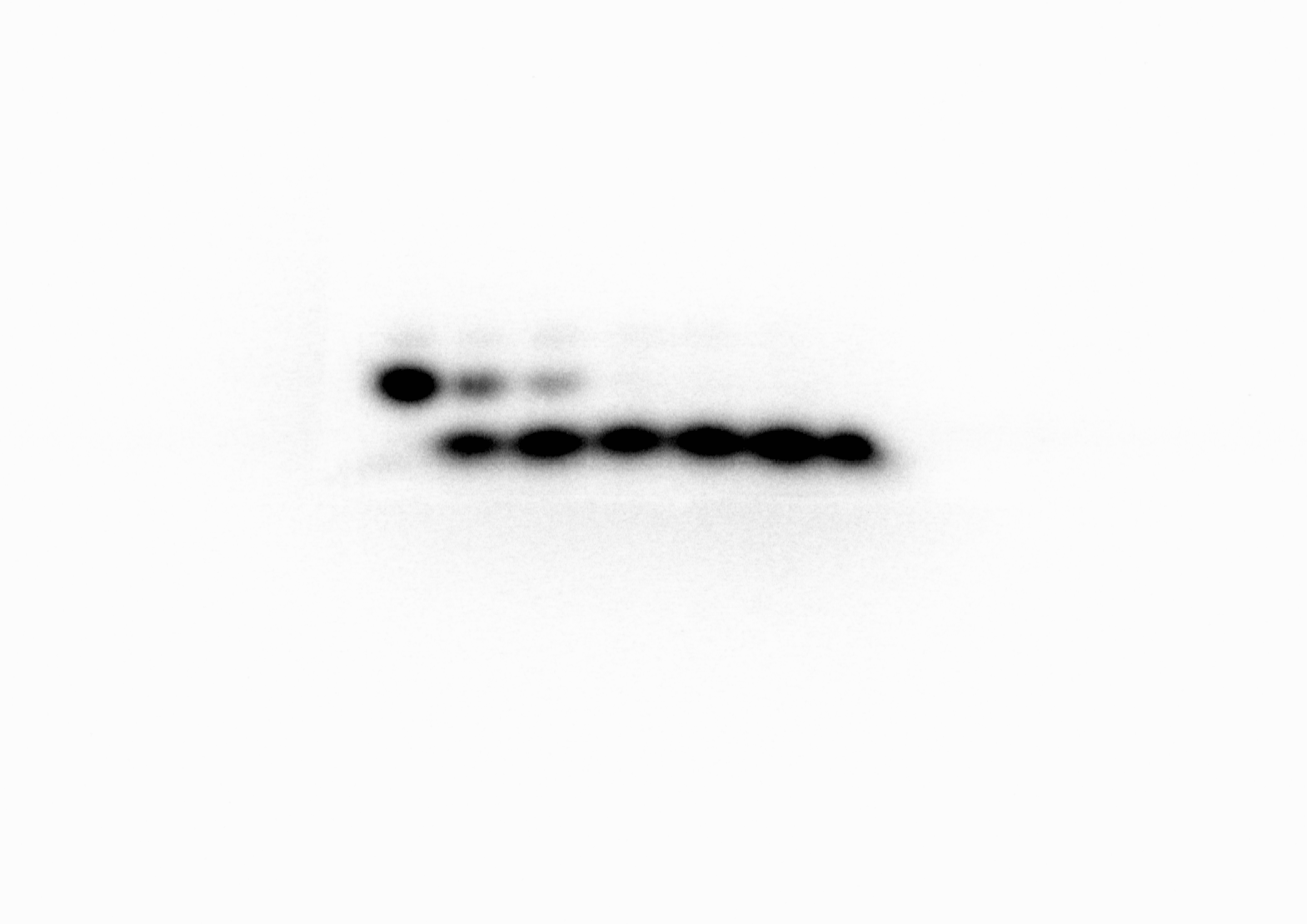

Supplement: Figure 2—source data 1. — Original, unedited images, labeled overview, and quantification of wild-type and mutant NrnC activity using pGG as the substrate from three replicates. [file elife-70146-fig2-data1.zip › Figure2_source_data_1/Figure 2A-source data 2 (WT).tif]

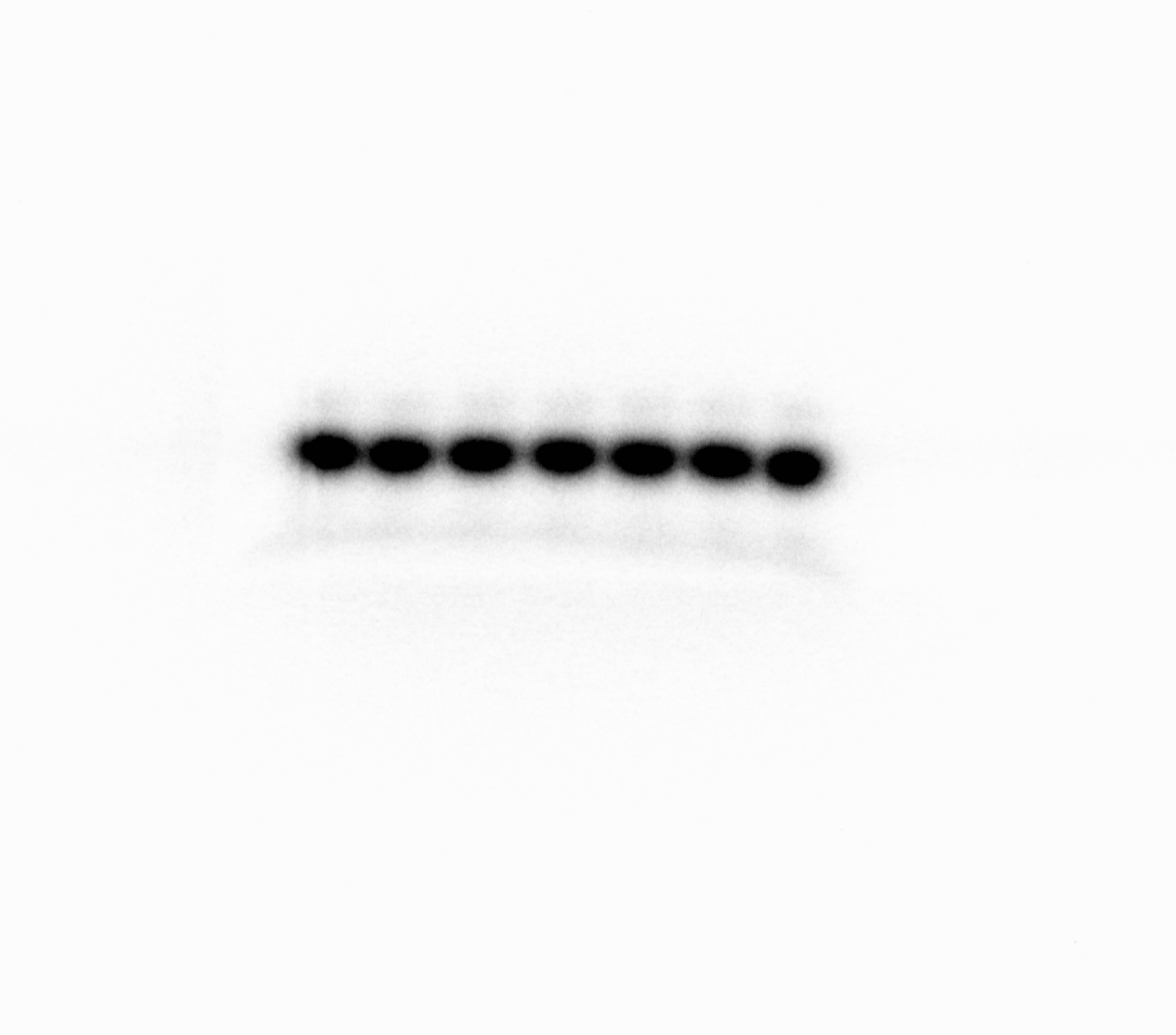

Supplement: Figure 2—source data 1. — Original, unedited images, labeled overview, and quantification of wild-type and mutant NrnC activity using pGG as the substrate from three replicates. [file elife-70146-fig2-data1.zip › Figure2_source_data_1/Figure 2A-source data 1 (Y151A).tif]

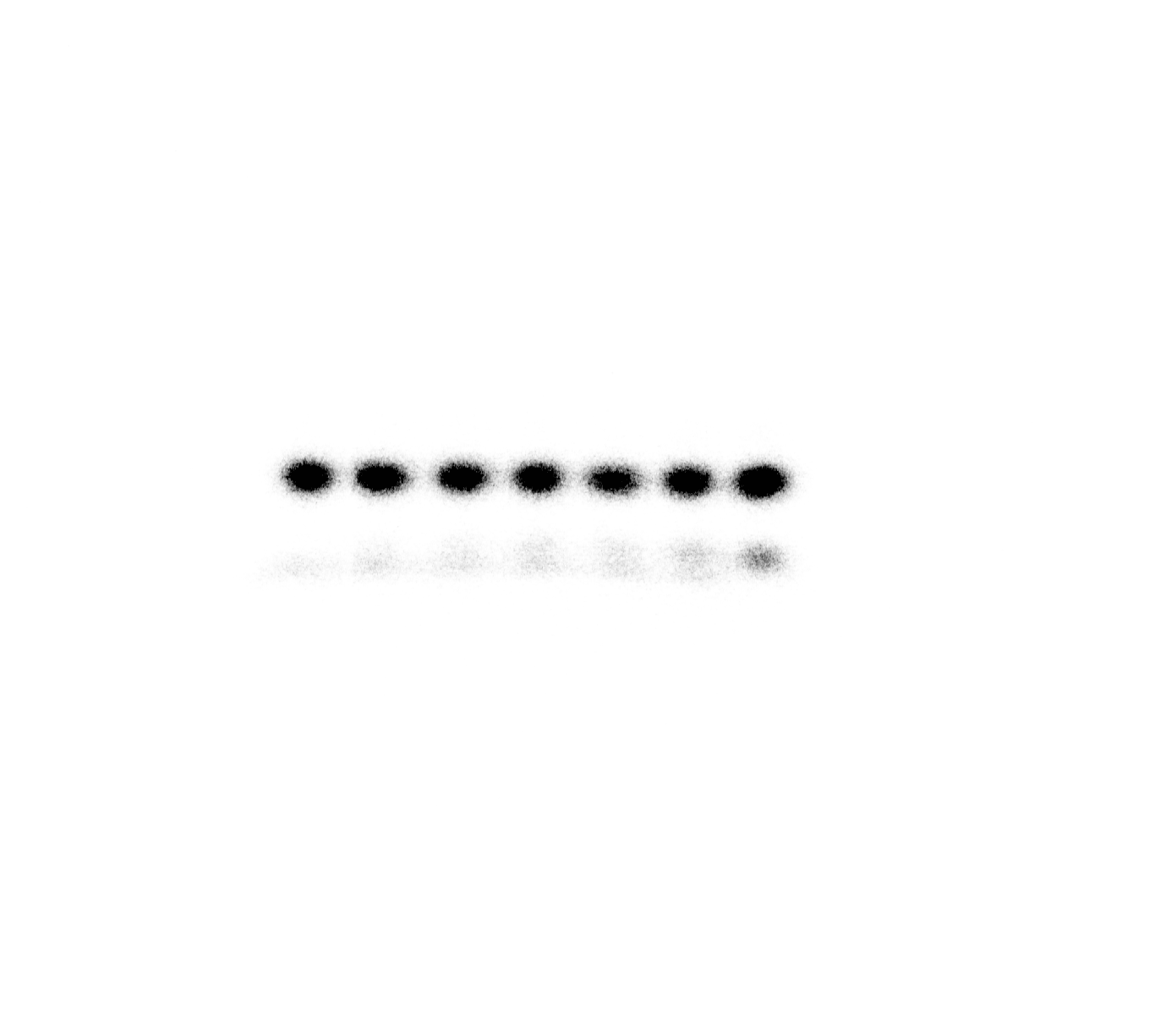

Supplement: Figure 2—source data 1. — Original, unedited images, labeled overview, and quantification of wild-type and mutant NrnC activity using pGG as the substrate from three replicates. [file elife-70146-fig2-data1.zip › Figure2_source_data_1/Figure 2A-source data 2 (L31A).tif]

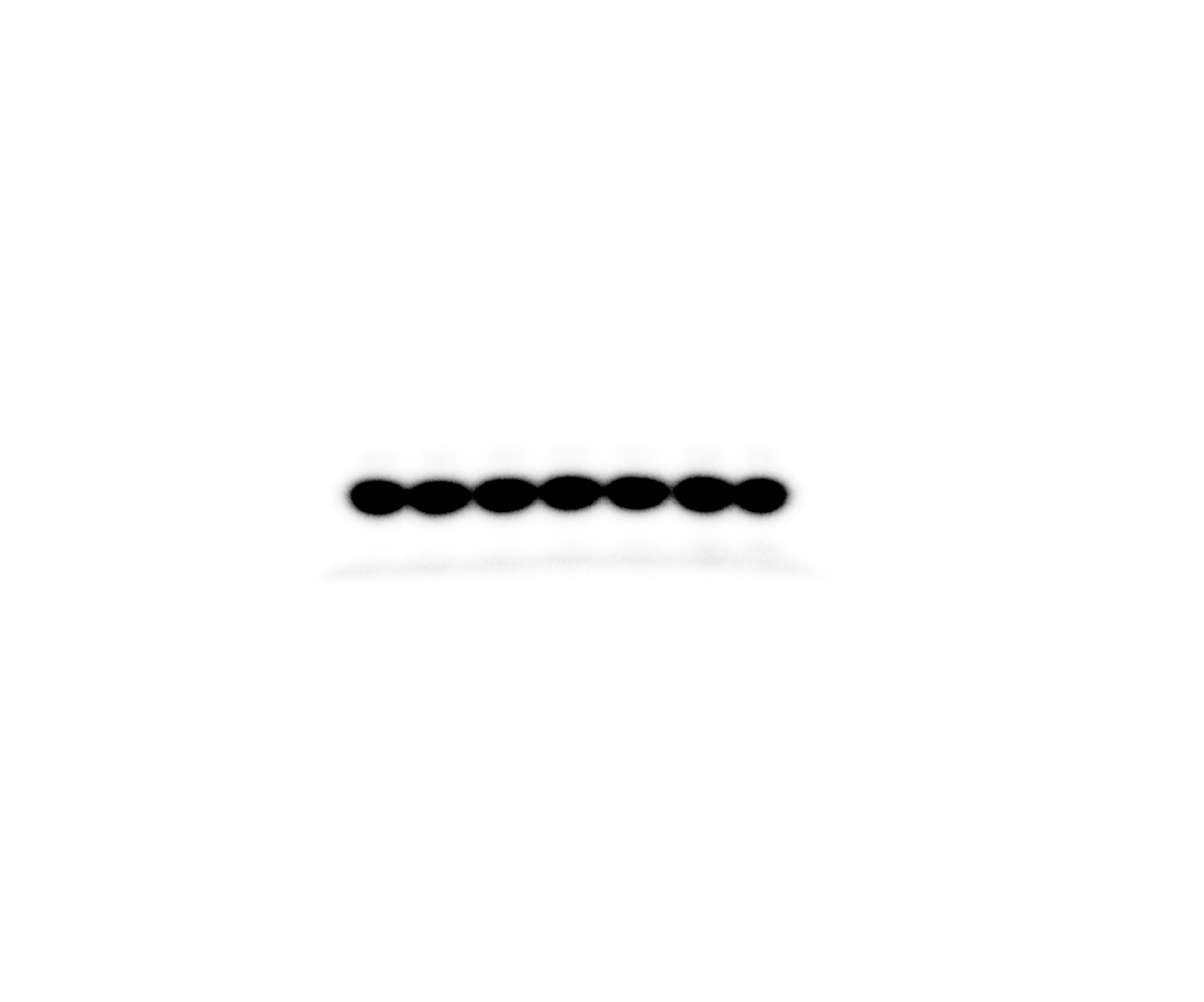

Supplement: Figure 2—source data 1. — Original, unedited images, labeled overview, and quantification of wild-type and mutant NrnC activity using pGG as the substrate from three replicates. [file elife-70146-fig2-data1.zip › Figure2_source_data_1/Figure 2A-source data 2 (Y151A).tif]

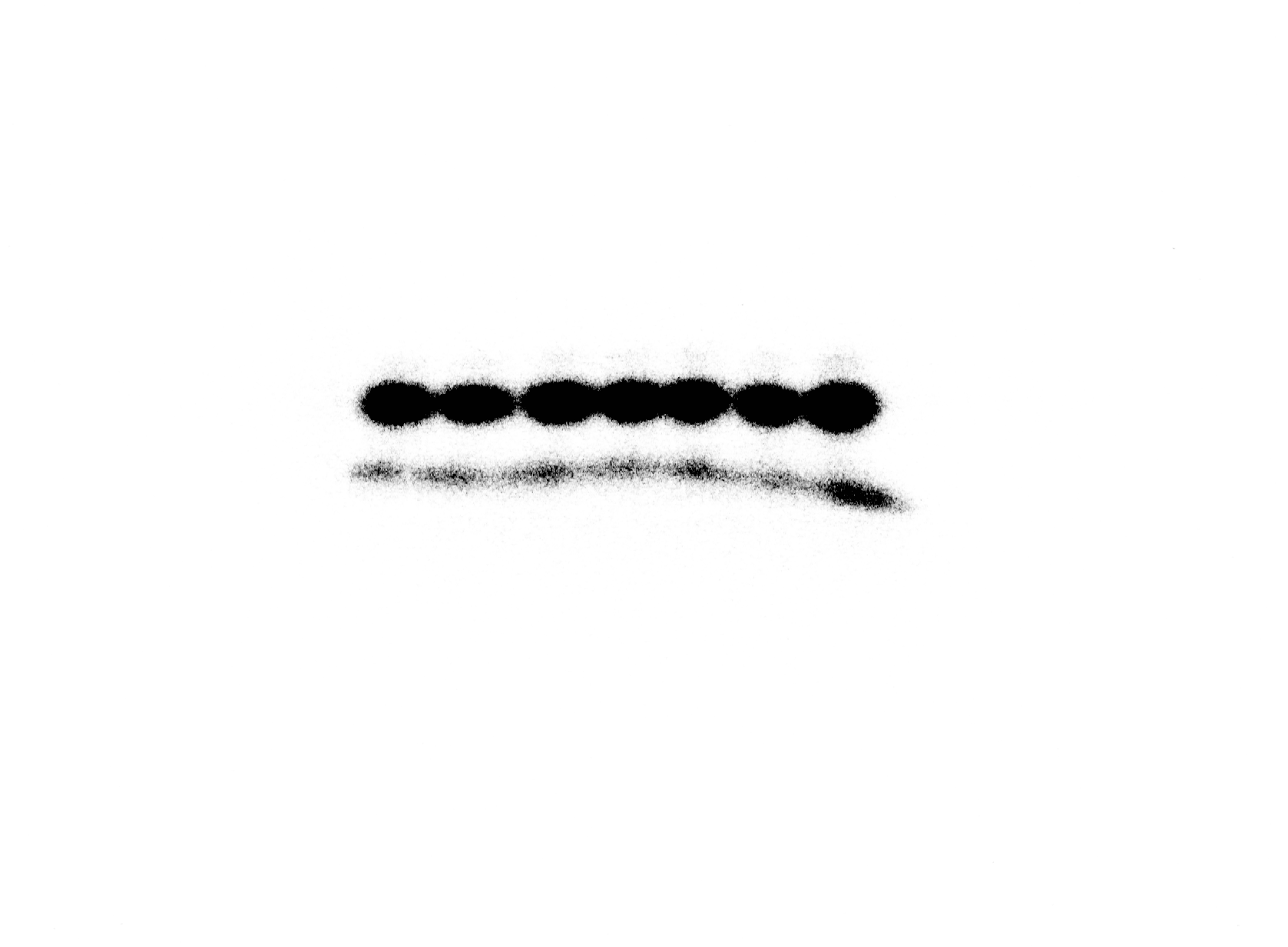

Supplement: Figure 2—source data 1. — Original, unedited images, labeled overview, and quantification of wild-type and mutant NrnC activity using pGG as the substrate from three replicates. [file elife-70146-fig2-data1.zip › Figure2_source_data_1/Figure 2A-source data 1 (D25A).tif]

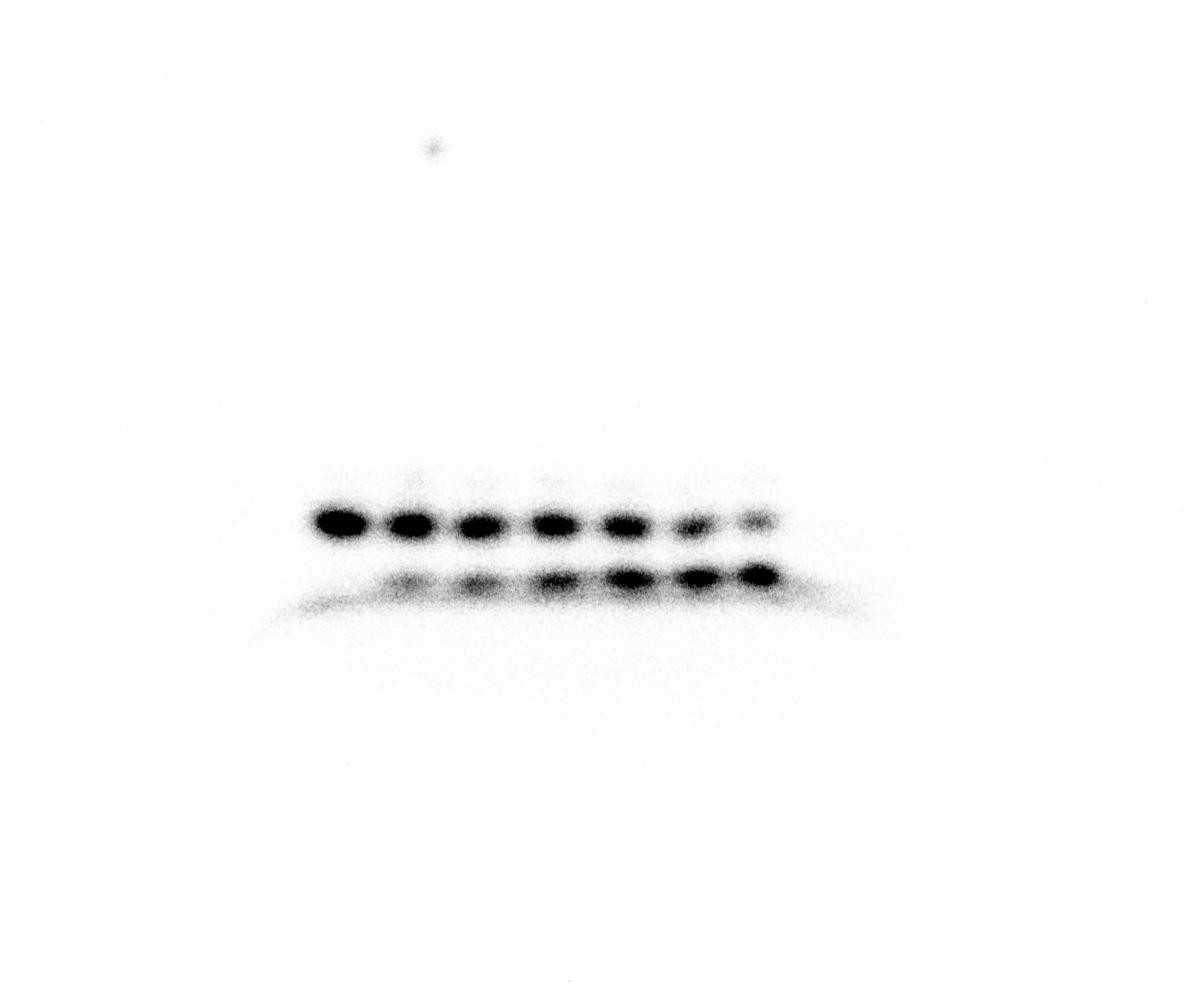

Supplement: Figure 2—source data 1. — Original, unedited images, labeled overview, and quantification of wild-type and mutant NrnC activity using pGG as the substrate from three replicates. [file elife-70146-fig2-data1.zip › Figure2_source_data_1/Figure 2A-source data 1 (H205A).tif]

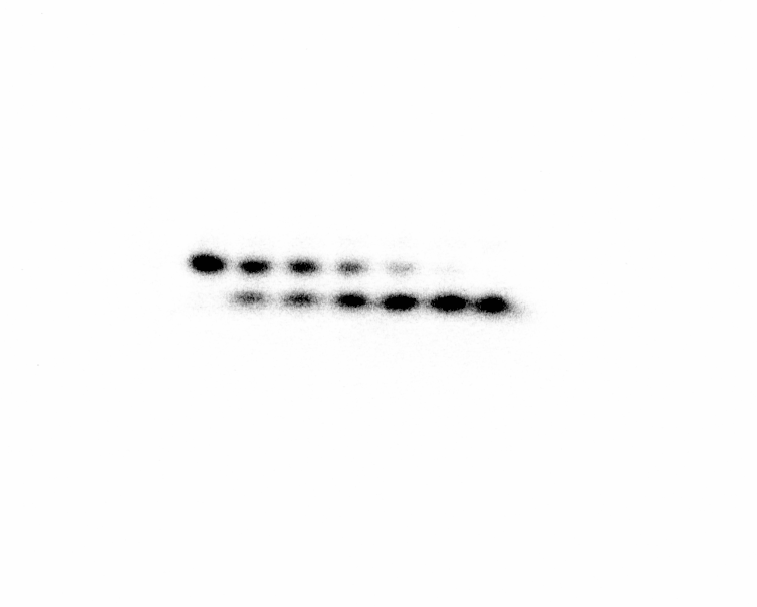

Supplement: Figure 2—source data 1. — Original, unedited images, labeled overview, and quantification of wild-type and mutant NrnC activity using pGG as the substrate from three replicates. [file elife-70146-fig2-data1.zip › Figure2_source_data_1/Figure 2A-source data 2 (H79A).tif]

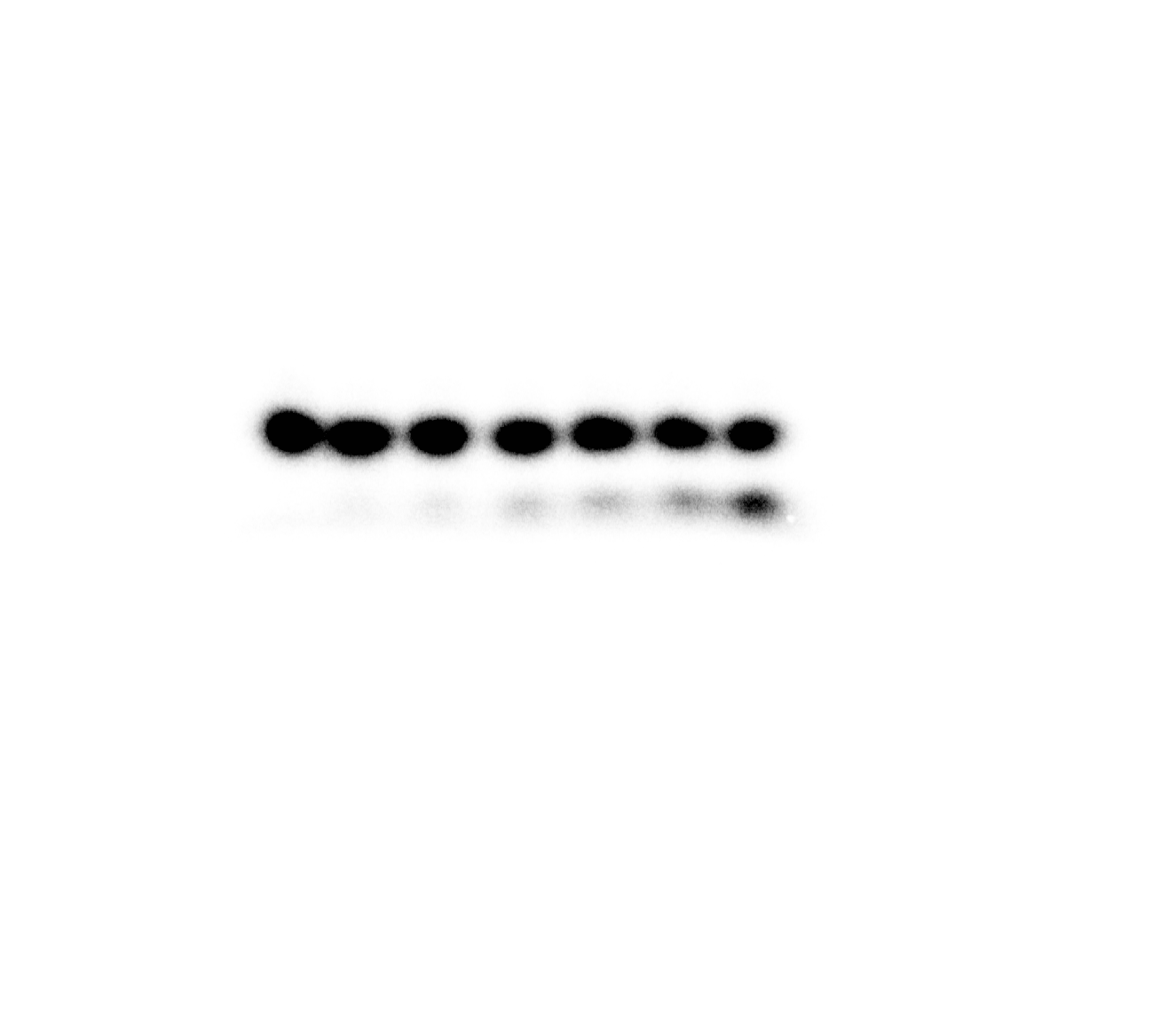

Supplement: Figure 2—source data 1. — Original, unedited images, labeled overview, and quantification of wild-type and mutant NrnC activity using pGG as the substrate from three replicates. [file elife-70146-fig2-data1.zip › Figure2_source_data_1/Figure 2A-source data 3 (K103A).tif]

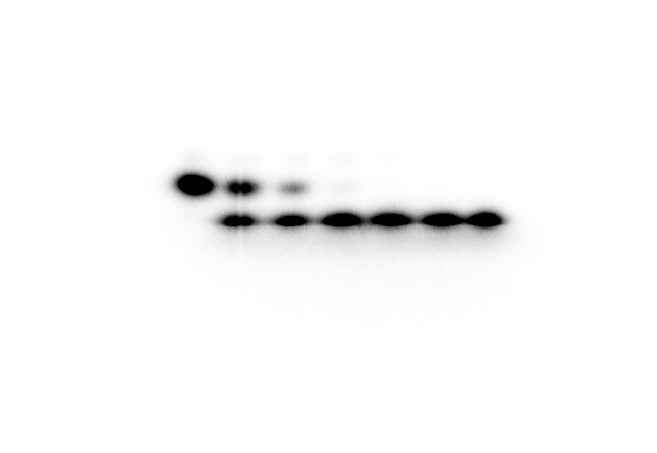

Supplement: Figure 2—source data 1. — Original, unedited images, labeled overview, and quantification of wild-type and mutant NrnC activity using pGG as the substrate from three replicates. [file elife-70146-fig2-data1.zip › Figure2_source_data_1/Figure 2A-source data 1 (WT).tif]

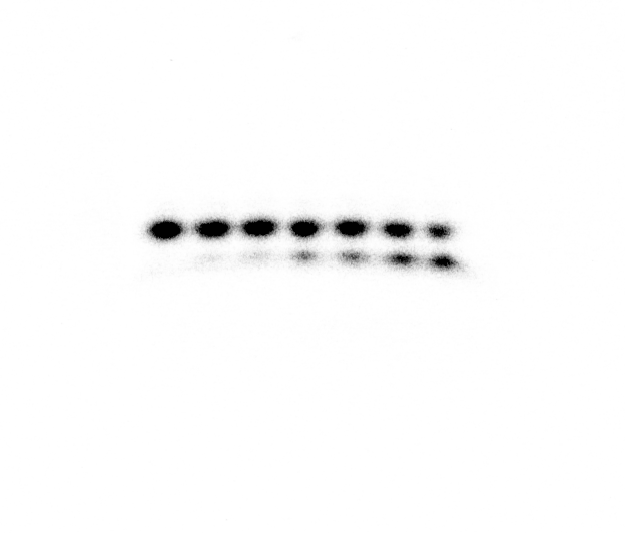

Supplement: Figure 2—source data 1. — Original, unedited images, labeled overview, and quantification of wild-type and mutant NrnC activity using pGG as the substrate from three replicates. [file elife-70146-fig2-data1.zip › Figure2_source_data_1/Figure 2A-source data 1 (K103A).tif]

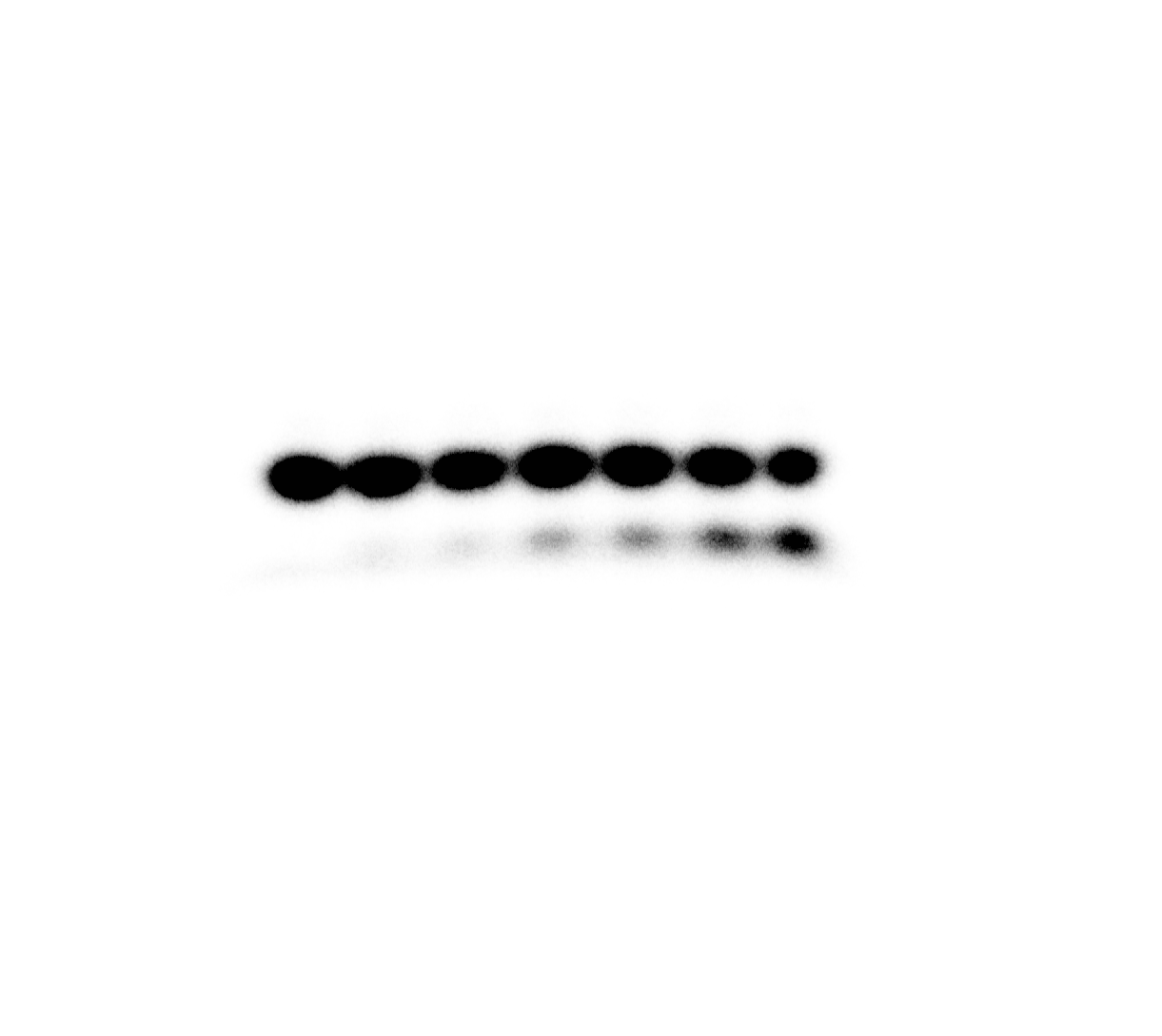

Supplement: Figure 2—source data 1. — Original, unedited images, labeled overview, and quantification of wild-type and mutant NrnC activity using pGG as the substrate from three replicates. [file elife-70146-fig2-data1.zip › Figure2_source_data_1/Figure 2A-source data 3 (L31A).tif]

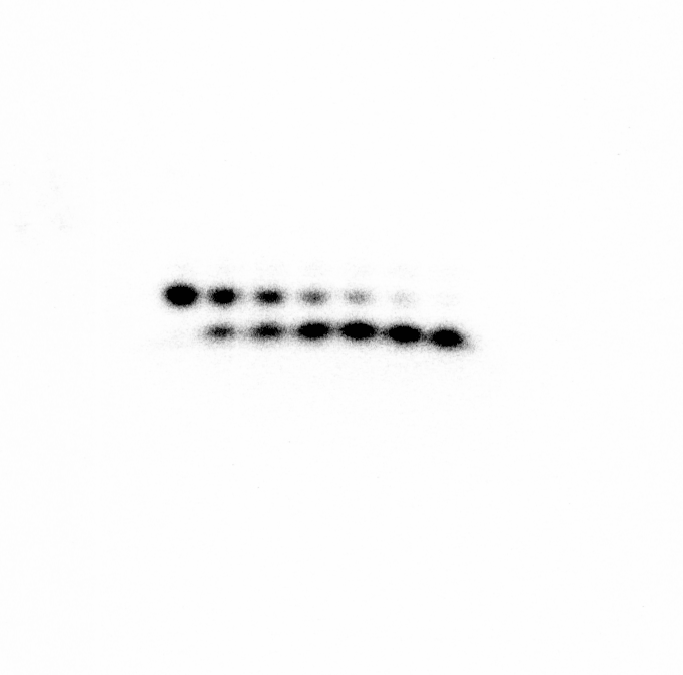

Supplement: Figure 2—source data 1. — Original, unedited images, labeled overview, and quantification of wild-type and mutant NrnC activity using pGG as the substrate from three replicates. [file elife-70146-fig2-data1.zip › Figure2_source_data_1/Figure 2A-source data 3 (H205A).tif]

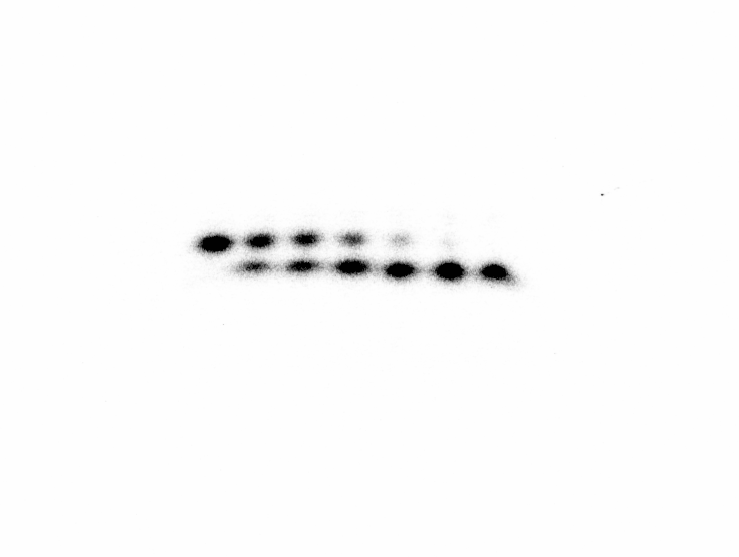

Supplement: Figure 2—source data 1. — Original, unedited images, labeled overview, and quantification of wild-type and mutant NrnC activity using pGG as the substrate from three replicates. [file elife-70146-fig2-data1.zip › Figure2_source_data_1/Figure 2A-source data 1 (H79A).tif]

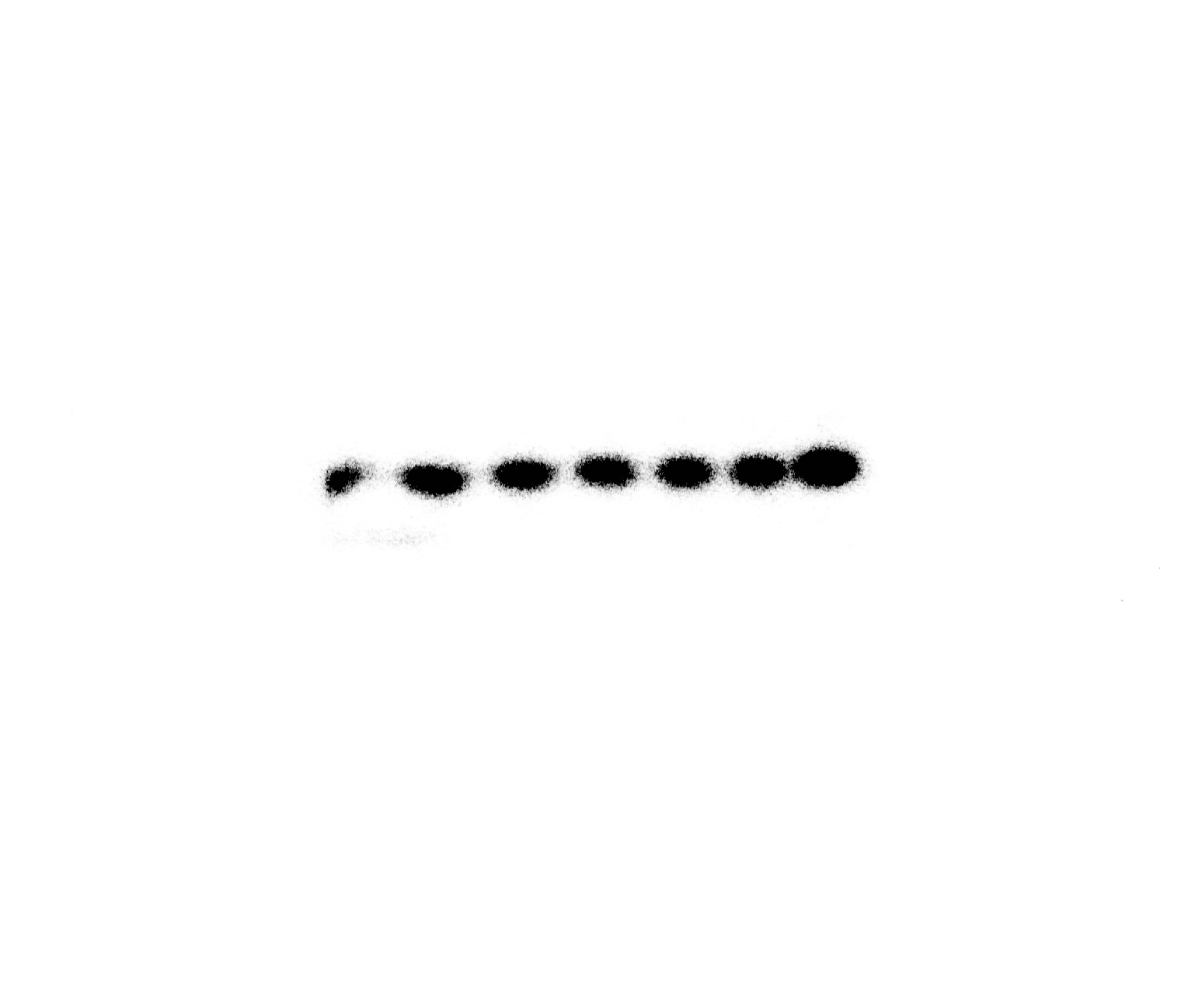

Supplement: Figure 2—source data 1. — Original, unedited images, labeled overview, and quantification of wild-type and mutant NrnC activity using pGG as the substrate from three replicates. [file elife-70146-fig2-data1.zip › Figure2_source_data_1/Figure 2A-source data 2 (D25A).tif]

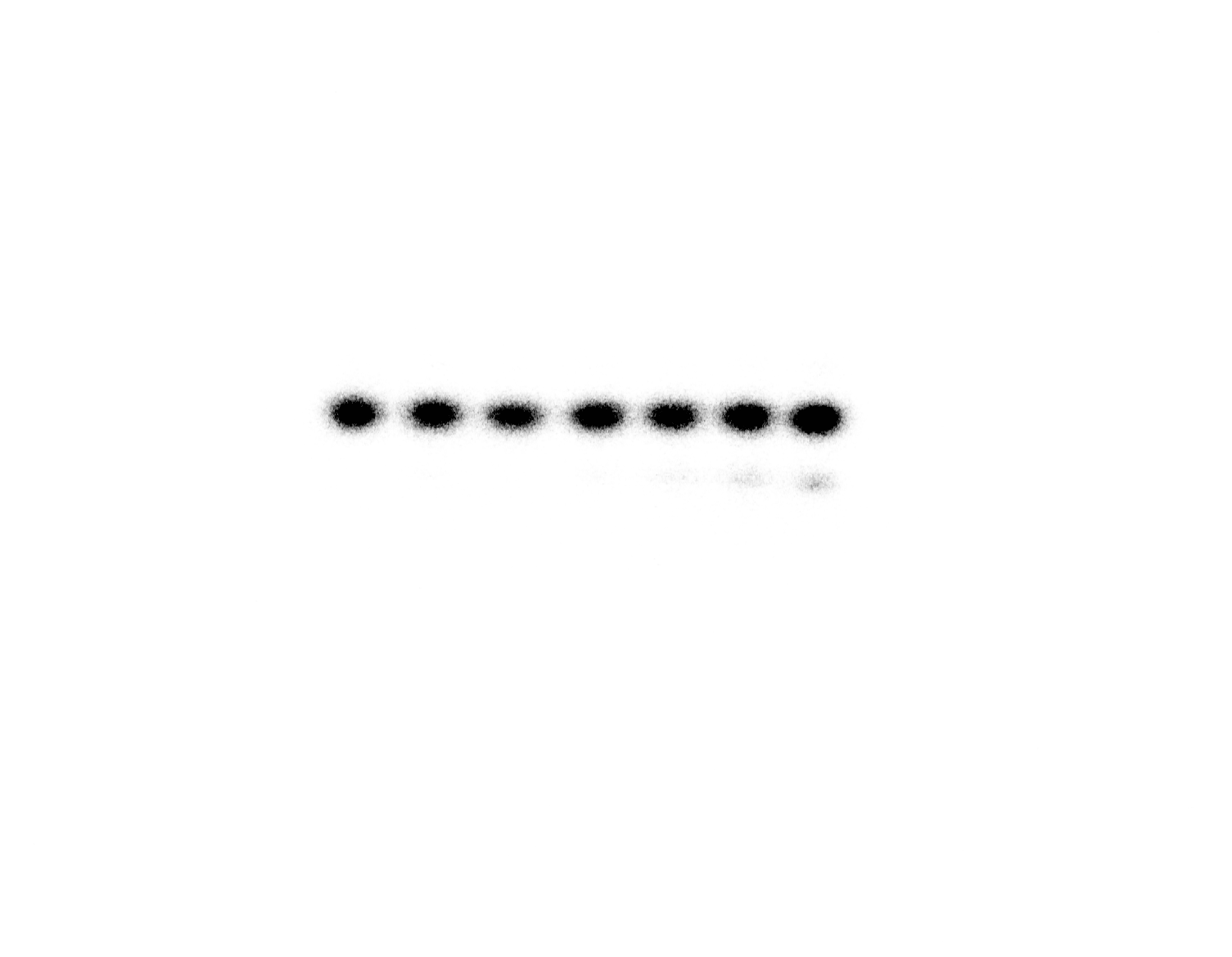

Supplement: Figure 2—source data 1. — Original, unedited images, labeled overview, and quantification of wild-type and mutant NrnC activity using pGG as the substrate from three replicates. [file elife-70146-fig2-data1.zip › Figure2_source_data_1/Figure 2A-source data 1 (L31A).tif]

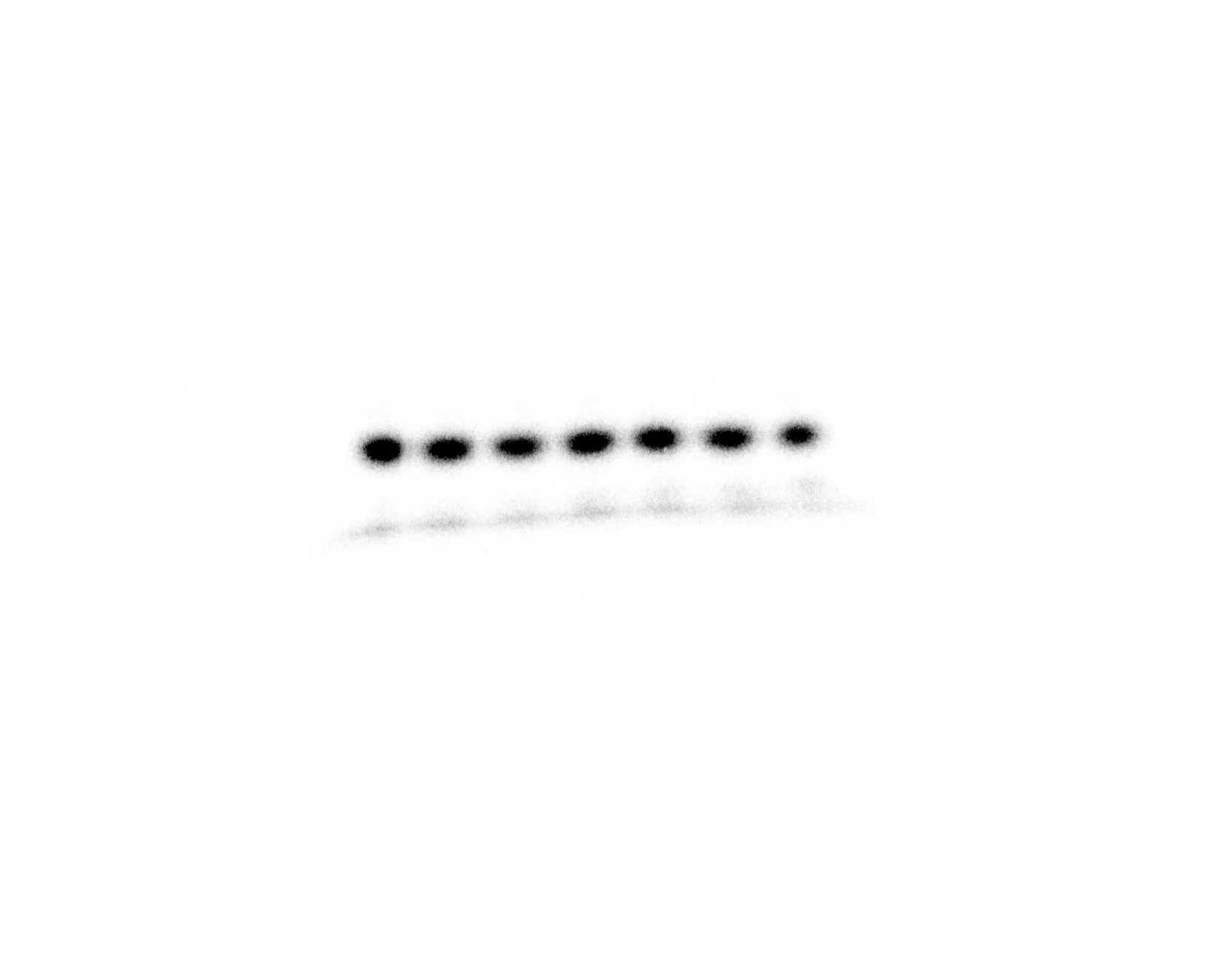

Supplement: Figure 2—source data 1. — Original, unedited images, labeled overview, and quantification of wild-type and mutant NrnC activity using pGG as the substrate from three replicates. [file elife-70146-fig2-data1.zip › Figure2_source_data_1/Figure 2A-source data 2 (K103A).tif]

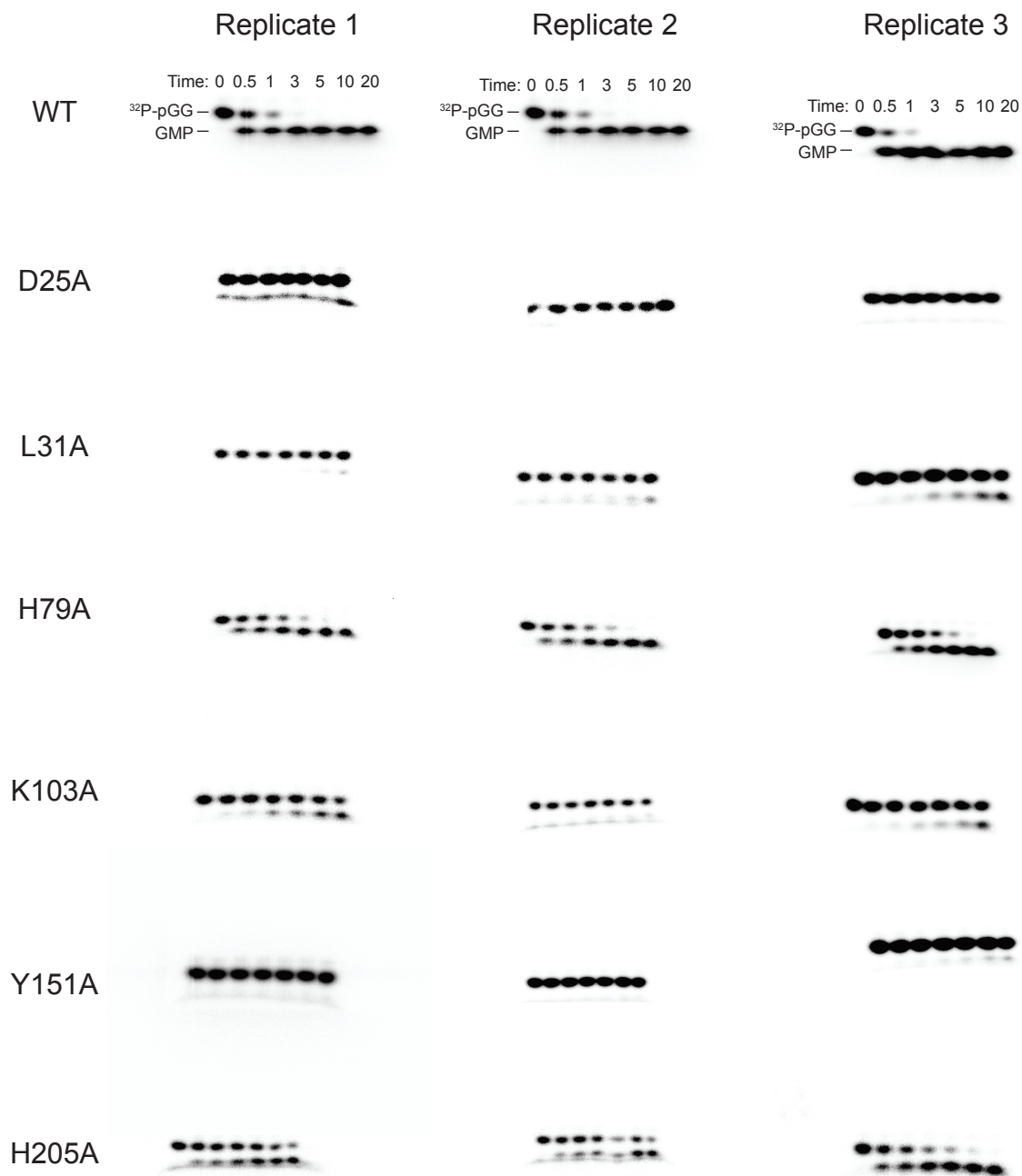

Supplement: Figure 2—source data 1. — Original, unedited images, labeled overview, and quantification of wild-type and mutant NrnC activity using pGG as the substrate from three replicates. [file elife-70146-fig2-data1.zip › Figure2_source_data_1/Figure 2A triplicate.pdf]

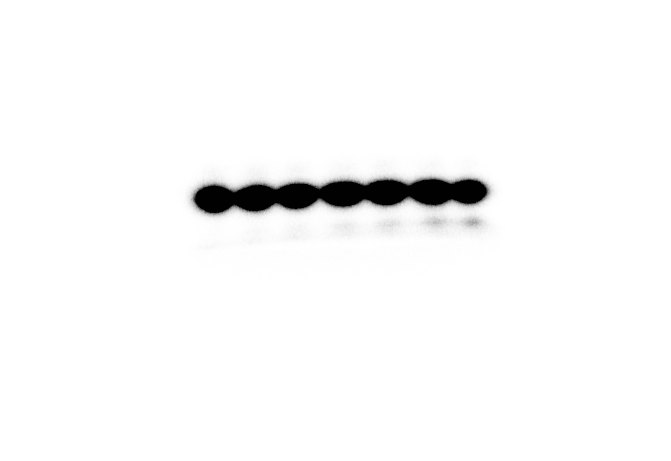

Supplement: Figure 2—source data 1. — Original, unedited images, labeled overview, and quantification of wild-type and mutant NrnC activity using pGG as the substrate from three replicates. [file elife-70146-fig2-data1.zip › Figure2_source_data_1/Figure 2A-source data 3 (Y151A).tif]

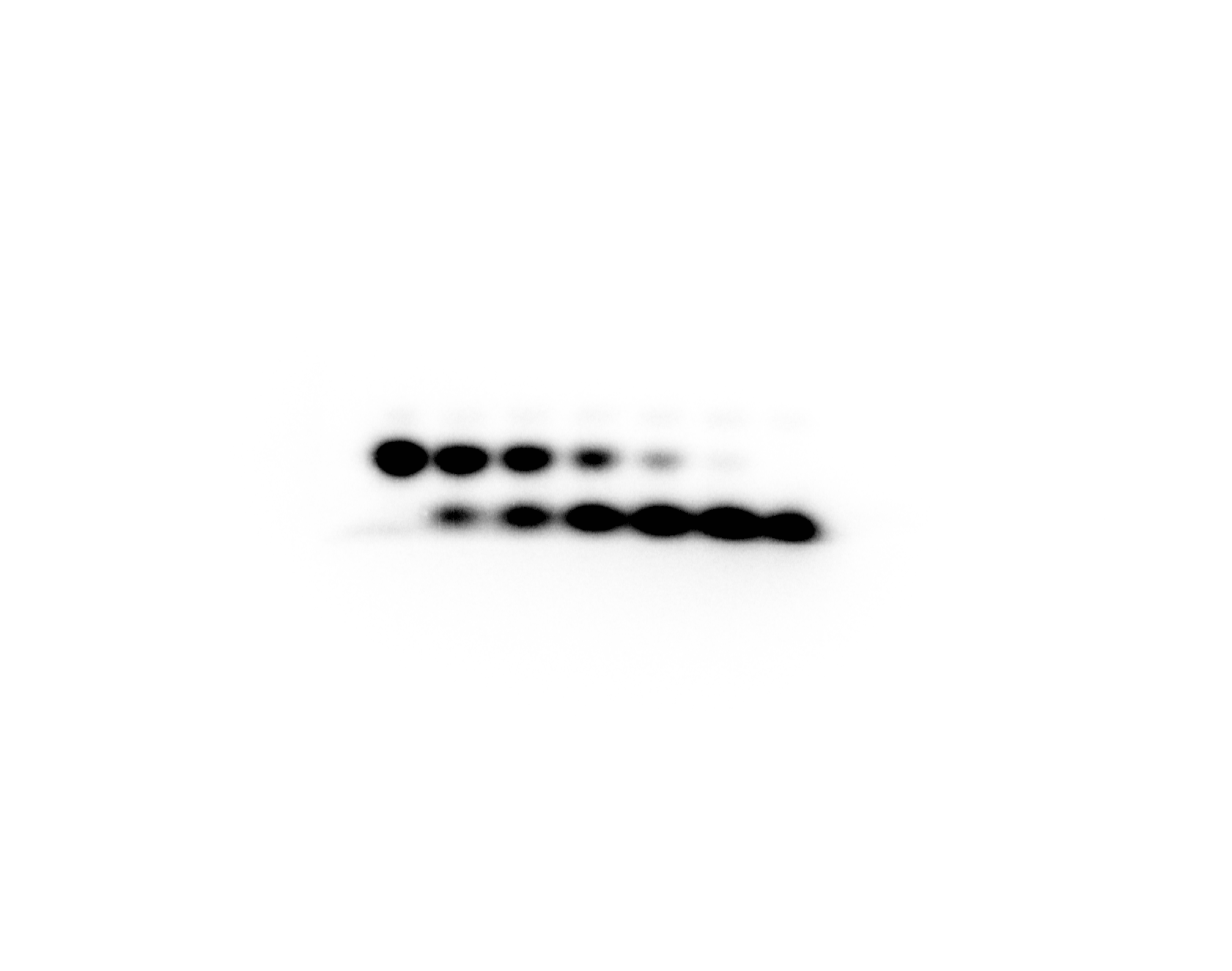

Supplement: Figure 2—source data 1. — Original, unedited images, labeled overview, and quantification of wild-type and mutant NrnC activity using pGG as the substrate from three replicates. [file elife-70146-fig2-data1.zip › Figure2_source_data_1/Figure 2A-source data 3 (H79A).tif]

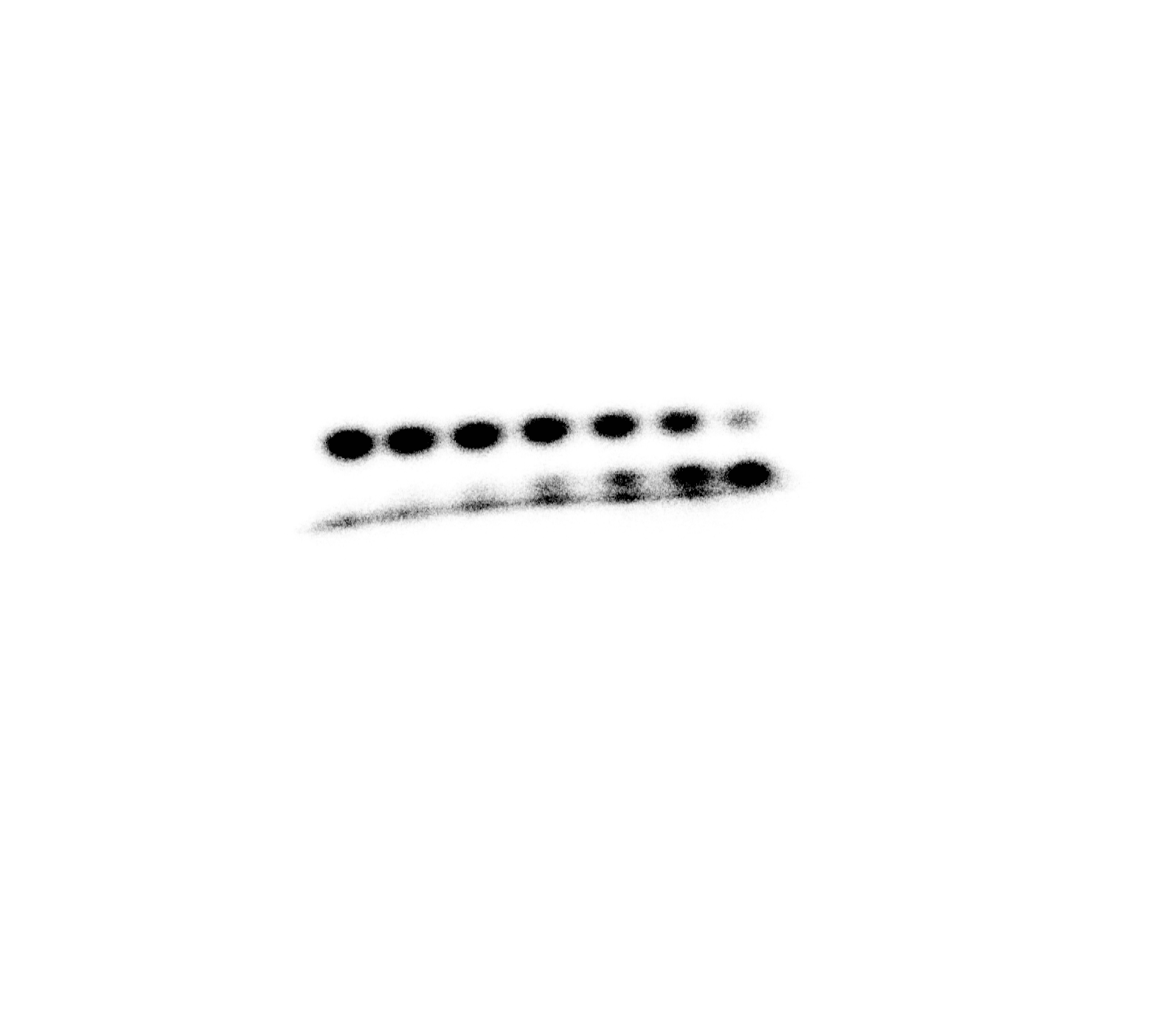

Supplement: Figure 2—source data 2. — Original, unedited images, labeled overview, and quantification of nano-RNase C (NrnC) activity using pGG as the substrate in the presence or absence of GG or pAp from three replicates. [file elife-70146-fig2-data2.zip › Figure2_source_data_2/Figure 2B-source data 3 (pAp).tif]

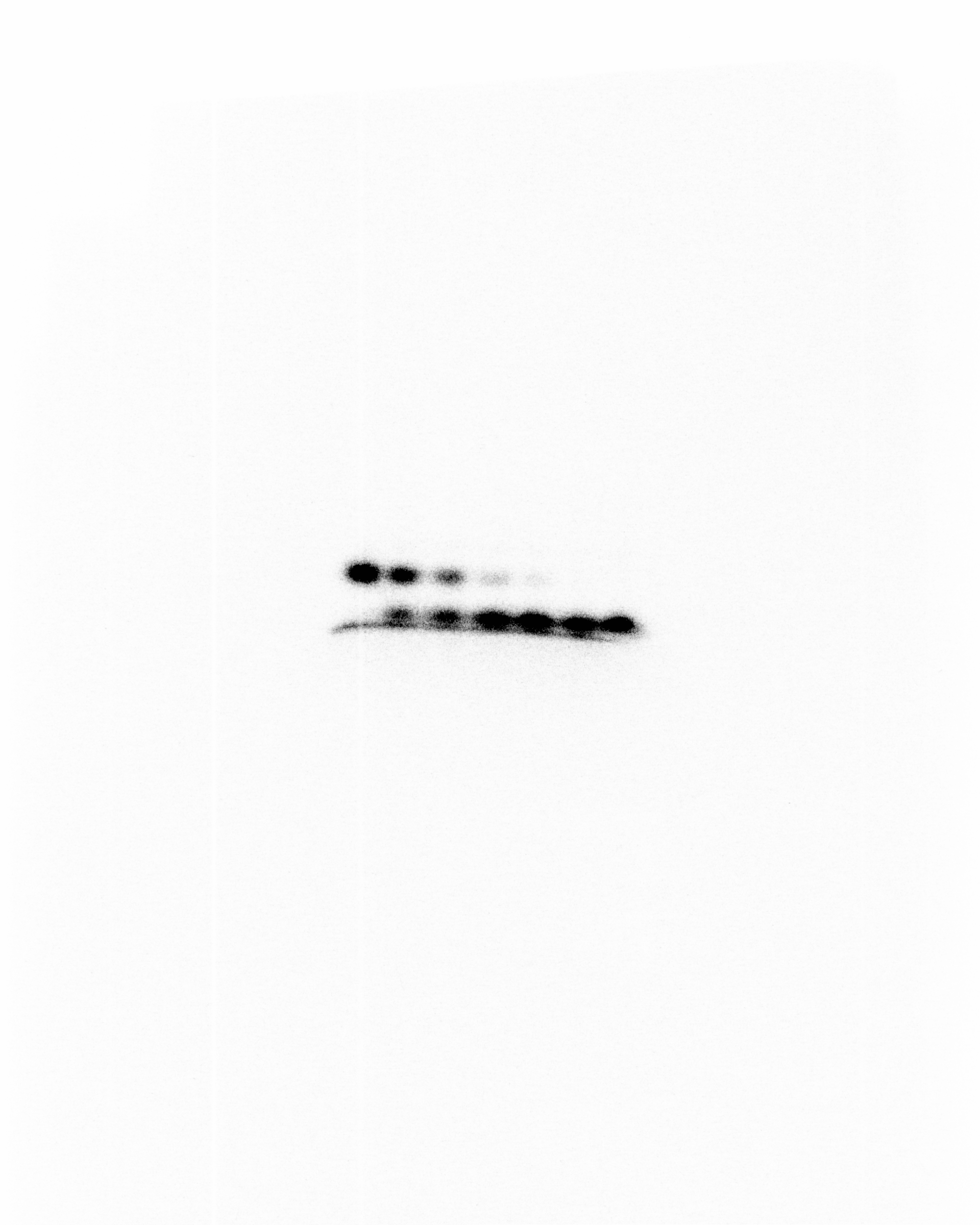

Supplement: Figure 2—source data 2. — Original, unedited images, labeled overview, and quantification of nano-RNase C (NrnC) activity using pGG as the substrate in the presence or absence of GG or pAp from three replicates. [file elife-70146-fig2-data2.zip › Figure2_source_data_2/Figure 2B-source data 3 (WT).tif]

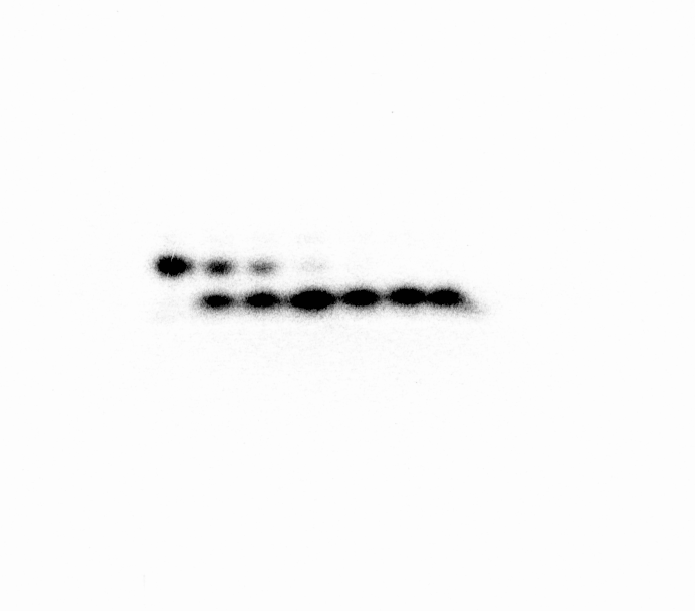

Supplement: Figure 2—source data 2. — Original, unedited images, labeled overview, and quantification of nano-RNase C (NrnC) activity using pGG as the substrate in the presence or absence of GG or pAp from three replicates. [file elife-70146-fig2-data2.zip › Figure2_source_data_2/Figure 2B-source data 2 (WT).tif]

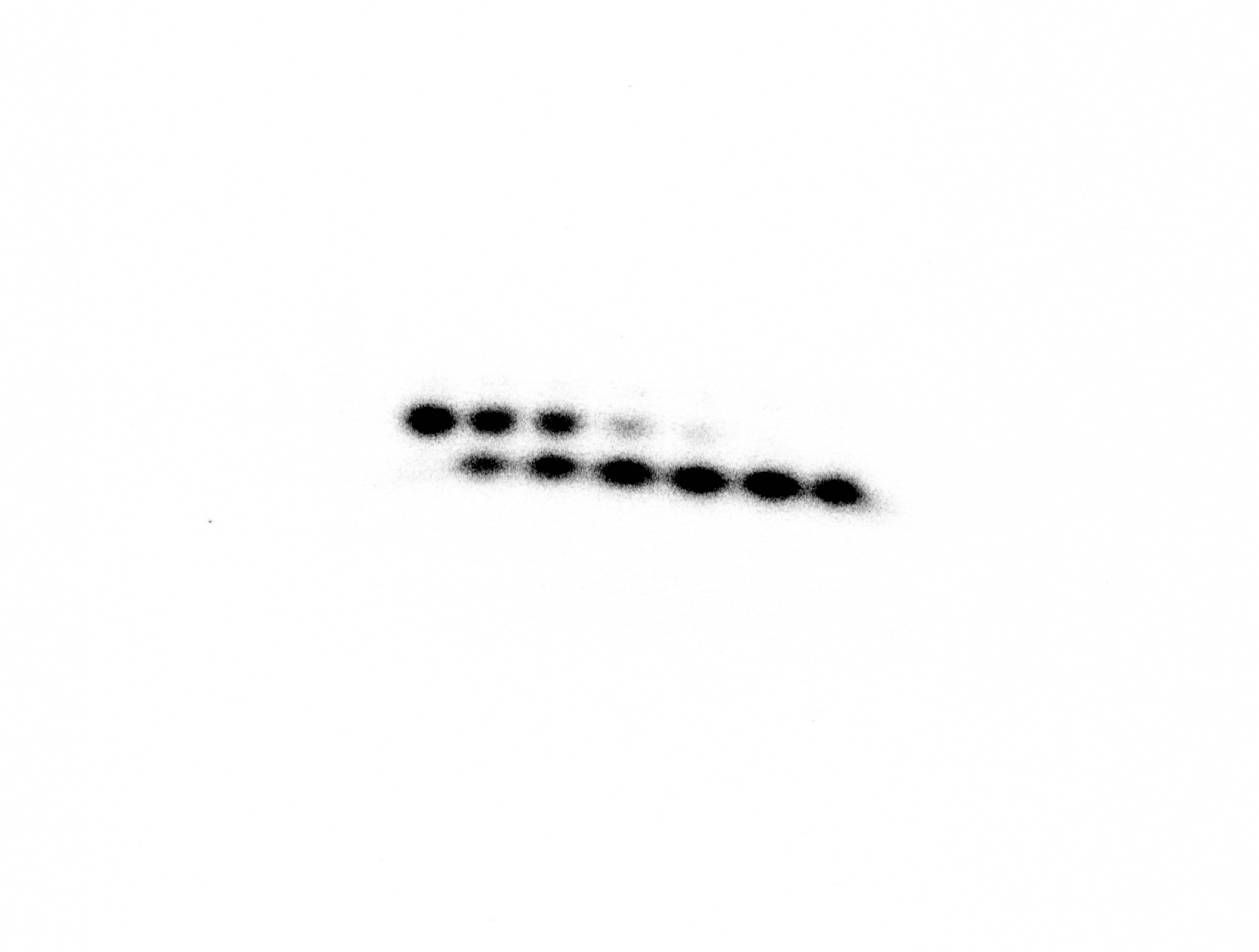

Supplement: Figure 2—source data 2. — Original, unedited images, labeled overview, and quantification of nano-RNase C (NrnC) activity using pGG as the substrate in the presence or absence of GG or pAp from three replicates. [file elife-70146-fig2-data2.zip › Figure2_source_data_2/Figure 2B-source data 1 (GpG).tif]

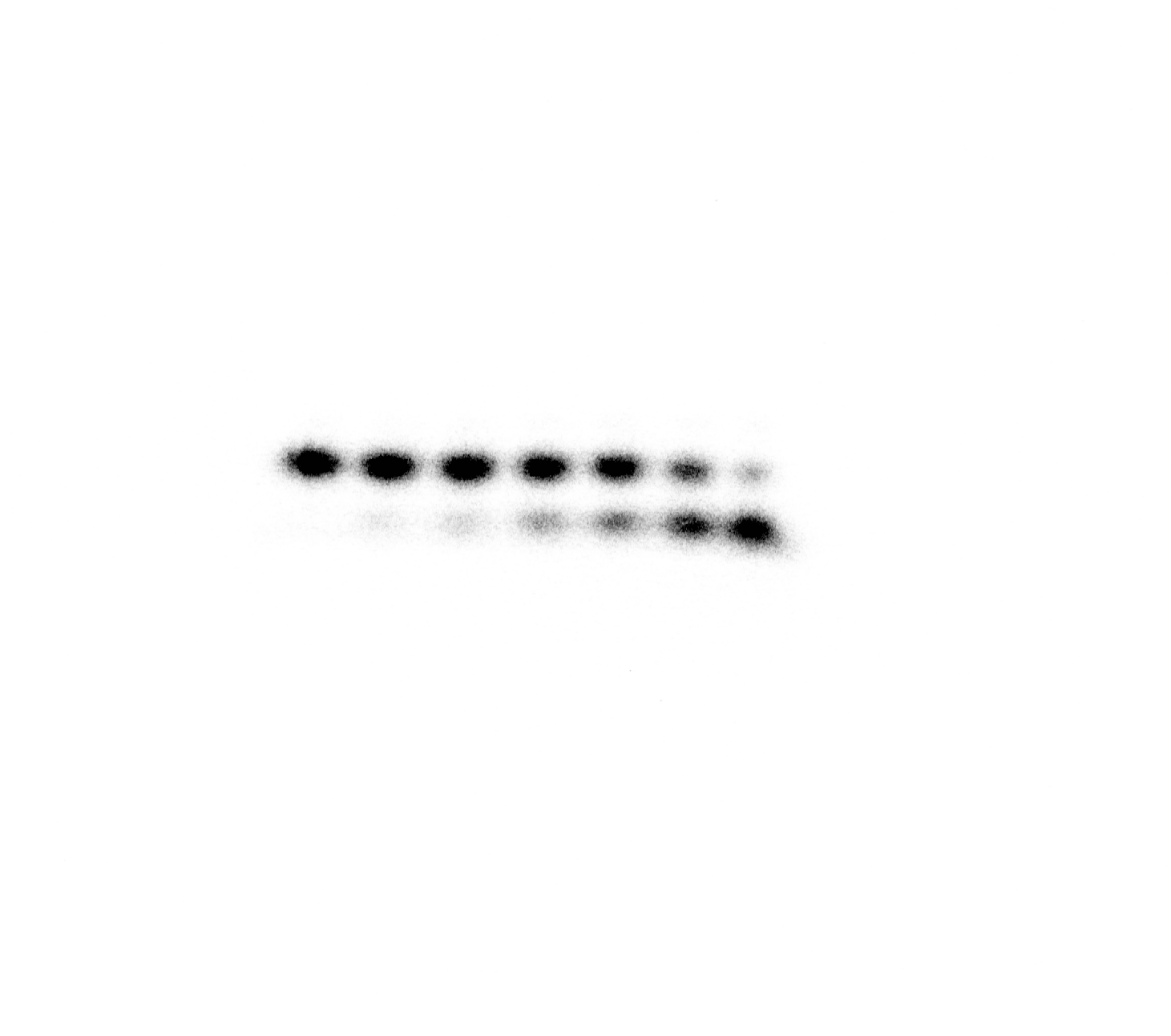

Supplement: Figure 2—source data 2. — Original, unedited images, labeled overview, and quantification of nano-RNase C (NrnC) activity using pGG as the substrate in the presence or absence of GG or pAp from three replicates. [file elife-70146-fig2-data2.zip › Figure2_source_data_2/Figure 2B-source data 2 (pAp).tif]

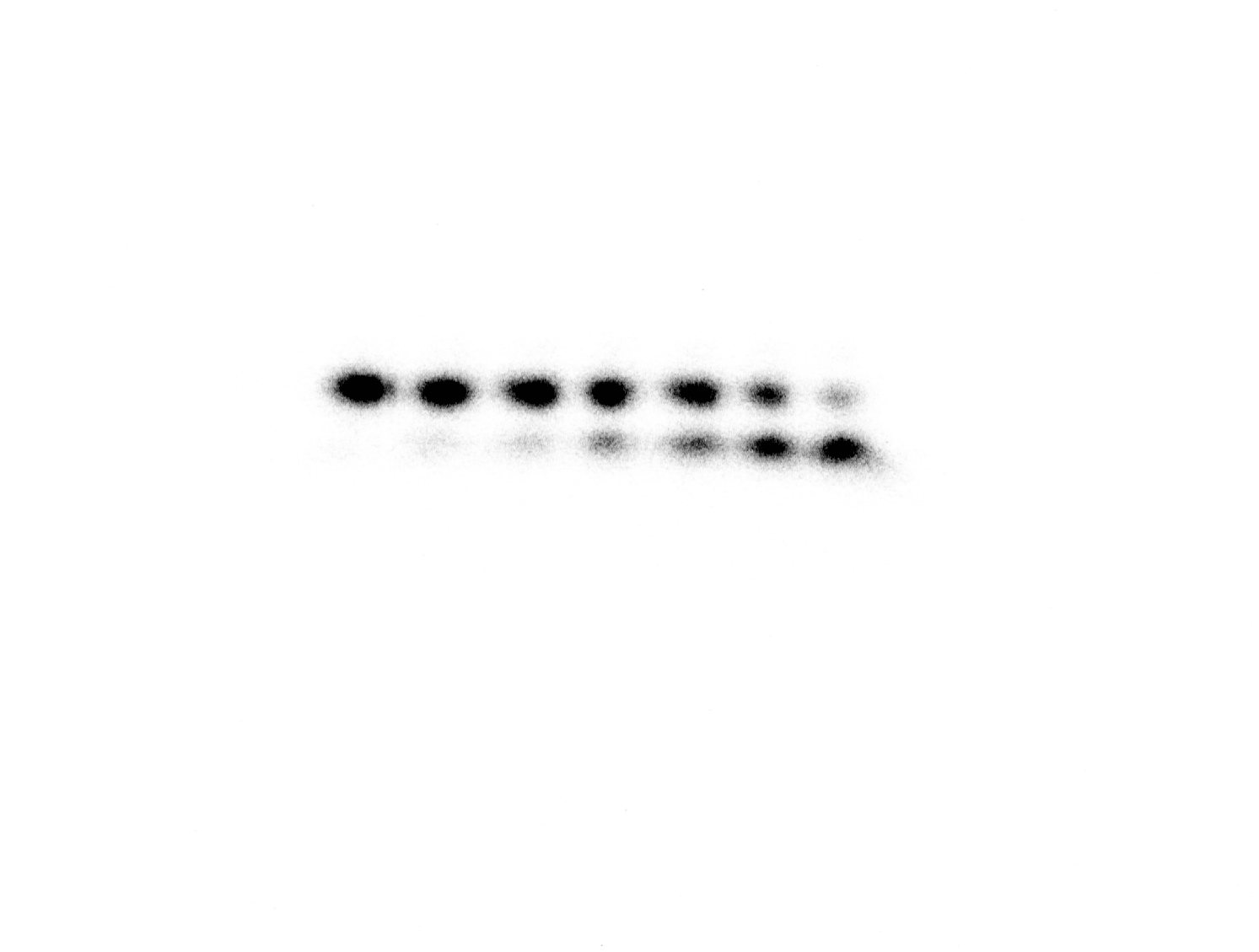

Supplement: Figure 2—source data 2. — Original, unedited images, labeled overview, and quantification of nano-RNase C (NrnC) activity using pGG as the substrate in the presence or absence of GG or pAp from three replicates. [file elife-70146-fig2-data2.zip › Figure2_source_data_2/Figure 2B-source data 1 (pAp).tif]

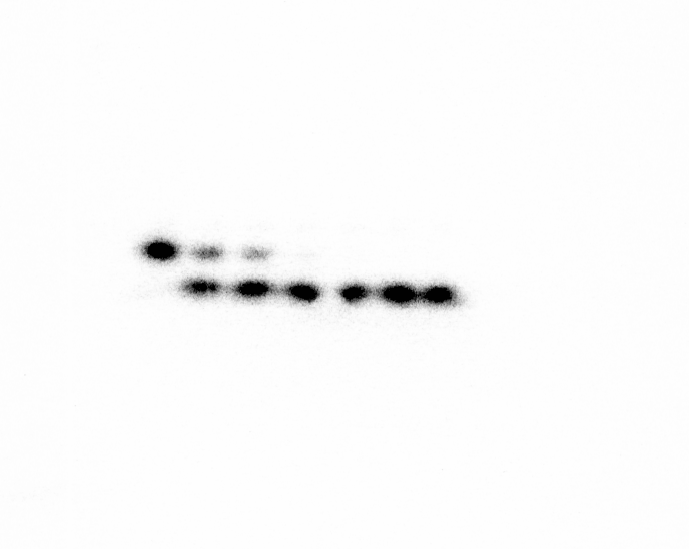

Supplement: Figure 2—source data 2. — Original, unedited images, labeled overview, and quantification of nano-RNase C (NrnC) activity using pGG as the substrate in the presence or absence of GG or pAp from three replicates. [file elife-70146-fig2-data2.zip › Figure2_source_data_2/Figure 2B-source data 1 (WT).tif]

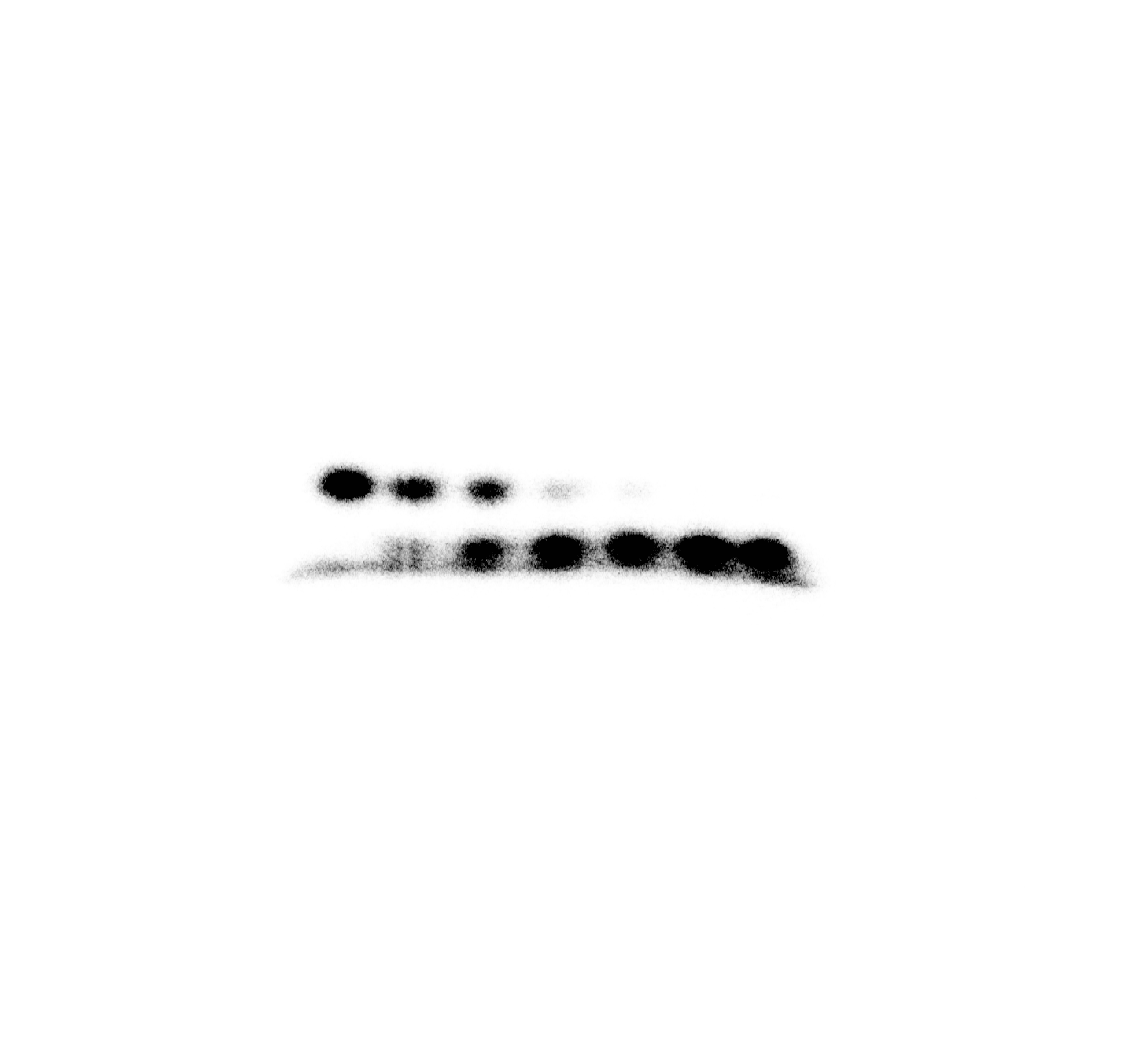

Supplement: Figure 2—source data 2. — Original, unedited images, labeled overview, and quantification of nano-RNase C (NrnC) activity using pGG as the substrate in the presence or absence of GG or pAp from three replicates. [file elife-70146-fig2-data2.zip › Figure2_source_data_2/Figure 2B-source data 3 (GpG).tif]

Replicate 1

Replicate 2

Replicate 3

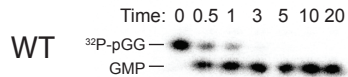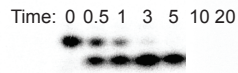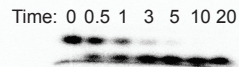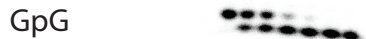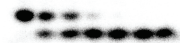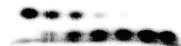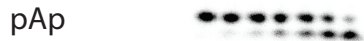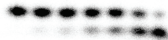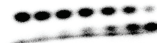

Supplement: Figure 2—source data 2. — Original, unedited images, labeled overview, and quantification of nano-RNase C (NrnC) activity using pGG as the substrate in the presence or absence of GG or pAp from three replicates. [file elife-70146-fig2-data2.zip › Figure2_source_data_2/Figure 2B Triplicate.pdf]

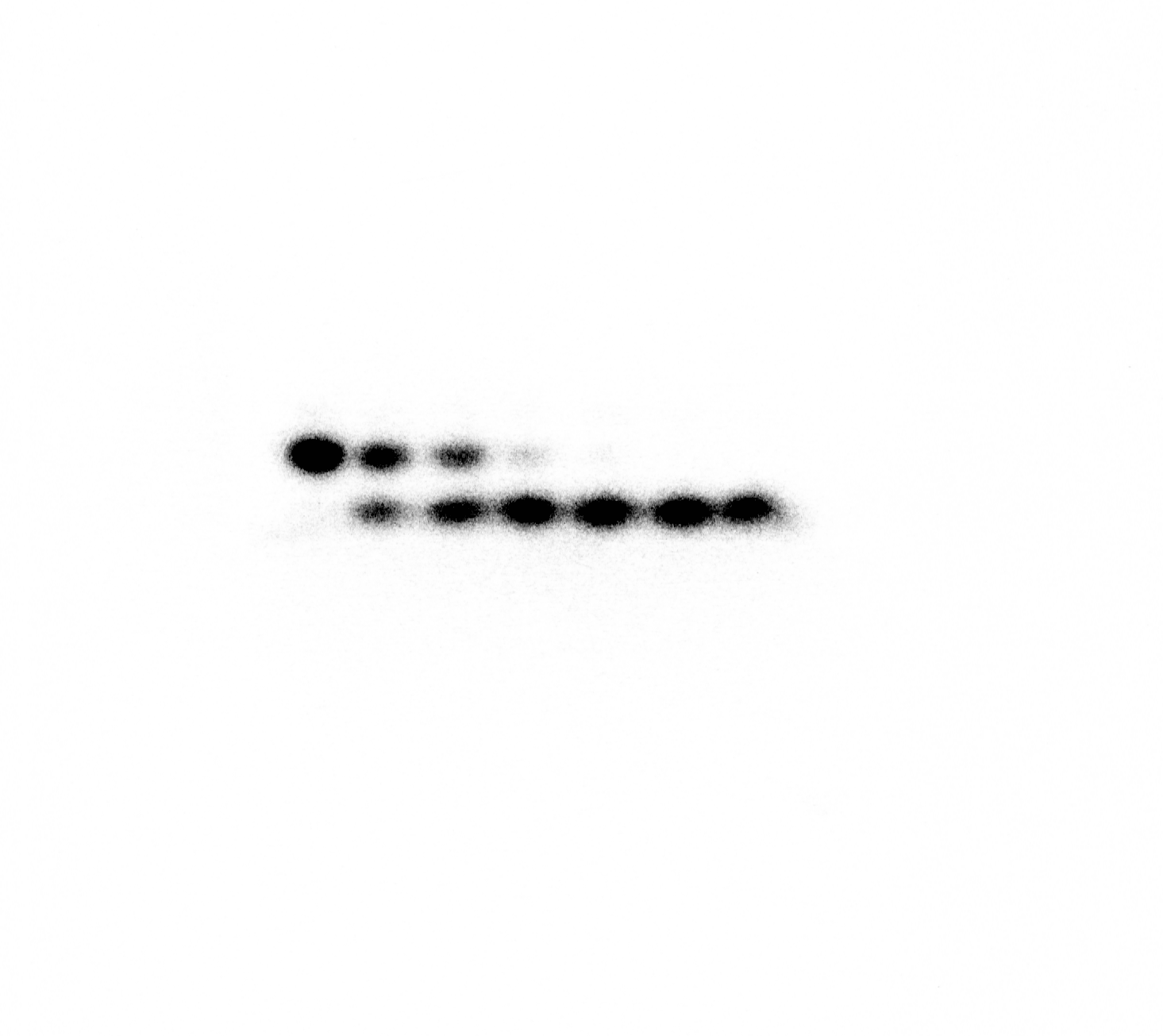

Supplement: Figure 2—source data 2. — Original, unedited images, labeled overview, and quantification of nano-RNase C (NrnC) activity using pGG as the substrate in the presence or absence of GG or pAp from three replicates. [file elife-70146-fig2-data2.zip › Figure2_source_data_2/Figure 2B-source data 2 (GpG).tif]

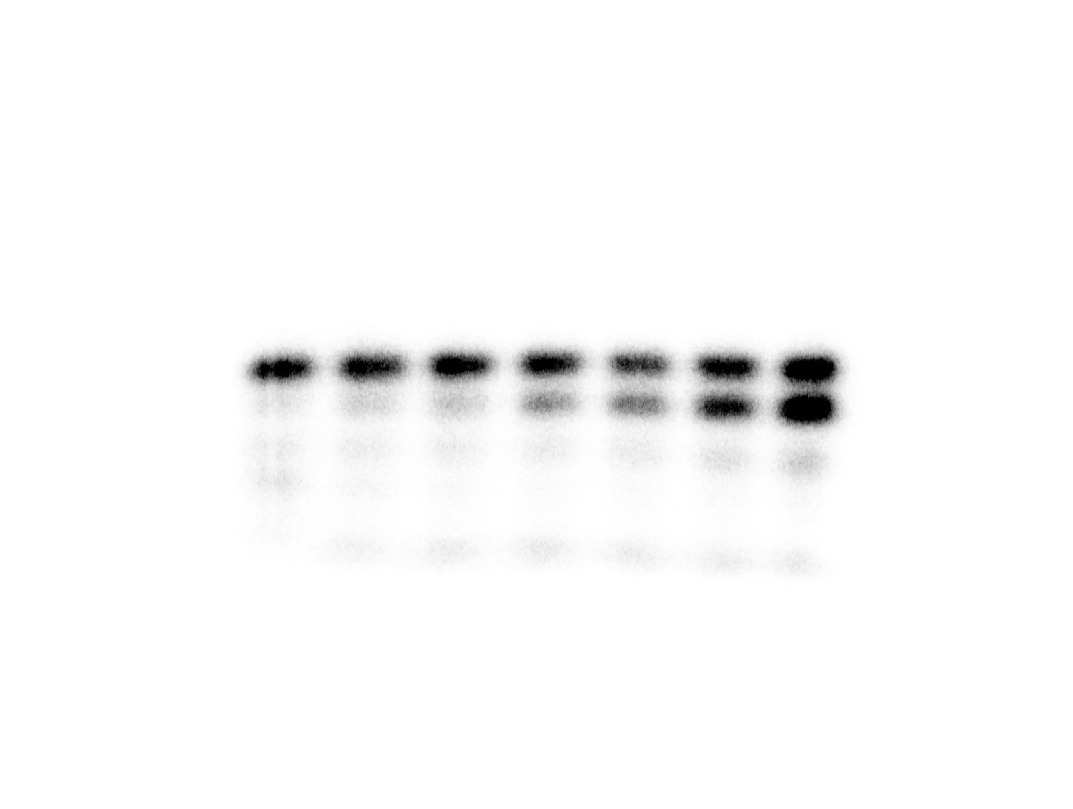

Supplement: Figure 5—source data 1. — Original, unedited images and labeled composite overview of nano-RNase C (NrnC) activity against substrates with different length in three replicates. [file elife-70146-fig5-data1.zip › Figure5_source_data_1/Figure 5A-source data 3 (pAAGGGG).tif]

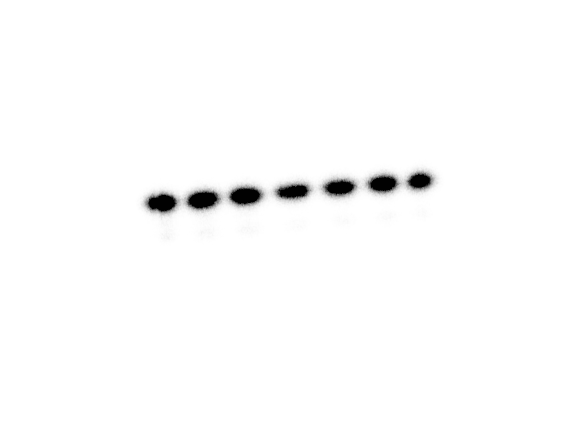

Supplement: Figure 5—source data 1. — Original, unedited images and labeled composite overview of nano-RNase C (NrnC) activity against substrates with different length in three replicates. [file elife-70146-fig5-data1.zip › Figure5_source_data_1/Figure 5A-source data 1 (pAGG).tif]

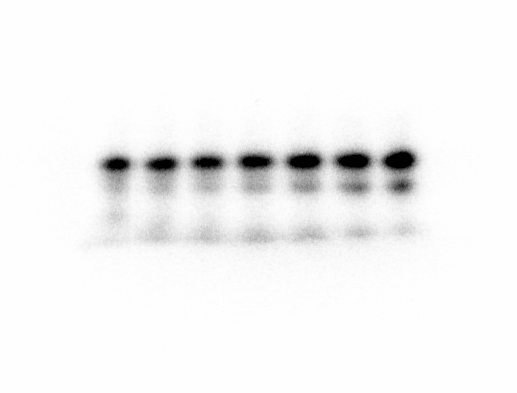

Supplement: Figure 5—source data 1. — Original, unedited images and labeled composite overview of nano-RNase C (NrnC) activity against substrates with different length in three replicates. [file elife-70146-fig5-data1.zip › Figure5_source_data_1/Figure 5A-source data 1 (pAAGG).tif]

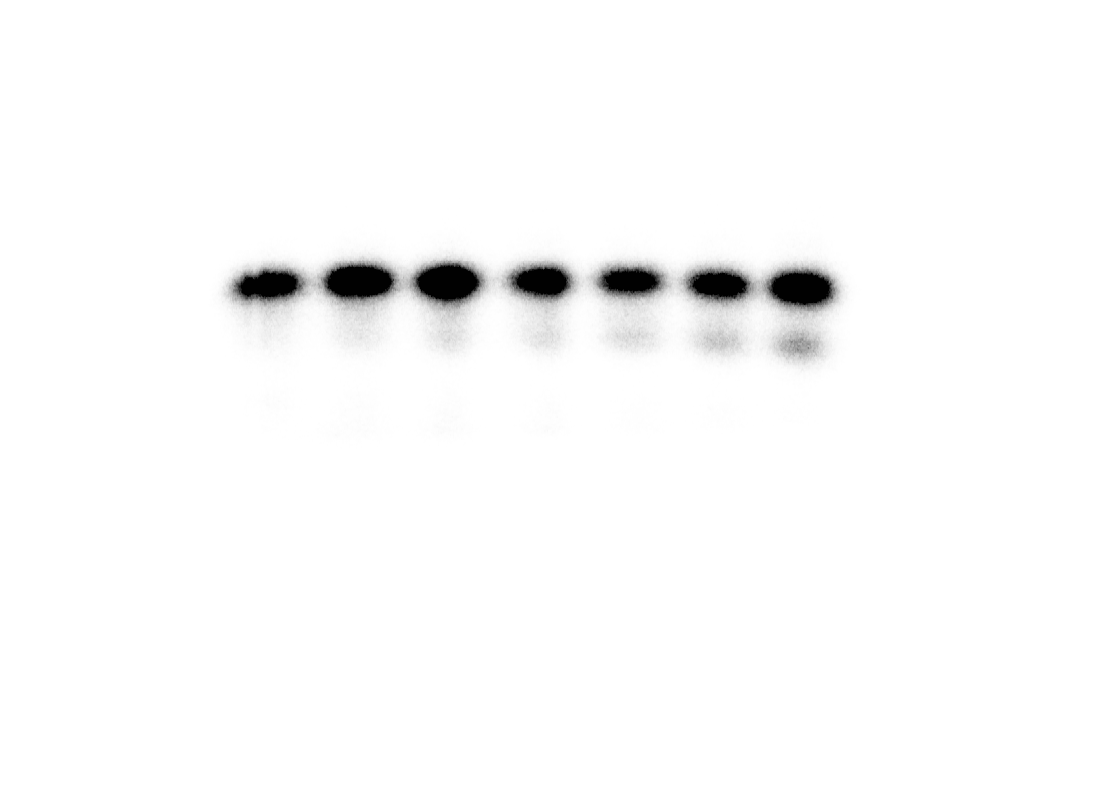

Supplement: Figure 5—source data 1. — Original, unedited images and labeled composite overview of nano-RNase C (NrnC) activity against substrates with different length in three replicates. [file elife-70146-fig5-data1.zip › Figure5_source_data_1/Figure 5A-source data 2 (pAAGG).tif]

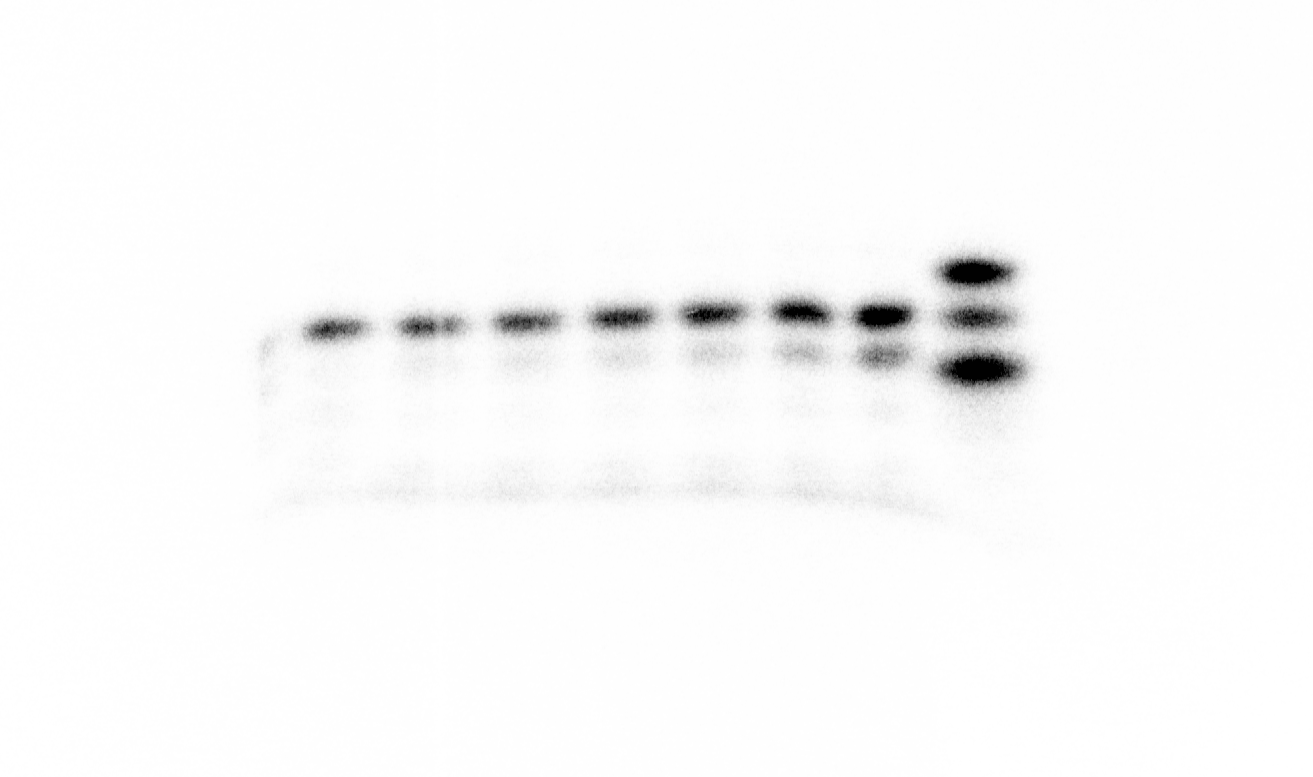

Supplement: Figure 5—source data 1. — Original, unedited images and labeled composite overview of nano-RNase C (NrnC) activity against substrates with different length in three replicates. [file elife-70146-fig5-data1.zip › Figure5_source_data_1/Figure 5A-source data 3 (pAAGGG).tif]

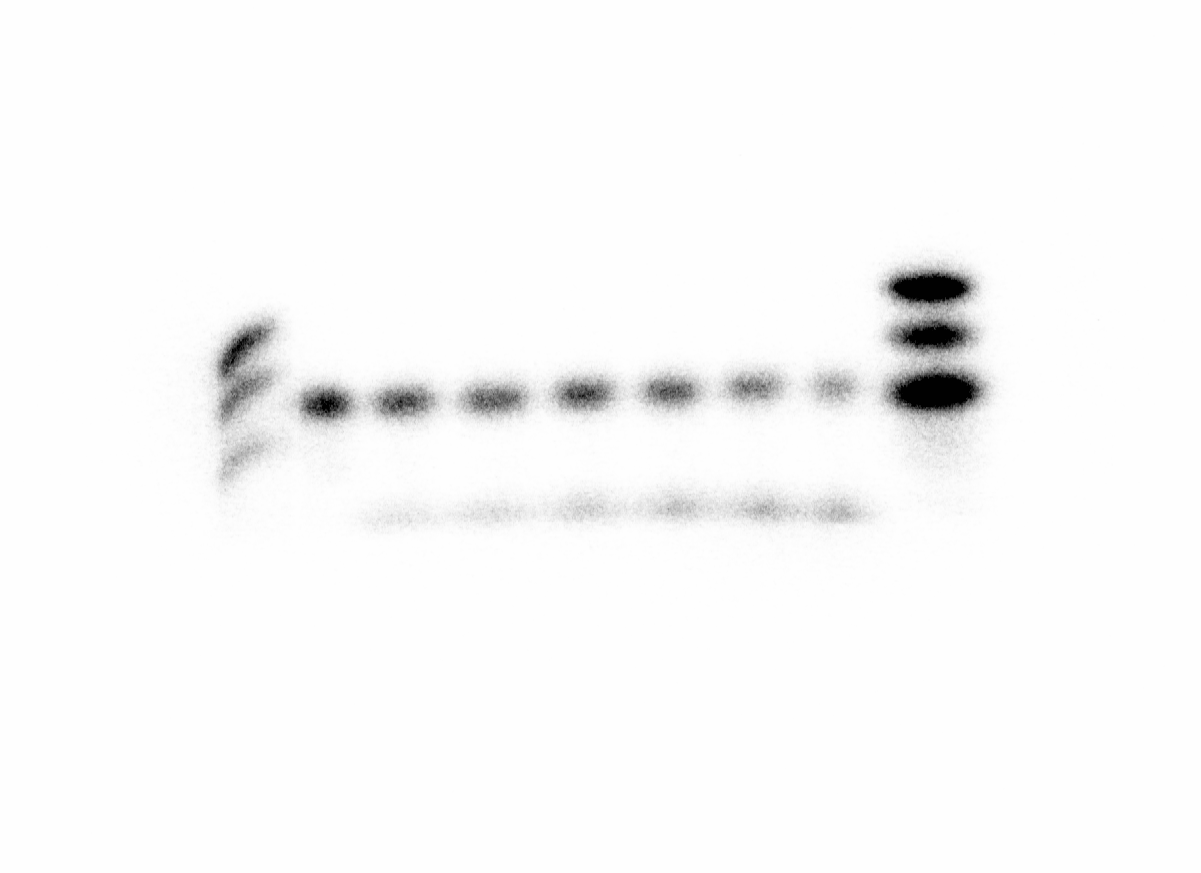

Supplement: Figure 5—source data 1. — Original, unedited images and labeled composite overview of nano-RNase C (NrnC) activity against substrates with different length in three replicates. [file elife-70146-fig5-data1.zip › Figure5_source_data_1/Figure 5A-source data 3 (pAGG).tif]

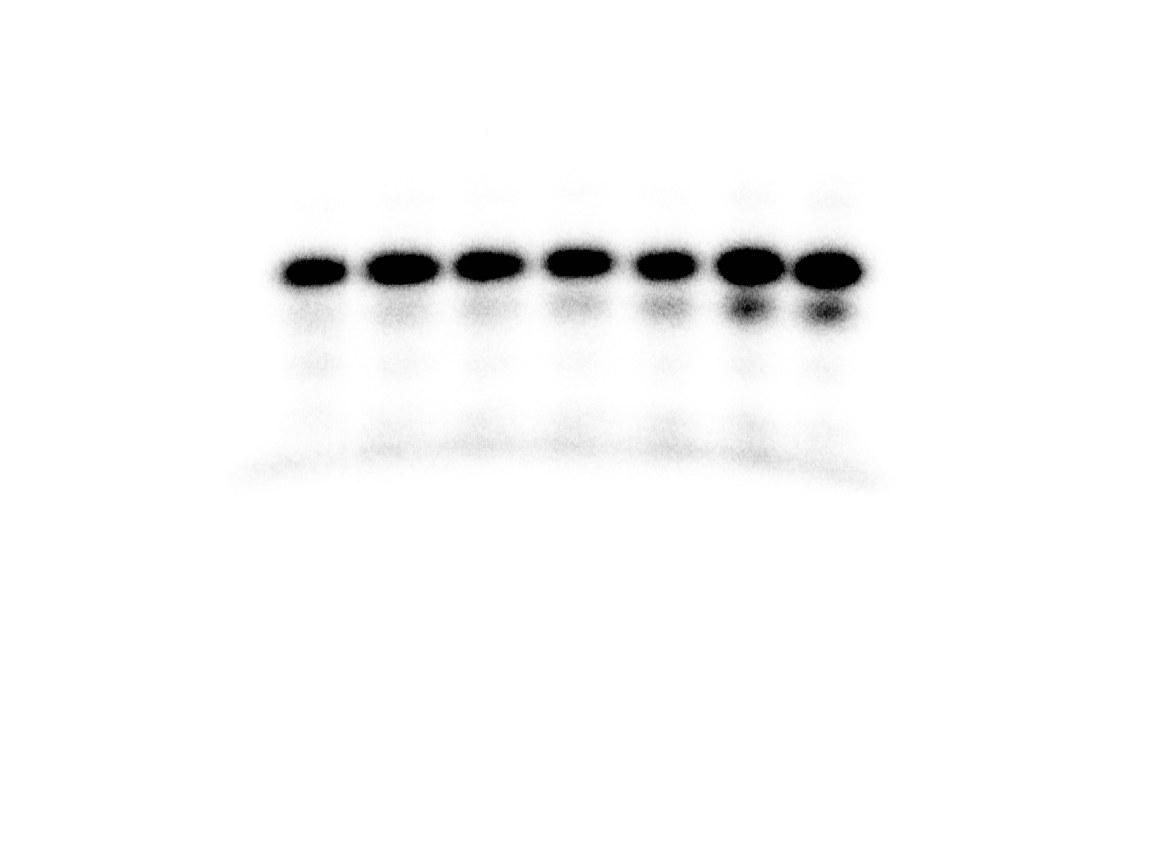

Supplement: Figure 5—source data 1. — Original, unedited images and labeled composite overview of nano-RNase C (NrnC) activity against substrates with different length in three replicates. [file elife-70146-fig5-data1.zip › Figure5_source_data_1/Figure 5A-source data 2 (pAAGGG).tif]

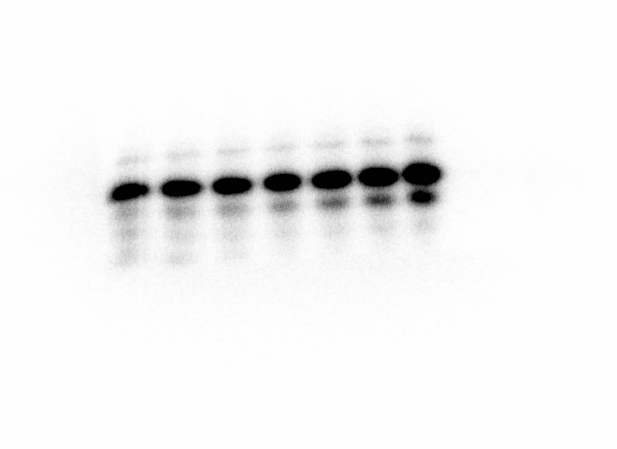

Supplement: Figure 5—source data 1. — Original, unedited images and labeled composite overview of nano-RNase C (NrnC) activity against substrates with different length in three replicates. [file elife-70146-fig5-data1.zip › Figure5_source_data_1/Figure 5A-source data 1 (pAAGGG).tif]

## Replicate 1

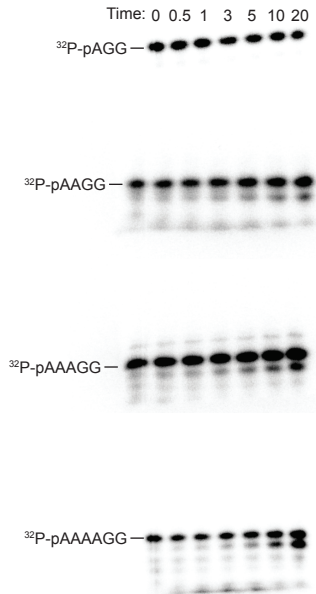

## Replicate 2

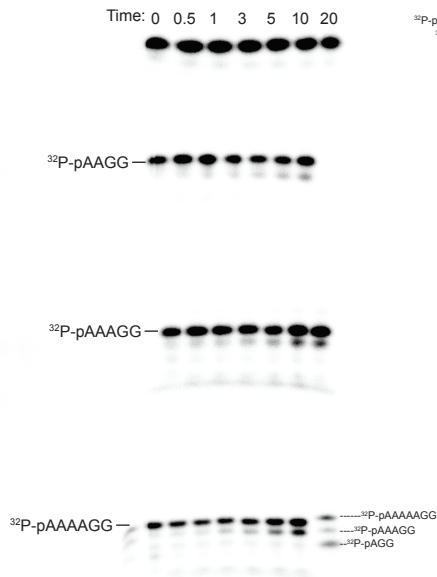

## Replicate 3

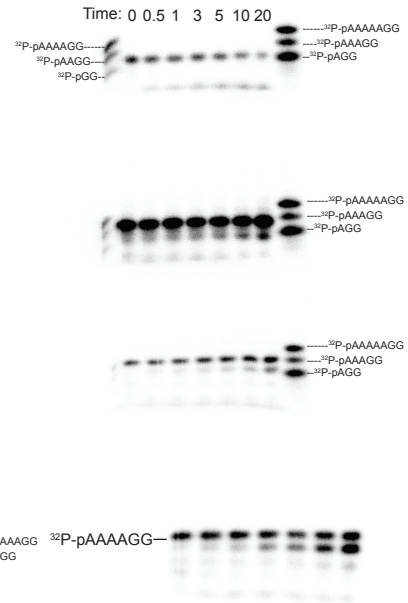

Supplement: Figure 5—source data 1. — Original, unedited images and labeled composite overview of nano-RNase C (NrnC) activity against substrates with different length in three replicates. [file elife-70146-fig5-data1.zip › Figure5_source_data_1/Figure 5A Triplicate.pdf]

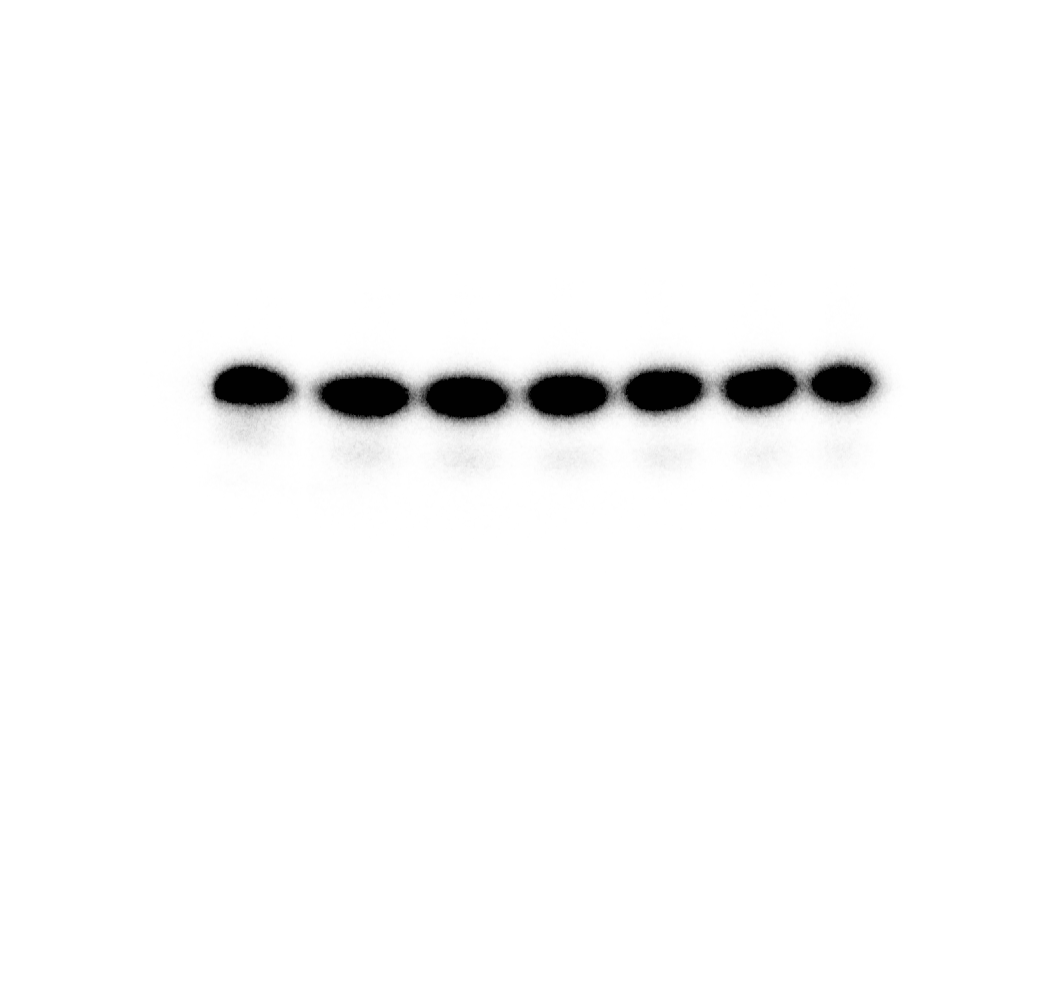

Supplement: Figure 5—source data 1. — Original, unedited images and labeled composite overview of nano-RNase C (NrnC) activity against substrates with different length in three replicates. [file elife-70146-fig5-data1.zip › Figure5_source_data_1/Figure 5A-source data 2 (pAGG).tif]

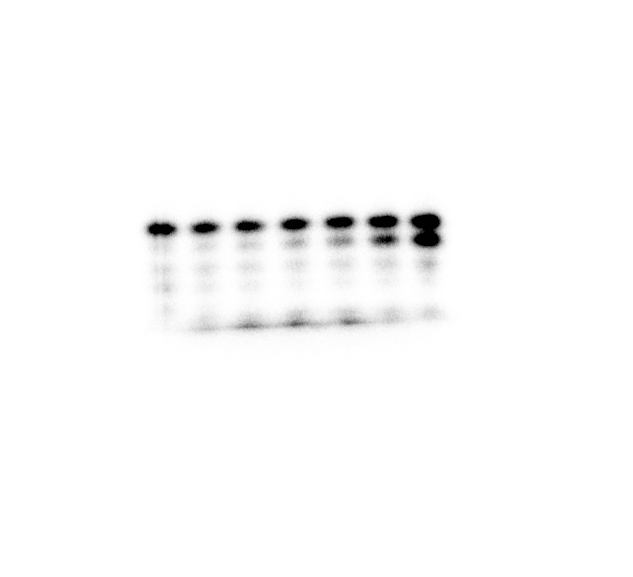

Supplement: Figure 5—source data 1. — Original, unedited images and labeled composite overview of nano-RNase C (NrnC) activity against substrates with different length in three replicates. [file elife-70146-fig5-data1.zip › Figure5_source_data_1/Figure 5A-source data 1 (pAAGGGG).tif]

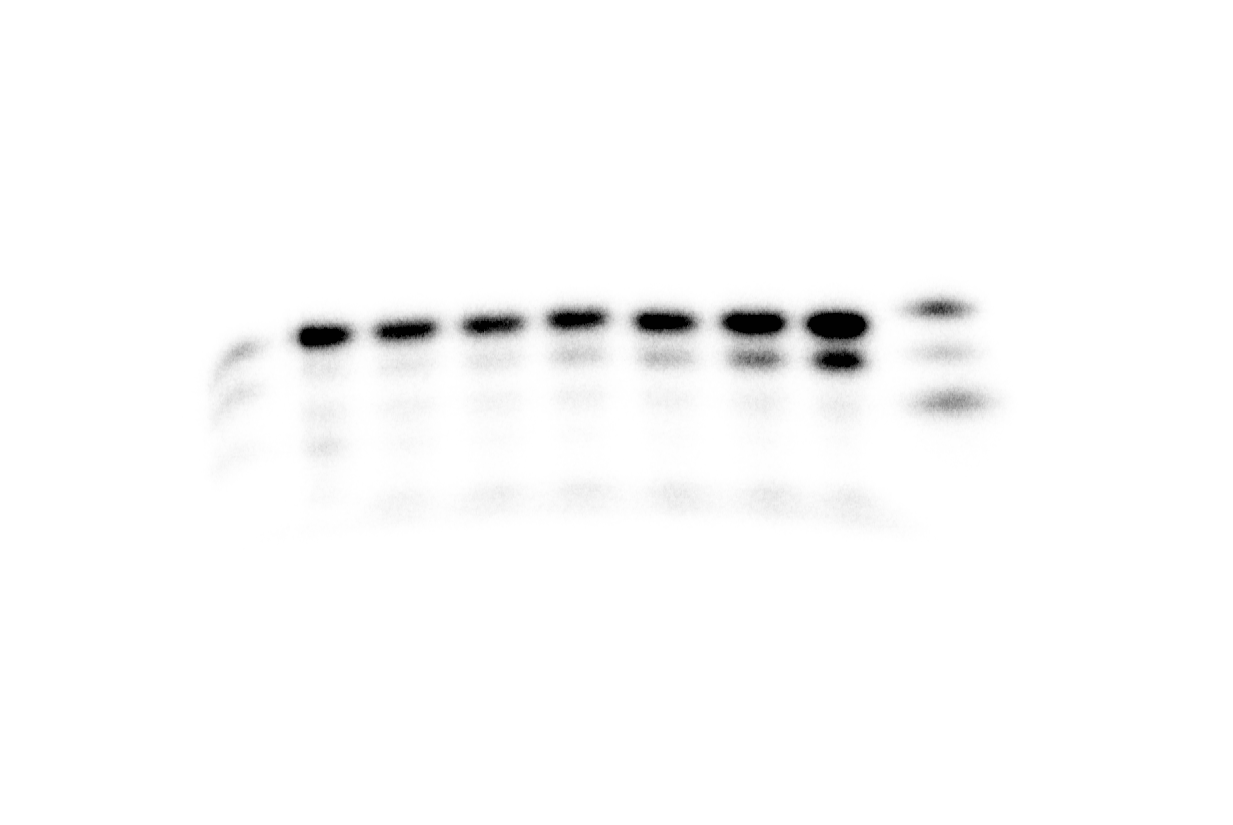

Supplement: Figure 5—source data 1. — Original, unedited images and labeled composite overview of nano-RNase C (NrnC) activity against substrates with different length in three replicates. [file elife-70146-fig5-data1.zip › Figure5_source_data_1/Figure 5A-source data 2 (pAAGGGG).tif]

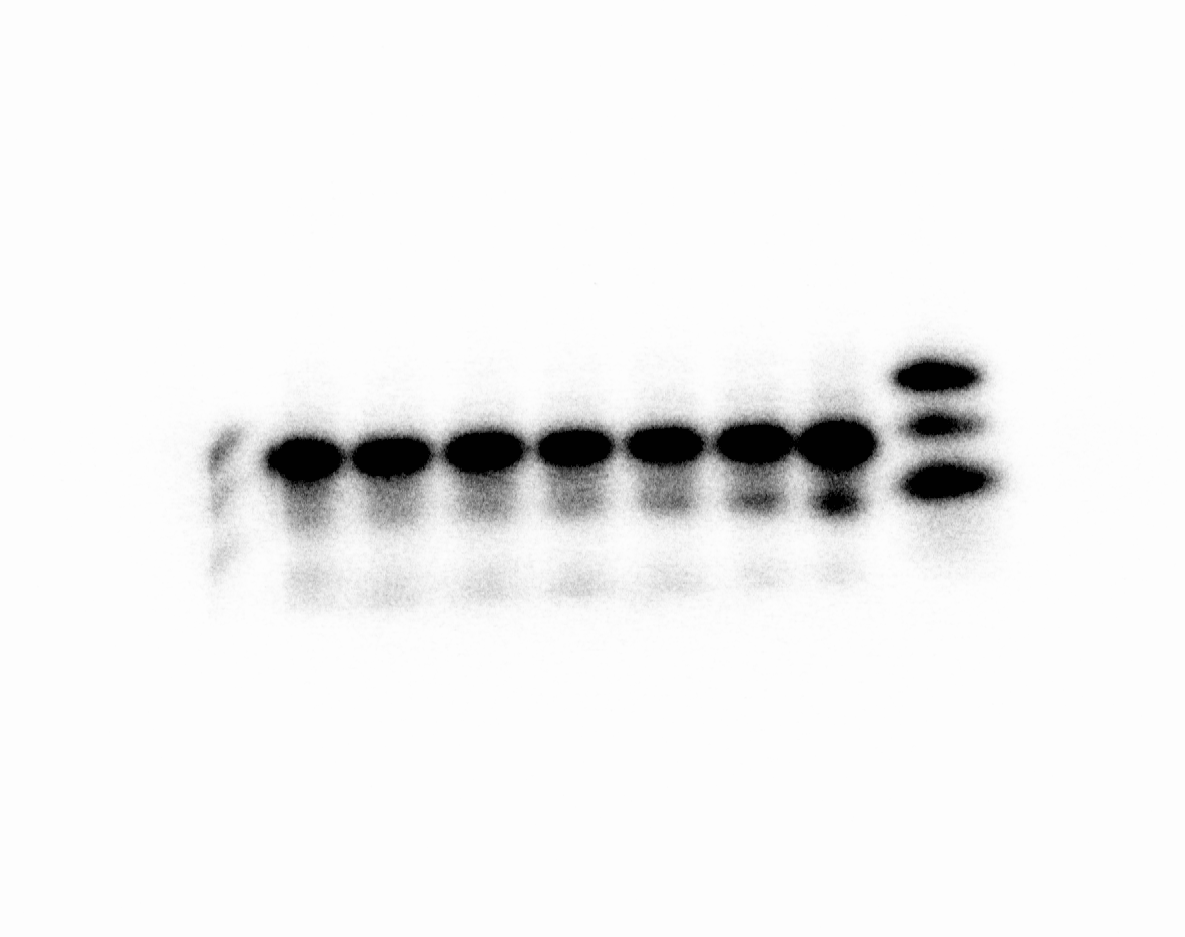

Supplement: Figure 5—source data 1. — Original, unedited images and labeled composite overview of nano-RNase C (NrnC) activity against substrates with different length in three replicates. [file elife-70146-fig5-data1.zip › Figure5_source_data_1/Figure 5A-source data 3 (pAAGG).tif]

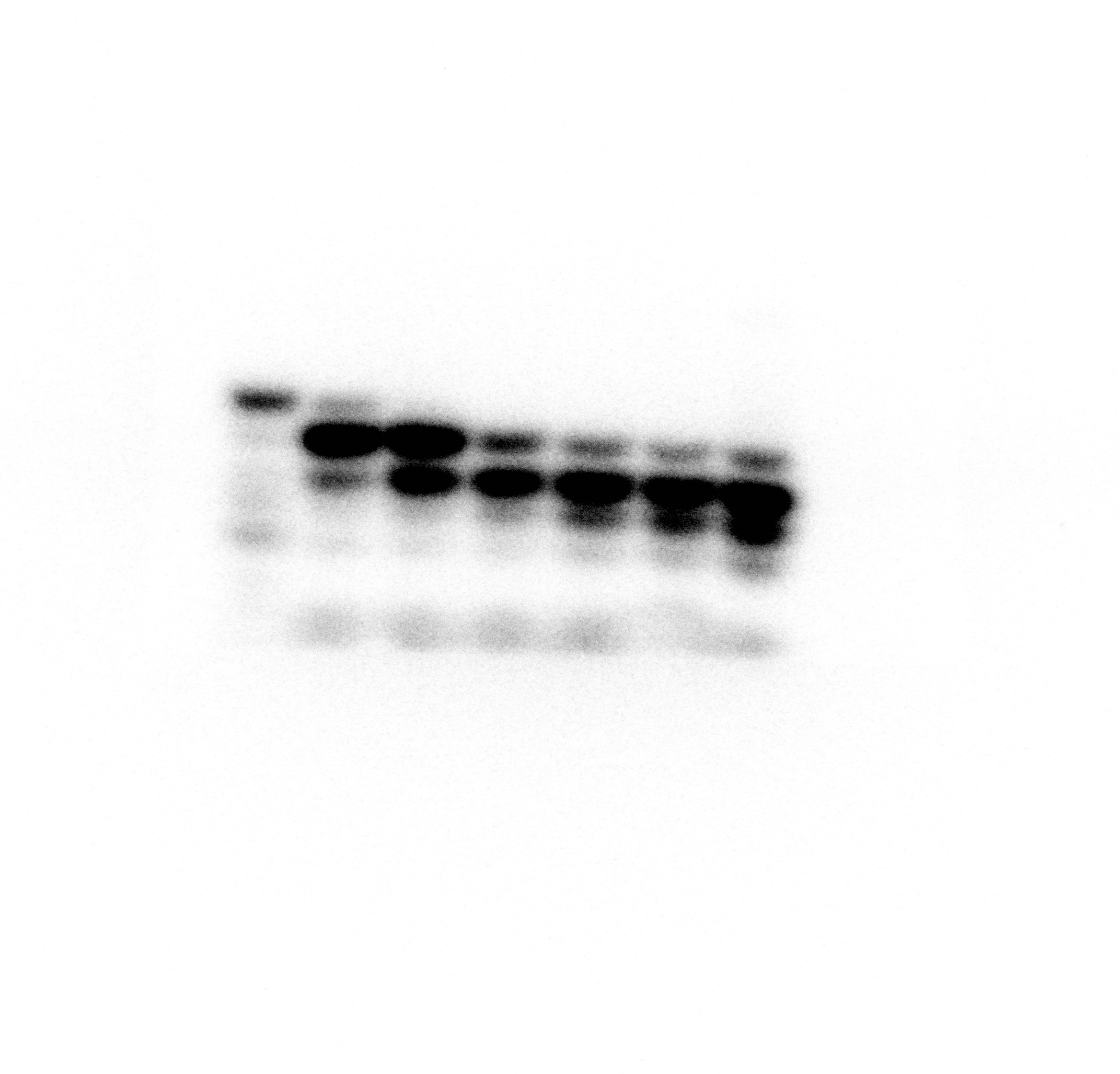

Supplement: Figure 5—source data 2. — Original, unedited images and labeled composite overview of nano-RNase C (NrnC) activity against a 7-nucleotide RNA substrate at increasing enzyme concentration in three replicates. [file elife-70146-fig5-data2.zip › Figure5_source_data_2/Figure 5B-source data 2 (50 nM).tif]

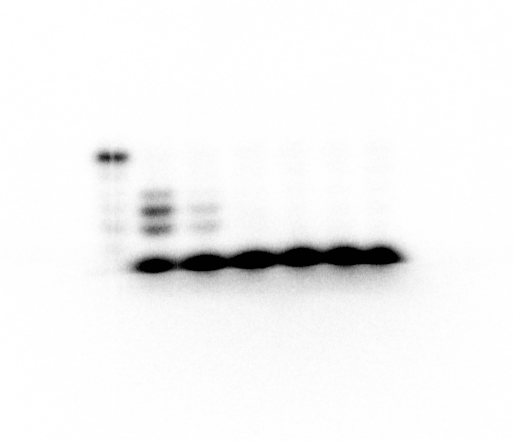

Supplement: Figure 5—source data 2. — Original, unedited images and labeled composite overview of nano-RNase C (NrnC) activity against a 7-nucleotide RNA substrate at increasing enzyme concentration in three replicates. [file elife-70146-fig5-data2.zip › Figure5_source_data_2/Figure 5B-source data 3 (1000 nM).tif]

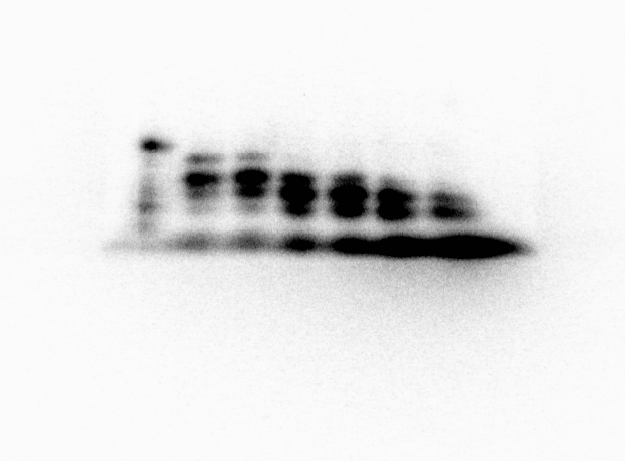

Supplement: Figure 5—source data 2. — Original, unedited images and labeled composite overview of nano-RNase C (NrnC) activity against a 7-nucleotide RNA substrate at increasing enzyme concentration in three replicates. [file elife-70146-fig5-data2.zip › Figure5_source_data_2/Figure 5B-source data 1 (100 nM).tif]

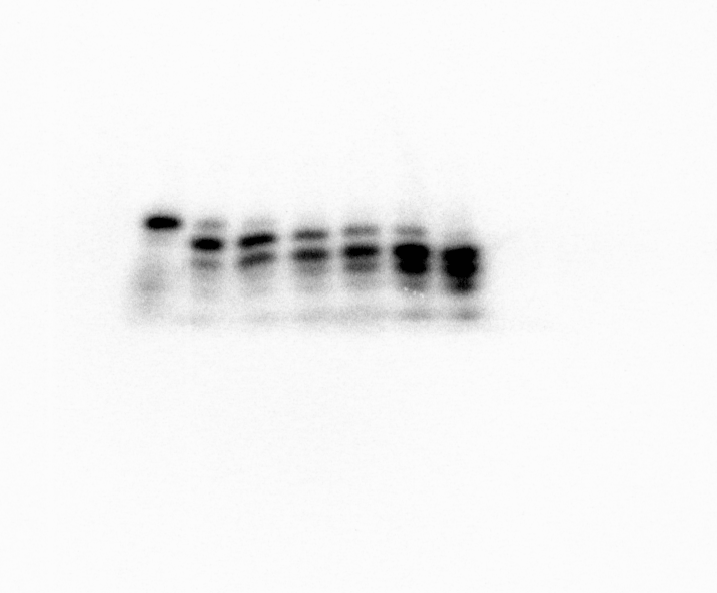

Supplement: Figure 5—source data 2. — Original, unedited images and labeled composite overview of nano-RNase C (NrnC) activity against a 7-nucleotide RNA substrate at increasing enzyme concentration in three replicates. [file elife-70146-fig5-data2.zip › Figure5_source_data_2/Figure 5B-source data 1 (50 nM).tif]

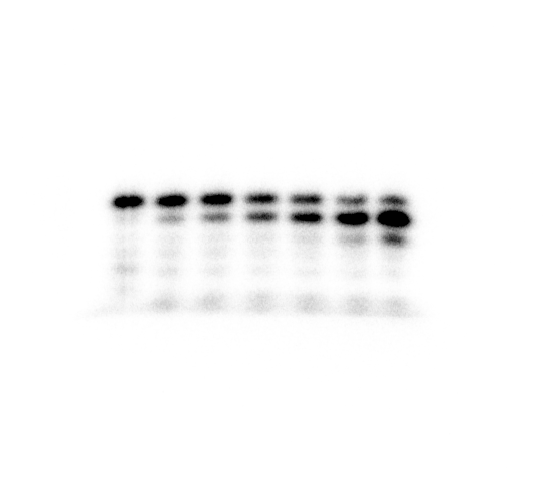

Supplement: Figure 5—source data 2. — Original, unedited images and labeled composite overview of nano-RNase C (NrnC) activity against a 7-nucleotide RNA substrate at increasing enzyme concentration in three replicates. [file elife-70146-fig5-data2.zip › Figure5_source_data_2/Figure 5B-source data 2 (5 nM).tif]

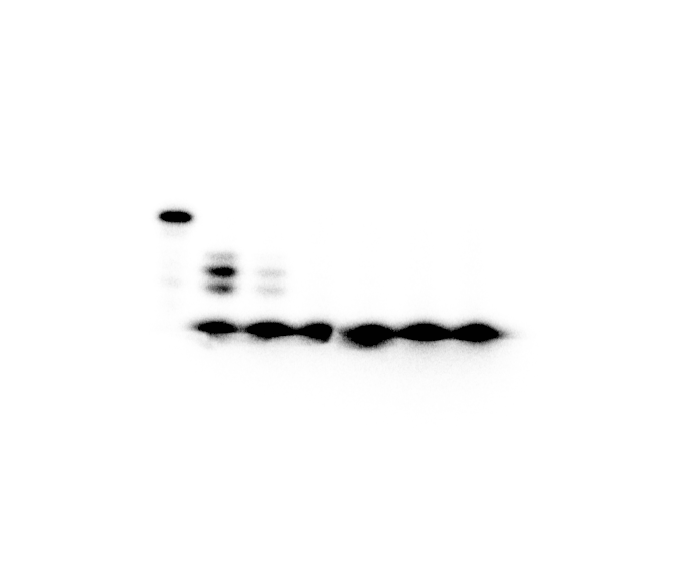

Supplement: Figure 5—source data 2. — Original, unedited images and labeled composite overview of nano-RNase C (NrnC) activity against a 7-nucleotide RNA substrate at increasing enzyme concentration in three replicates. [file elife-70146-fig5-data2.zip › Figure5_source_data_2/Figure 5B-source data 1 (1000 nM).tif]

Replicate 1

Replicate 2

Replicate 3

NrnC 5 nM

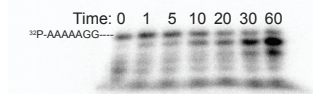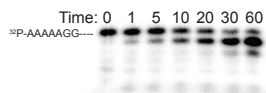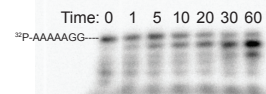

NrnC 50 nM

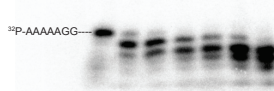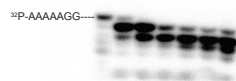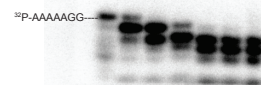

NrnC 100 nM

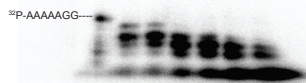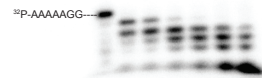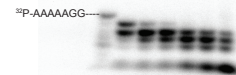

NrnC 1000 nM

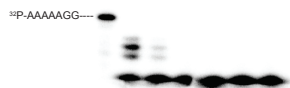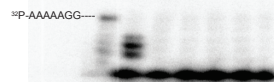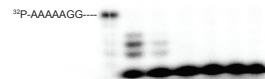

Supplement: Figure 5—source data 2. — Original, unedited images and labeled composite overview of nano-RNase C (NrnC) activity against a 7-nucleotide RNA substrate at increasing enzyme concentration in three replicates. [file elife-70146-fig5-data2.zip › Figure5_source_data_2/Figure 5B Triplicate.pdf]

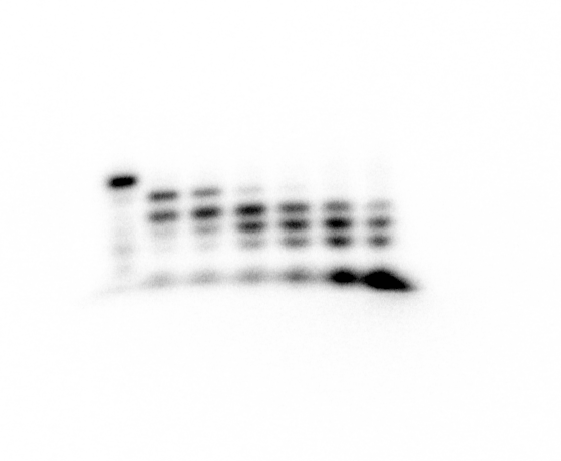

Supplement: Figure 5—source data 2. — Original, unedited images and labeled composite overview of nano-RNase C (NrnC) activity against a 7-nucleotide RNA substrate at increasing enzyme concentration in three replicates. [file elife-70146-fig5-data2.zip › Figure5_source_data_2/Figure 5B-source data 2 (100 nM).tif]

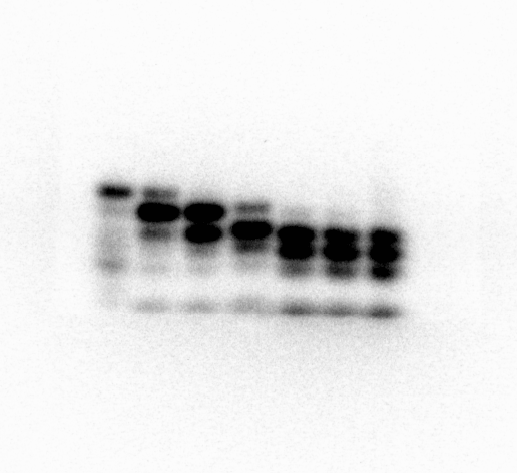

Supplement: Figure 5—source data 2. — Original, unedited images and labeled composite overview of nano-RNase C (NrnC) activity against a 7-nucleotide RNA substrate at increasing enzyme concentration in three replicates. [file elife-70146-fig5-data2.zip › Figure5_source_data_2/Figure 5B-source data 3 (50 nM).tif]

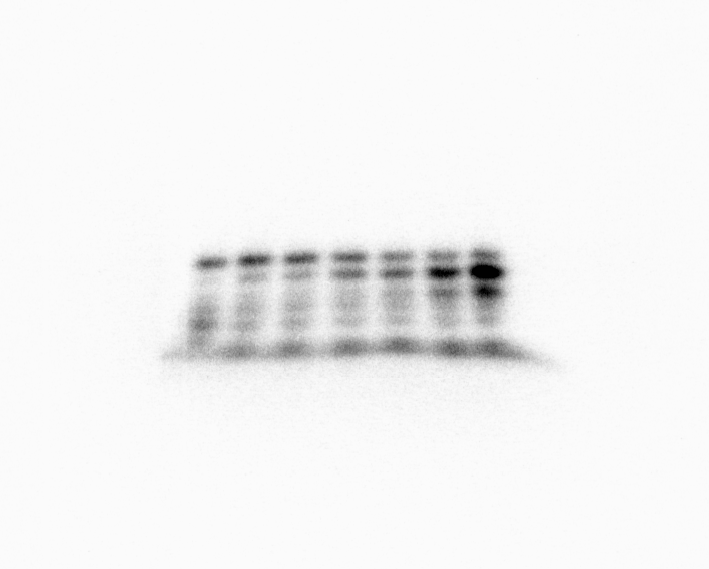

Supplement: Figure 5—source data 2. — Original, unedited images and labeled composite overview of nano-RNase C (NrnC) activity against a 7-nucleotide RNA substrate at increasing enzyme concentration in three replicates. [file elife-70146-fig5-data2.zip › Figure5_source_data_2/Figure 5B-source data 1 (5 nM).tif]

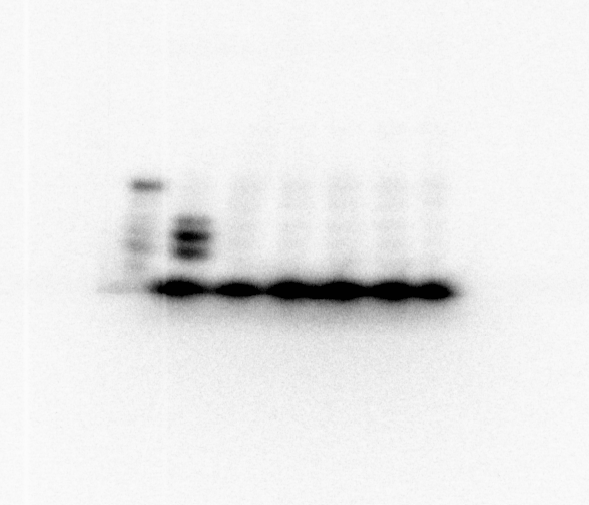

Supplement: Figure 5—source data 2. — Original, unedited images and labeled composite overview of nano-RNase C (NrnC) activity against a 7-nucleotide RNA substrate at increasing enzyme concentration in three replicates. [file elife-70146-fig5-data2.zip › Figure5_source_data_2/Figure 5B-source data 2 (1000 nM).tif]

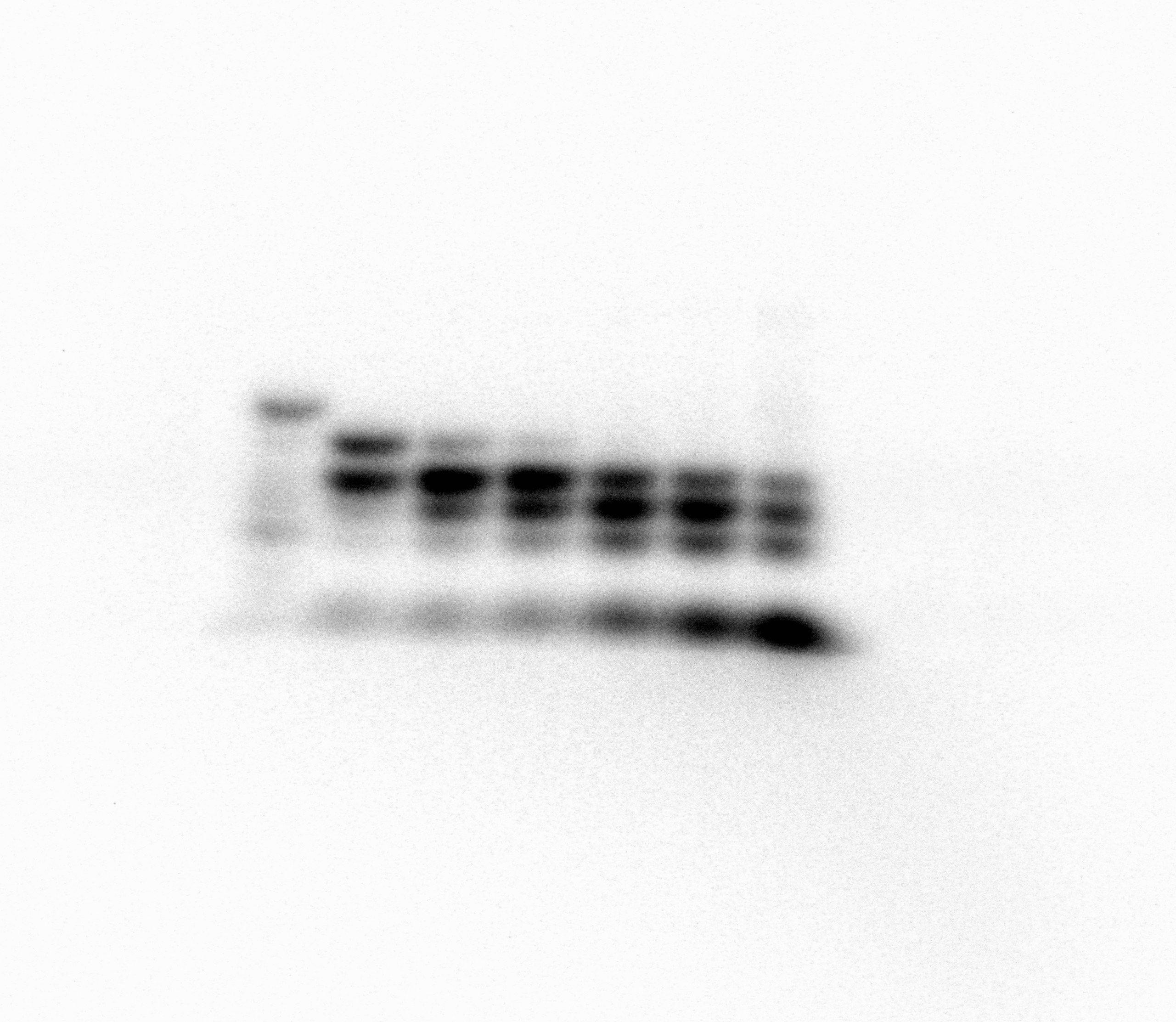

Supplement: Figure 5—source data 2. — Original, unedited images and labeled composite overview of nano-RNase C (NrnC) activity against a 7-nucleotide RNA substrate at increasing enzyme concentration in three replicates. [file elife-70146-fig5-data2.zip › Figure5_source_data_2/Figure 5B-source data 3 (100 nM).tif]

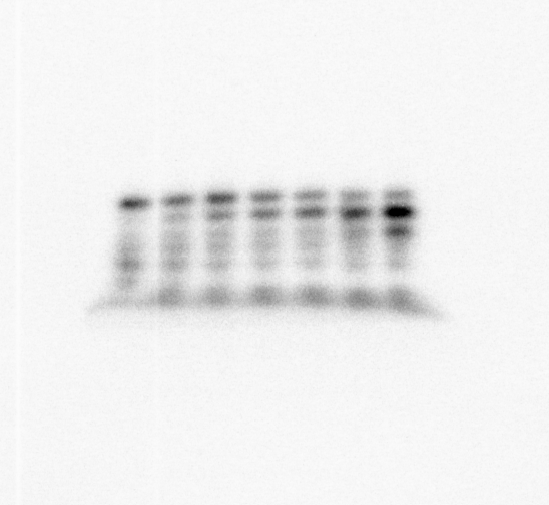

Supplement: Figure 5—source data 2. — Original, unedited images and labeled composite overview of nano-RNase C (NrnC) activity against a 7-nucleotide RNA substrate at increasing enzyme concentration in three replicates. [file elife-70146-fig5-data2.zip › Figure5_source_data_2/Figure 5B-source data 3 (5 nM).tif]

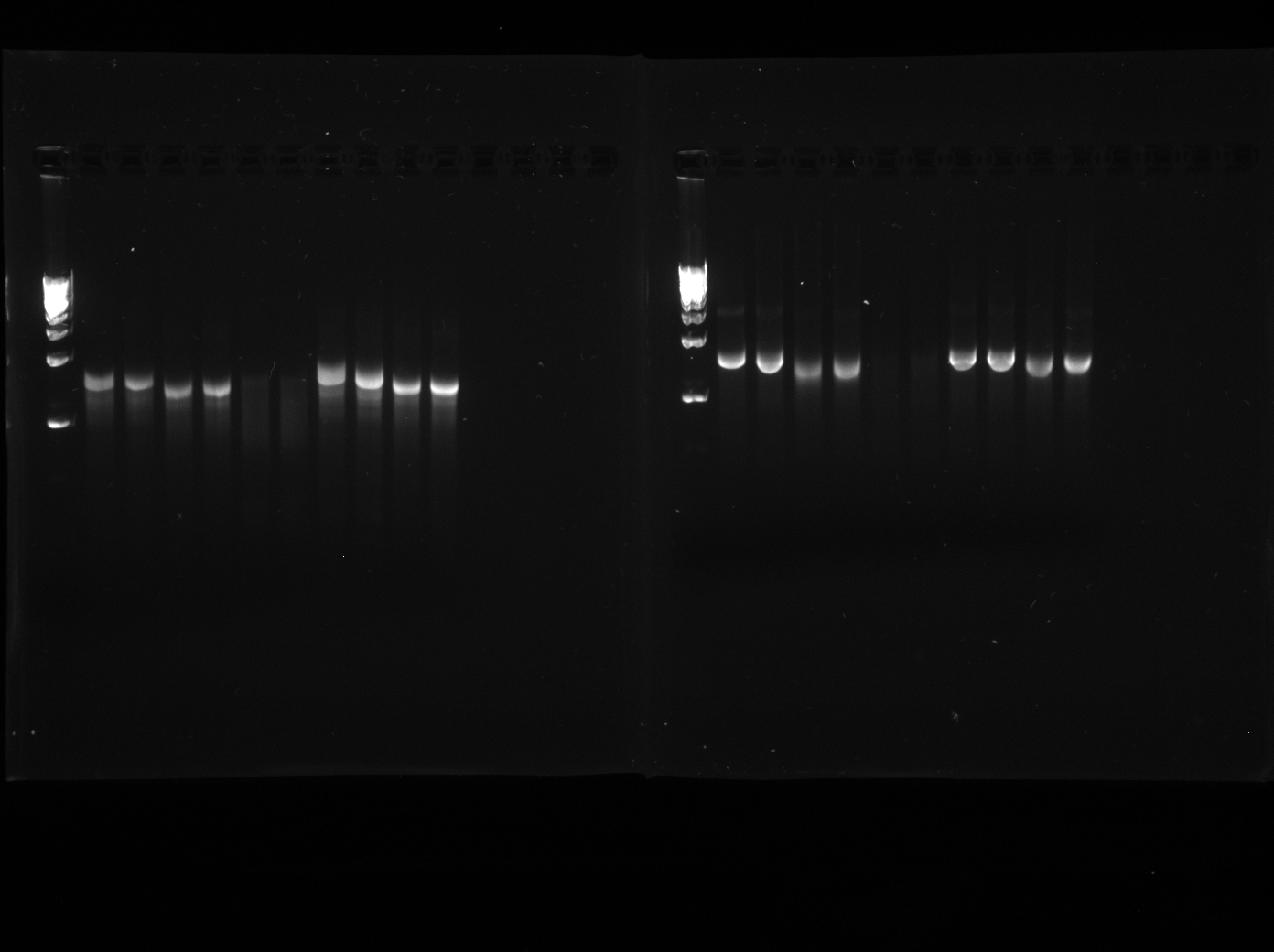

Supplement: Figure 5—figure supplement 2—source data 1. [file elife-70146-fig5-figsupp2-data1.zip › Figure5_figure_supplement2_source_data_1/Figure5_figuresupplement2_source_data_NotI_KpnI.tif]

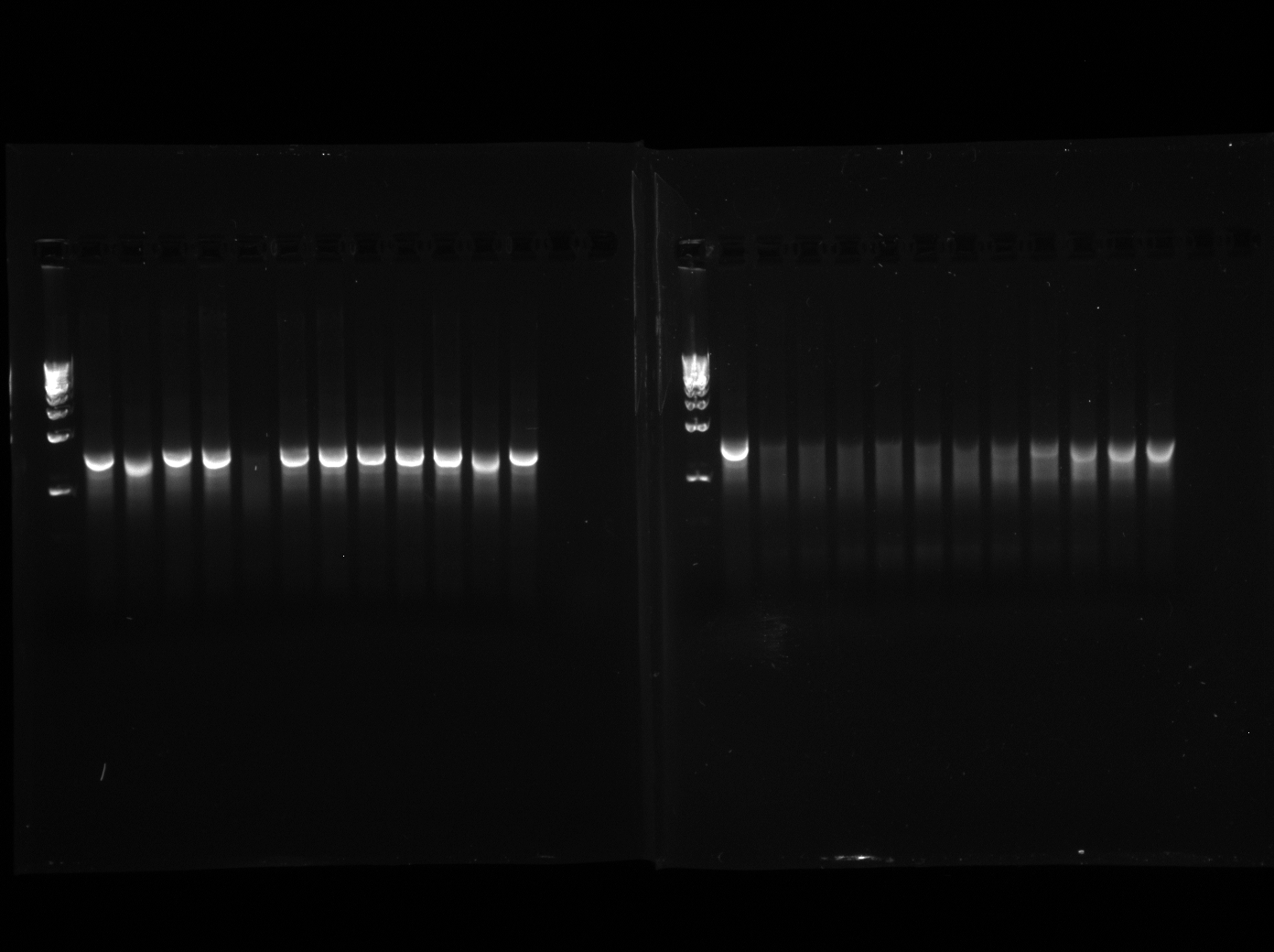

Supplement: Figure 5—figure supplement 2—source data 1. [file elife-70146-fig5-figsupp2-data1.zip › Figure5_figure_supplement2_source_data_1/Figure5_figuresupplement2_source_data_cations_salt.tif]

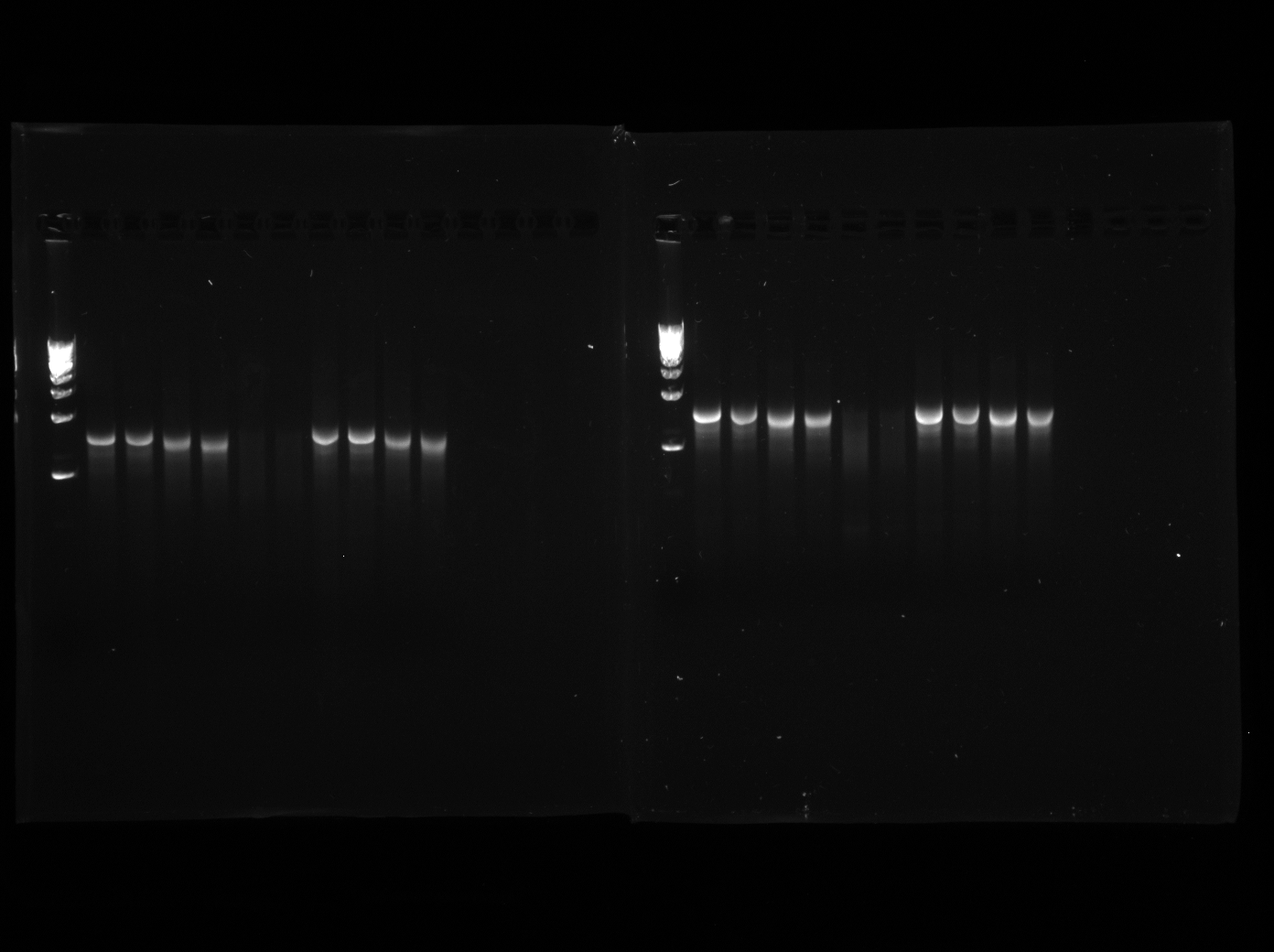

Supplement: Figure 5—figure supplement 2—source data 1. [file elife-70146-fig5-figsupp2-data1.zip › Figure5_figure_supplement2_source_data_1/Figure5_figuresupplement2_source_data_blunt_NdeI.tif]

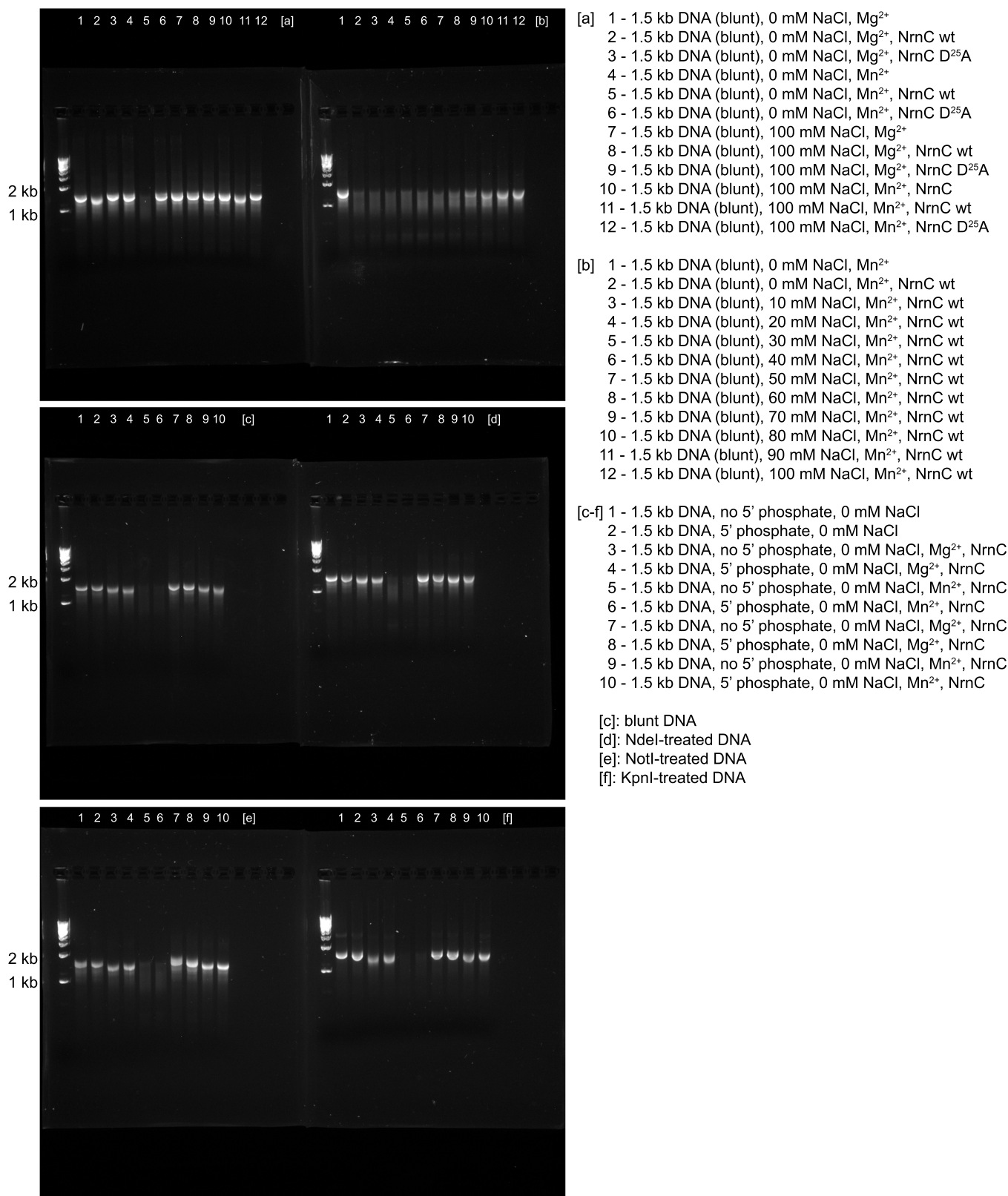

Figure 5 - figure supplement 2 - source data 4

Supplement: Figure 5—figure supplement 2—source data 1. [file elife-70146-fig5-figsupp2-data1.zip › Figure5_figure_supplement2_source_data_1/Figure5_figure_supplment2_source_data_composite.pdf]

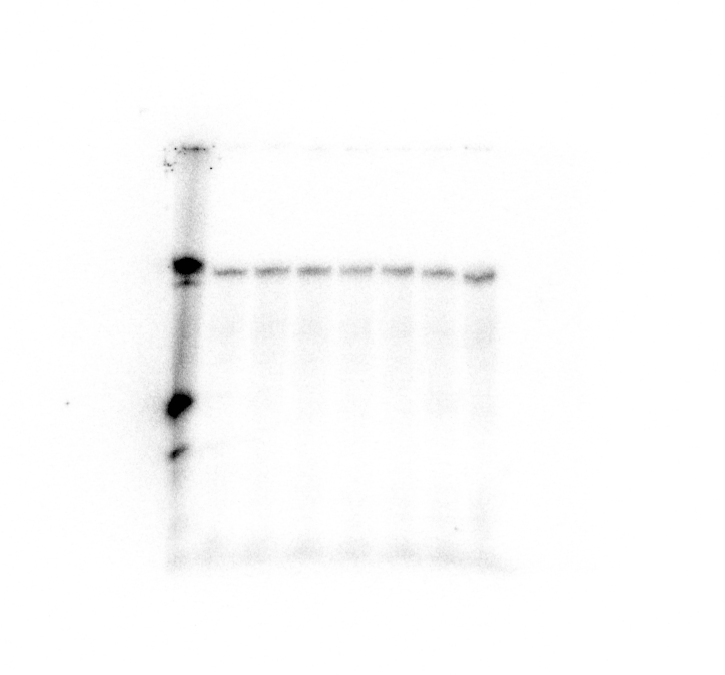

Supplement: Figure 5—figure supplement 3—source data 1. — Original, unedited images and labeled composite overview of nano-RNase C (NrnC) activity against double-stranded DNA oligonucleotides. [file elife-70146-fig5-figsupp3-data1.zip › Figure5_figure_supplement3_source_data_1/Figure 5-figure supplement 3B-source data 3 (3'overhang).tif]

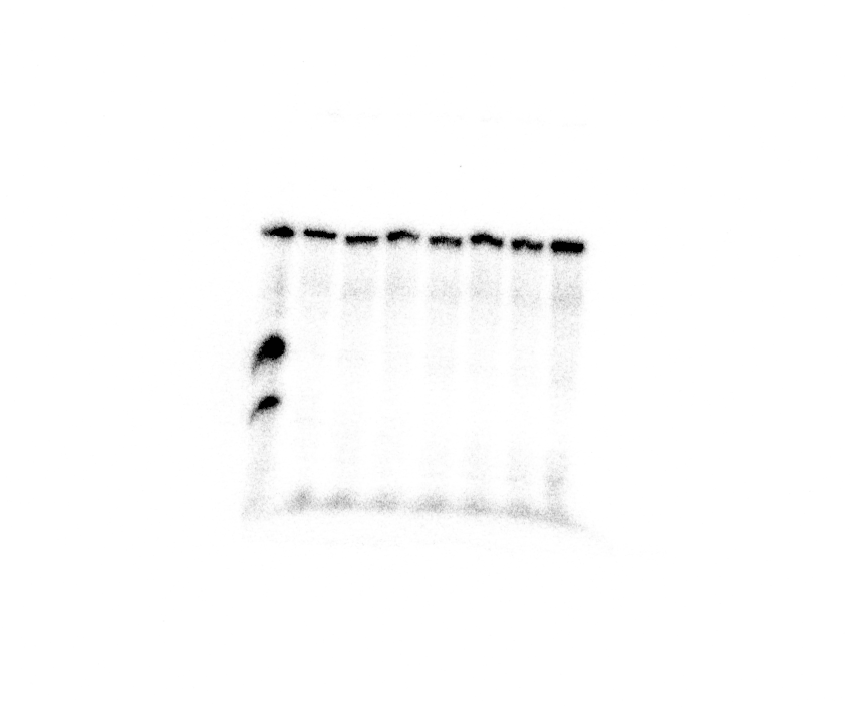

Supplement: Figure 5—figure supplement 3—source data 1. — Original, unedited images and labeled composite overview of nano-RNase C (NrnC) activity against double-stranded DNA oligonucleotides. [file elife-70146-fig5-figsupp3-data1.zip › Figure5_figure_supplement3_source_data_1/Figure 5-figure supplement 3B-source data 2 (3'overhang).tif]

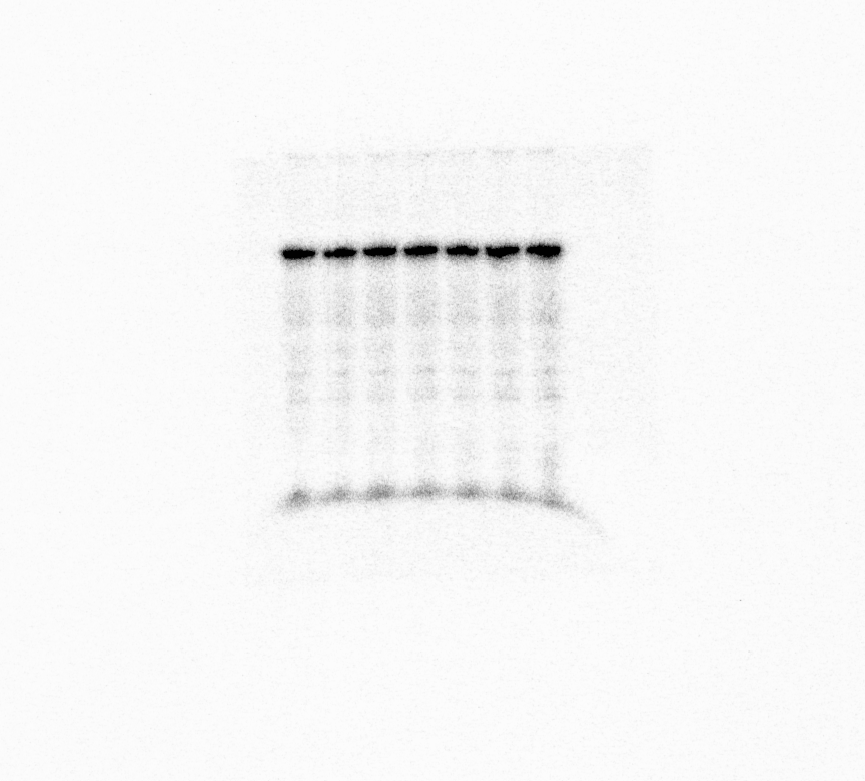

Supplement: Figure 5—figure supplement 3—source data 1. — Original, unedited images and labeled composite overview of nano-RNase C (NrnC) activity against double-stranded DNA oligonucleotides. [file elife-70146-fig5-figsupp3-data1.zip › Figure5_figure_supplement3_source_data_1/Figure 5-figure supplement 3B-source data 1 (5'overhang).tif]

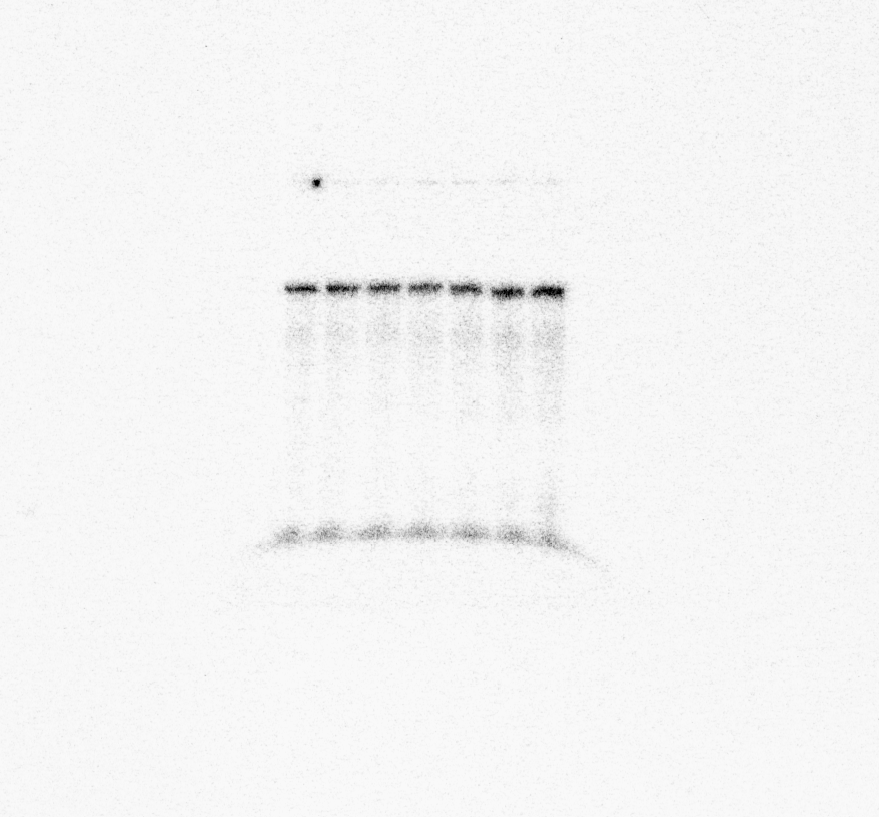

Supplement: Figure 5—figure supplement 3—source data 1. — Original, unedited images and labeled composite overview of nano-RNase C (NrnC) activity against double-stranded DNA oligonucleotides. [file elife-70146-fig5-figsupp3-data1.zip › Figure5_figure_supplement3_source_data_1/Figure 5-figure supplement 3B-source data 1 (3'overhang).tif]

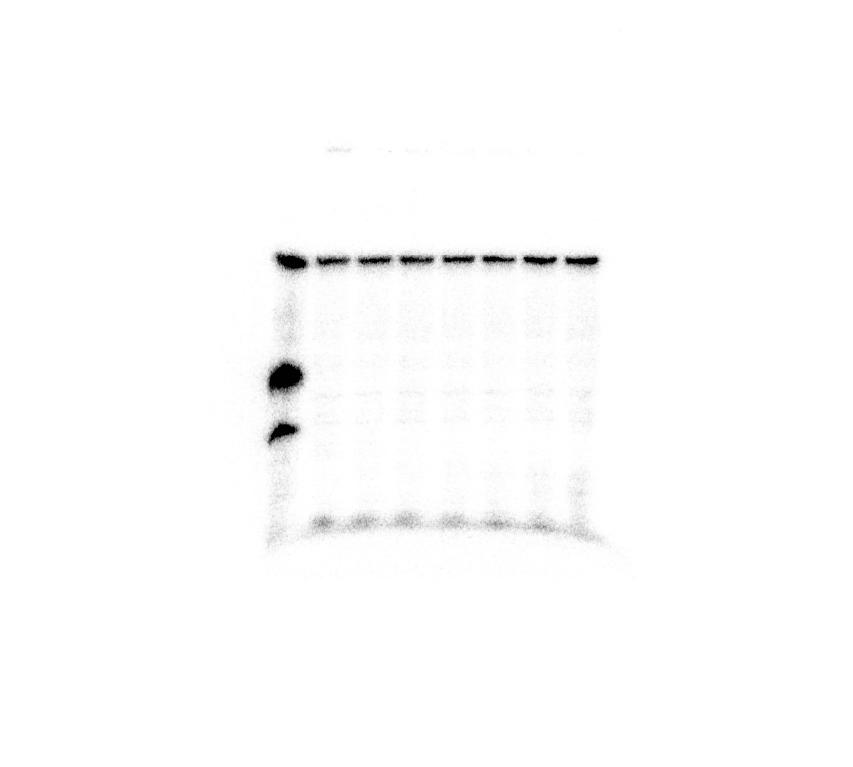

Supplement: Figure 5—figure supplement 3—source data 1. — Original, unedited images and labeled composite overview of nano-RNase C (NrnC) activity against double-stranded DNA oligonucleotides. [file elife-70146-fig5-figsupp3-data1.zip › Figure5_figure_supplement3_source_data_1/Figure 5-figure supplement 3B-source data 2 (5'overhang).tif]

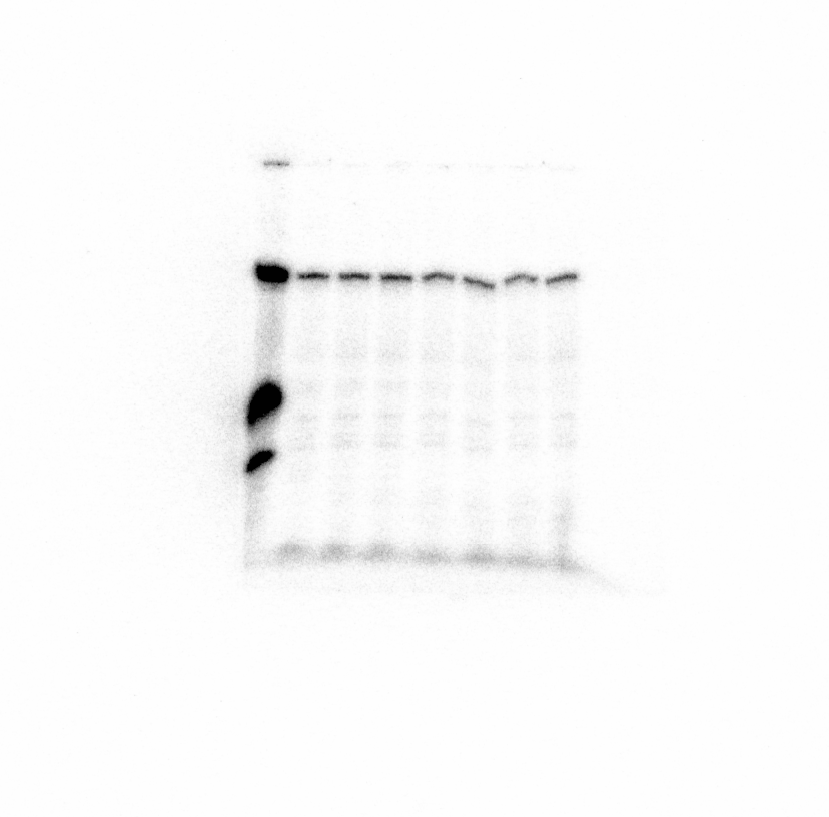

Supplement: Figure 5—figure supplement 3—source data 1. — Original, unedited images and labeled composite overview of nano-RNase C (NrnC) activity against double-stranded DNA oligonucleotides. [file elife-70146-fig5-figsupp3-data1.zip › Figure5_figure_supplement3_source_data_1/Figure 5-figure supplement 3B-source data 3 (5'overhang).tif]

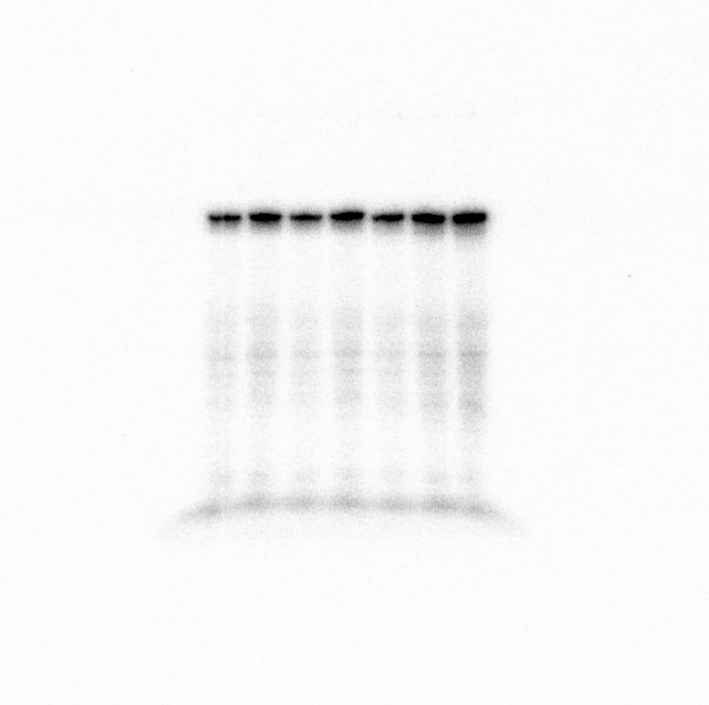

Supplement: Figure 5—figure supplement 3—source data 2. — Original, unedited images and labeled composite overview of nano-RNase C (NrnC) activity against double-stranded RNA oligonucleotides. [file elife-70146-fig5-figsupp3-data2.zip › Figure5_figure_supplement3_source_data_2/Figure 5-figure supplement 3C-source data 2 (3'overhang).tif]

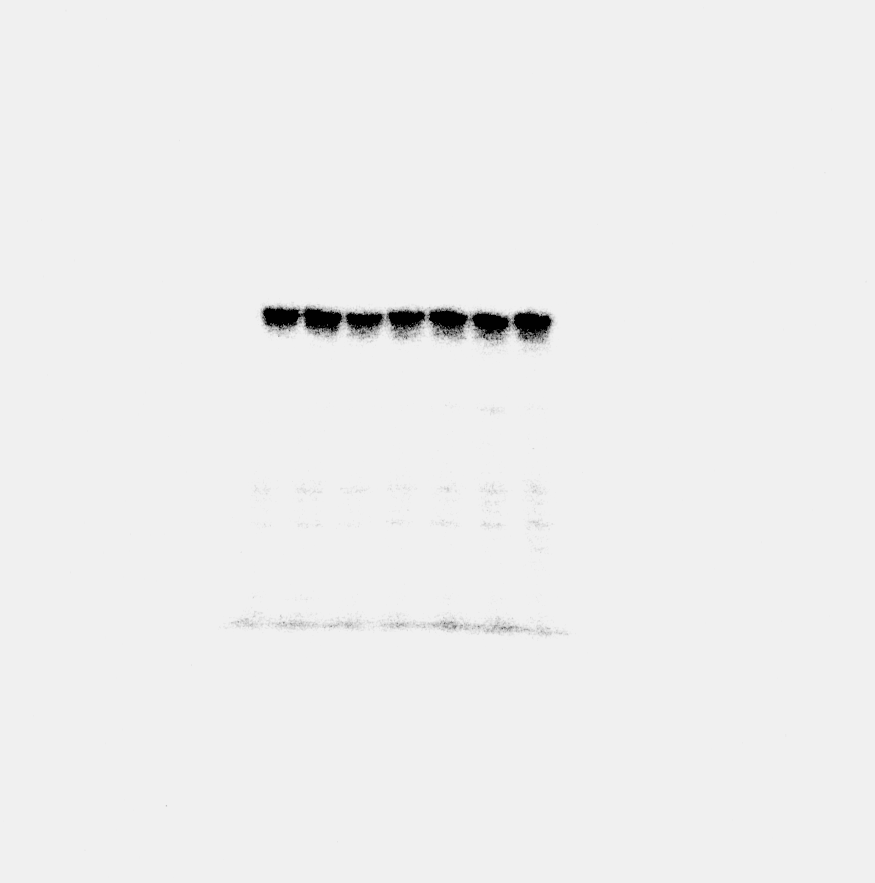

Supplement: Figure 5—figure supplement 3—source data 2. — Original, unedited images and labeled composite overview of nano-RNase C (NrnC) activity against double-stranded RNA oligonucleotides. [file elife-70146-fig5-figsupp3-data2.zip › Figure5_figure_supplement3_source_data_2/Figure 5-figure supplement 3C-source data 1 (5'overhang).tif]

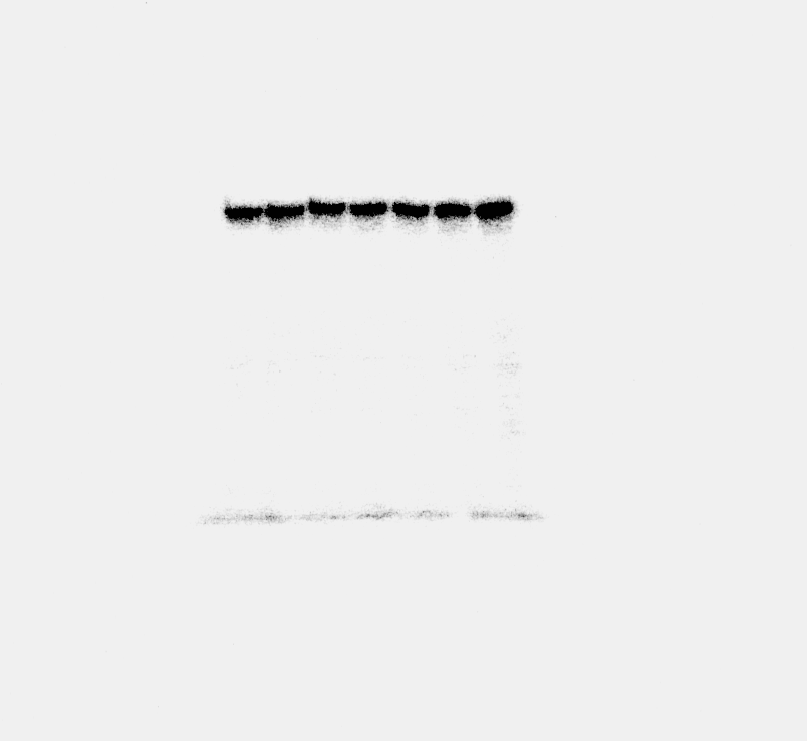

Supplement: Figure 5—figure supplement 3—source data 2. — Original, unedited images and labeled composite overview of nano-RNase C (NrnC) activity against double-stranded RNA oligonucleotides. [file elife-70146-fig5-figsupp3-data2.zip › Figure5_figure_supplement3_source_data_2/Figure 5-figure supplement 3C-source data 3 (3'overhang).tif]

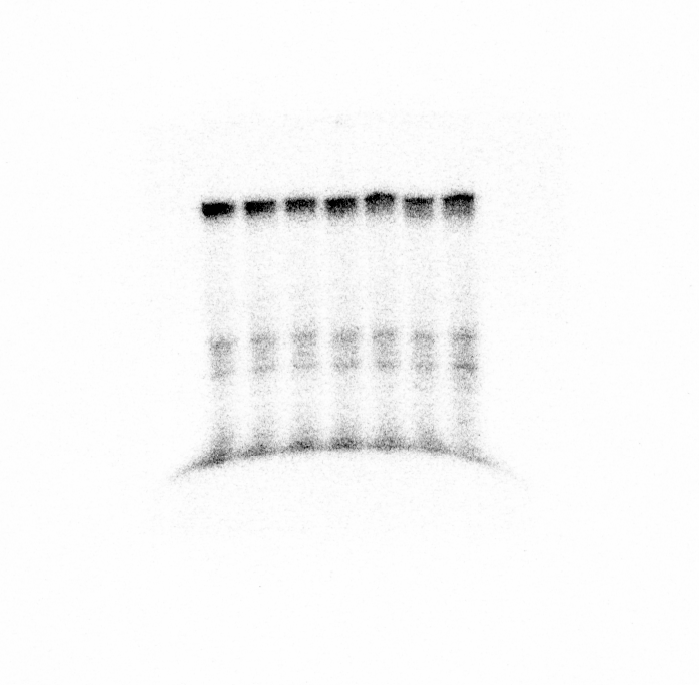

Supplement: Figure 5—figure supplement 3—source data 2. — Original, unedited images and labeled composite overview of nano-RNase C (NrnC) activity against double-stranded RNA oligonucleotides. [file elife-70146-fig5-figsupp3-data2.zip › Figure5_figure_supplement3_source_data_2/Figure 5-figure supplement 3C-source data 3 (5'overhang).tif]

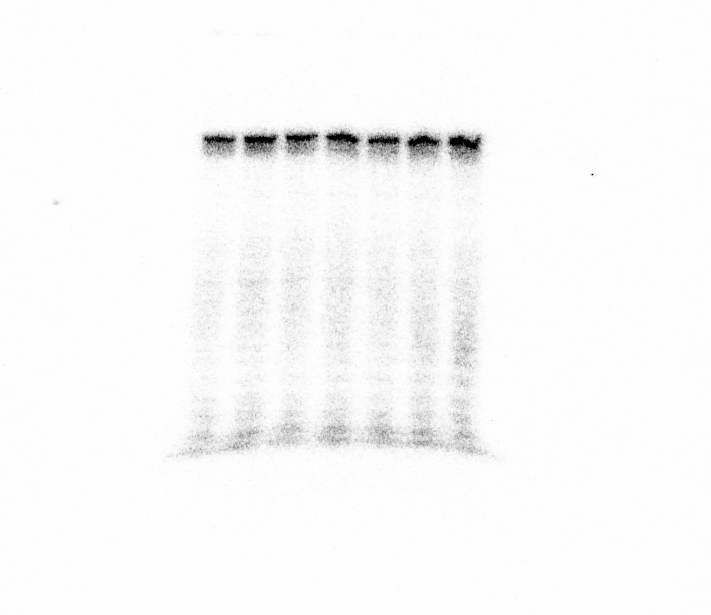

Supplement: Figure 5—figure supplement 3—source data 2. — Original, unedited images and labeled composite overview of nano-RNase C (NrnC) activity against double-stranded RNA oligonucleotides. [file elife-70146-fig5-figsupp3-data2.zip › Figure5_figure_supplement3_source_data_2/Figure 5-figure supplement 3C-source data 1 (3'overhang).tif]

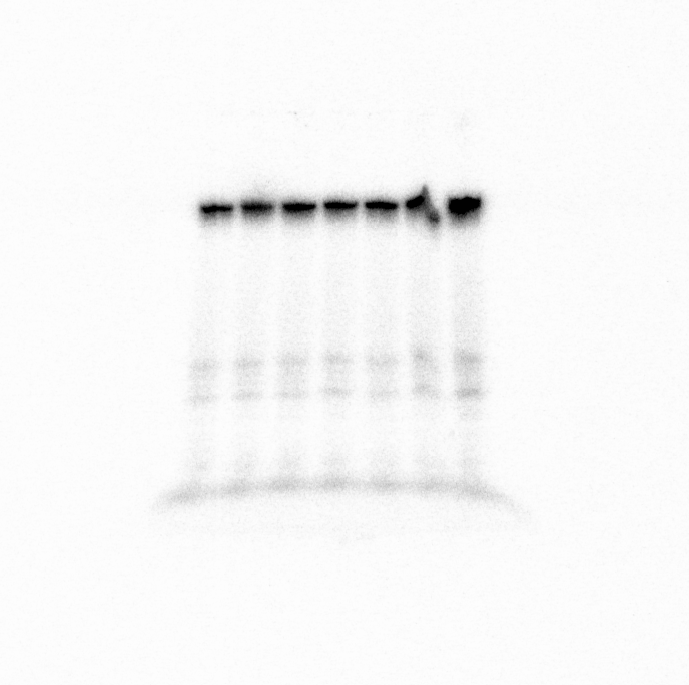

Supplement: Figure 5—figure supplement 3—source data 2. — Original, unedited images and labeled composite overview of nano-RNase C (NrnC) activity against double-stranded RNA oligonucleotides. [file elife-70146-fig5-figsupp3-data2.zip › Figure5_figure_supplement3_source_data_2/Figure 5-figure supplement 3C-source data 2 (5'overhang).tif]
