# Supplementary material for: Structural characterization of NrnC identifies unifying features of dinucleases
Source: eLife. 2021 Sep 17;10:e70146. doi: 10.7554/eLife.70146 (PMC8492067; doi:10.7554/eLife.70146)
Supplement: Figure 2—source data 4. — Original, unedited images and labeled composite image of P. aeruginosa colony growth. [file elife-70146-fig2-data4.zip › Figure2_source_data_4/Figure2D_source_data_3.pdf]

1 2 3 4 5 6 7 8

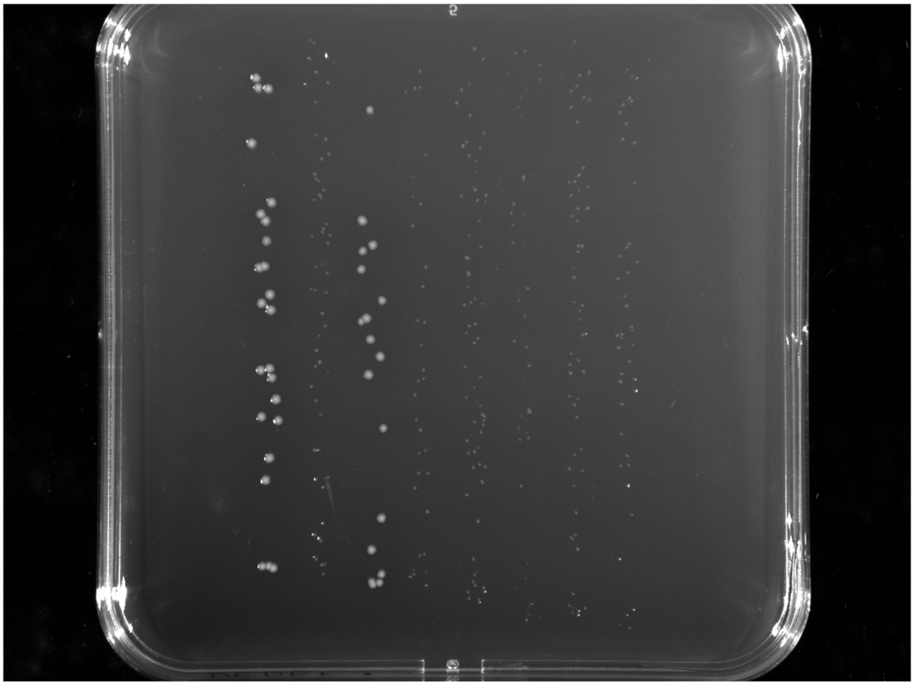

1 - *P. aeruginosa* PA14
2 - PA14 Δ*orn*
3 - PA14 Δ*orn* + pNrnC wt
4 - PA14 Δ*orn* + pNrnC D^{25}A
5 - PA14 Δ*orn* + pNrnC E^{27}A
6 - PA14 Δ*orn* + pNrnC D^{84}A
7 - PA14 Δ*orn* + pNrnC D^{155}A
8 - PA14 Δ*orn* + pNrnC Y^{151}A

9 10 11 12 13 14 15 16

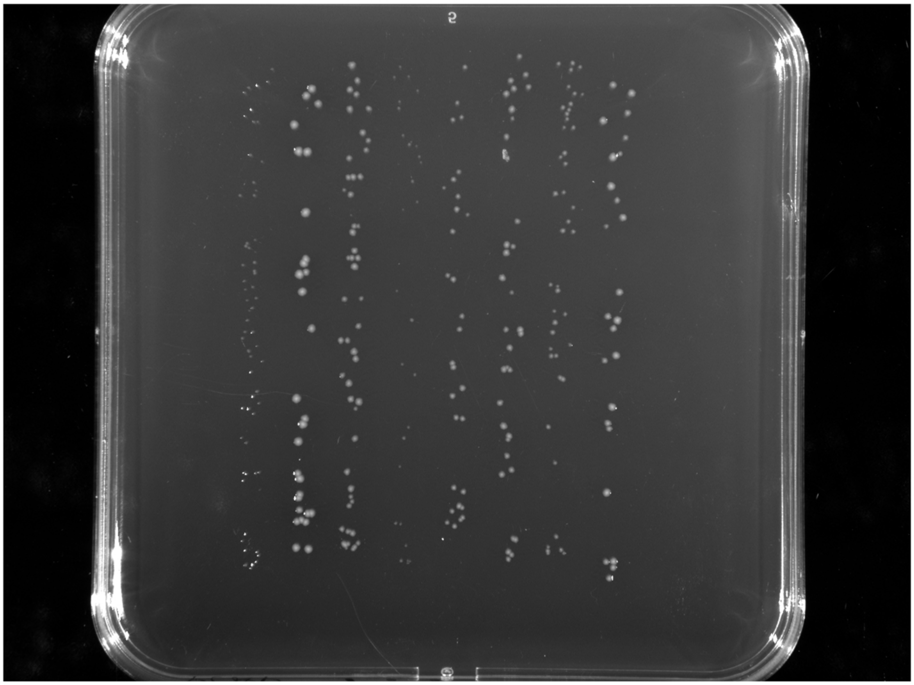

9 - PA14 Δ*orn*
10 - PA14 Δ*orn* + pNrnC wt
11 - PA14 Δ*orn* + pNrnC L^{31}A
12 - PA14 Δ*orn* + pNrnC H^{79}A
13 - PA14 Δ*orn* + pNrnC K^{103}A
14 - PA14 Δ*orn* + pNrnC H^{205}A
15 - PA14 Δ*orn* + pNrnC K^{132}A
16 - PA14 Δ*orn* + pNrnC Q^{135}A

Figure 2D - source data 1
