# Supplementary material for: Structural characterization of NrnC identifies unifying features of dinucleases
Source: eLife. 2021 Sep 17;10:e70146. doi: 10.7554/eLife.70146 (PMC8492067; doi:10.7554/eLife.70146)
Supplement: Figure 5—figure supplement 3—source data 1. — Original, unedited images and labeled composite overview of nano-RNase C (NrnC) activity against double-stranded DNA oligonucleotides. [file elife-70146-fig5-figsupp3-data1.zip › Figure5_figure_supplement3_source_data_1/Figure 5_S3B.pdf]

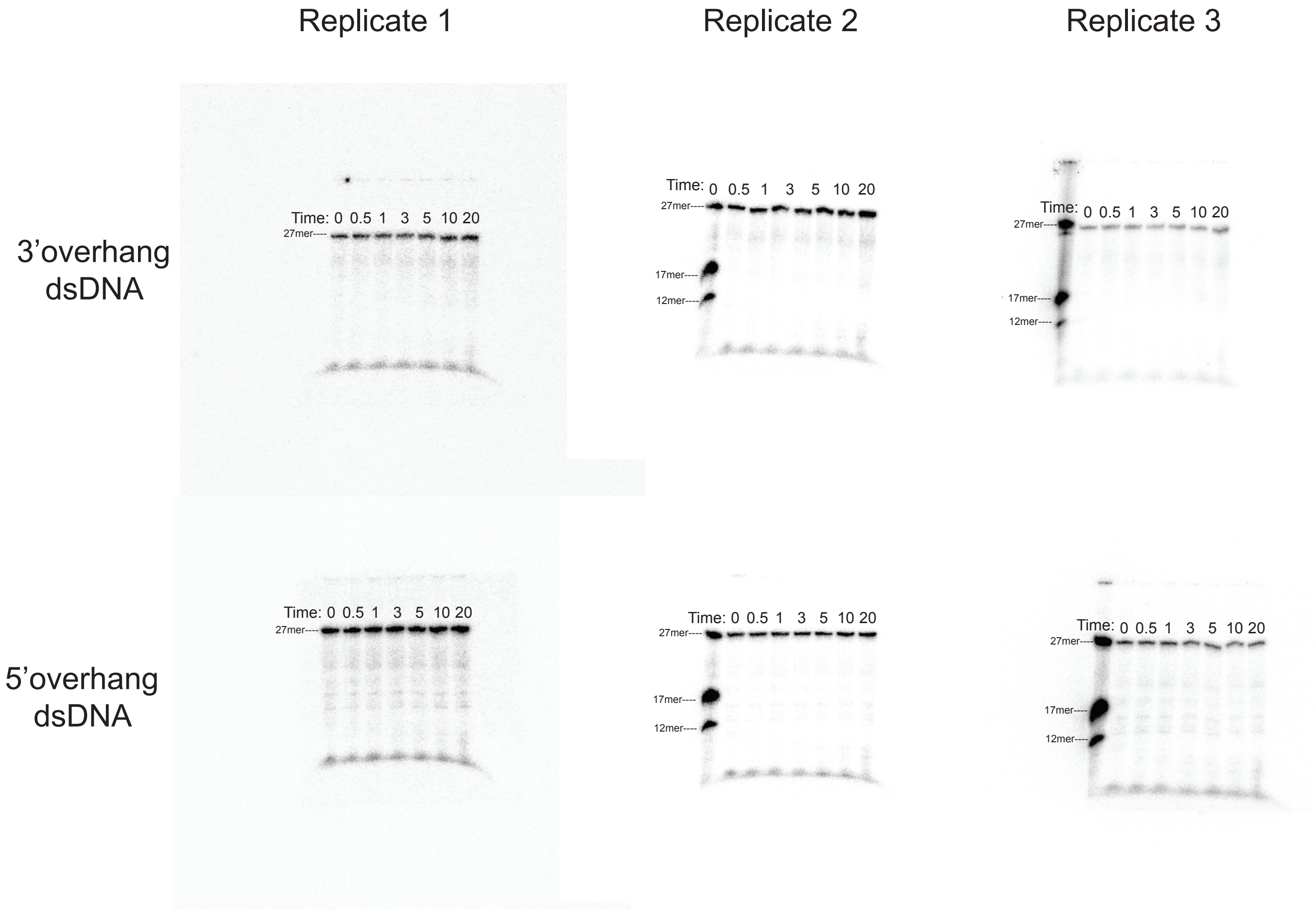
Replicate 1
Replicate 2
Replicate 3
3'overhang dsDNA
5'overhang dsDNA
Time: 0 0.5 1 3 5 10 20
27mer----
Time: 0 0.5 1 3 5 10 20
27mer----
17mer----
12mer----
Time: 0 0.5 1 3 5 10 20
27mer----
17mer----
12mer----
Time: 0 0.5 1 3 5 10 20
27mer----
Time: 0 0.5 1 3 5 10 20
27mer----
17mer----
12mer----
Time: 0 0.5 1 3 5 10 20
27mer----
17mer----
12mer----
