# Supplementary material for: Structural characterization of NrnC identifies unifying features of dinucleases
Source: eLife. 2021 Sep 17;10:e70146. doi: 10.7554/eLife.70146 (PMC8492067; doi:10.7554/eLife.70146)
Supplement: Supplementary file 1. [file elife-70146-supp1.docx]

| **Supplementary File 1. Data collection and refinement statistics.^a^** | | | | | | |
| --- | --- | --- | --- | --- | --- | --- |
| ***Name*** | NrnC•pGpG | NrnC•pApA | NrnC•pGpC | NrnC•pAp | NrnC•pGpG/Mg^2+^ | NrnC•pGpG/Mn^2+^ |
| ***Organism*** | *B. henselae* | *B. henselae* | *B. henselae* | *B. henselae* | *B. henselae* | *B. henselae* |
| ***Ligand*** | pGpG | pApA | pGpC | pAp | 2GMP/Mg2+ | 2GMP/Mn2+ |
| ***Data collection*** | | | | | | |
| X-ray source | APS 24-ID-E | CHESS 7B2 | CHESS 7B2 | CHESS 7B2 | APS 24-ID-C | APS 24-ID-C |
| Wavelength | 0.9792 | 1.000 | 1.000 | 1.000 | 0.9792 | 1.8917 |
| Resolution range (Å) | 64.4-1.80 | 42.7-1.95 | 47.2-1.94 | 47.2-1.95 | 127-2.00 | 128-2.35 |
| Space group | P21 | P21 | P212121 | P212121 | P21 | P21 |
| Unit cell a/b/c (Å) | 71.2 128.3 129.3 | 70.8 127.5 128.6 | 100.1 142.4 148.8 | 99.7 141.7 147.9 | 70.9 126.7 127.1 | 71.7 127.8 128.8 |
| Unit cell α/β/γ (°) | 90 94.7 90 | 90 95.3 90 | 90 90 90 | 90 90 90 | 90 94.3 90 | 90 94.9 90 |
| Total reflections | 889162 (80298) | 615957 (61235) | 1031986 (104247) | 1121813 (108055) | 616220 (52575) | 589748 (79845) |
| Unique reflections | 212143 (20933) | 164518 (16396) | 155943 (15333) | 152553 (15073) | 148330 (14541) | 174471 (12956) |
| Multiplicity | 4.2 (3.8) | 3.7 (3.7) | 6.6 (6.8) | 7.4 (7.2) | 4.2 (3.6) | 6.6 (6.2) |
| Completeness (%) | 99.21 (98.12) | 99.68 (99.53) | 99.71 (99.13) | 99.89 (99.90) | 98.14 (95.95) | 98.8 (98.5) |
| I/sigma(I) | 17.50 (1.43) | 11.98 (2.36) | 9.08 (1.72) | 14.04 (2.46) | 18.57 (1.94) | 11.0 (1.9) |
| Wilson B-factor | 30.72 | 26.37 | 24.26 | 25.11 | 33.48 | 46.67 |
| CC1/2 | 0.999 (0.602) | 0.997 (0.775) | 0.998 (0.778) | 0.999 (0.877) | 0.992 (0.444) | 0.994 (0.709) |
| ***Refinement*** | | | | | | |
| Reflections (R-free) | 10608 (1073) | 1990 (198) | 1999 (195) | 1990 (198) | 2004 (197) | 4387 (449) |
| R-work | 15.09 | 14.51 | 19.41 | 16.43 | 15.80 | 16.19 |
| R-free | 17.11 | 17.96 | 22.50 | 18.81 | 18.72 | 20.63 |
| Non-hydrogen atoms | 15456 | 15812 | 15868 | 15307 | 14792 | 14235 |
| macromolecules | 13590 | 13766 | 13556 | 13516 | 13224 | 13261 |
| ligands | 0 | 0 | 0 | 0 | 424 | 352 |
| solvent | 1866 | 2046 | 2312 | 1791 | 1144 | 622 |
| Protein residues | 1639 | 1638 | 1641 | 1644 | 1641 | 1639 |
| Rms (bonds) | 0.006 | 0.006 | 0.006 | 0.012 | 0.006 | 0.007 |
| Rms (angles) | 0.78 | 0.77 | 0.78 | 0.97 | 0.84 | 0.87 |
| Ramachandran |  |  |  |  |  |  |
| favored (%) | 99.51 | 99.51 | 99.08 | 99.26 | 99.45 | 99.45 |
| allowed (%) | 0.49 | 0.49 | 0.92 | 0.74 | 0.55 | 0.55 |
| outliers (%) | 0 | 0 | 0 | 0 | 0 | 0 |
| Rotamer outliers (%) | 0.90 | 0.69 | 1.66 | 0.95 | 1.17 | 2.00 |
| Clashscore | 1.48 | 1.98 | 2.08 | 2.04 | 1.44 | 2.81 |
| Average B-factor | 37.17 | 30.72 | 27.52 | 29.31 | 41.77 | 48.70 |
| macromolecules | 36.20 | 29.26 | 25.72 | 27.90 | 40.90 | 47.68 |
| ligands |  |  |  |  | 59.22 | 81.66 |
| solvent | 45.18 | 40.16 | 38.14 | 39.25 | 45.46 | 48.76 |
| PDB ID | 7MPL | 7MPM | 7MPN | 7MPO | 7MPP | 7MPQ |
| ^a^Statistics for the highest-resolution shell are shown in parentheses. | | | | | | |

| **Table S1b. Data collection and refinement statistics.^a^** | | | | |
| --- | --- | --- | --- | --- |
| ***Name*** | NrnC | NrnC | NrnC | NrnC•pGpG |
| ***Organism*** | *B. melitensis* | *B. melitensis* | *B. melitensis* | *B. melitensis* |
| ***Ligand*** | - | - | - | pGpG (partial) |
| ***Data collection*** | | | | |
| X-ray source | APS 24-ID-C | APS 24-ID-C | APS 24-ID-C | APS 24-ID-E |
| Wavelength | 0.9795 | 0.9795 | 0.9795 | 0.9792 |
| Resolution range (Å) | 74.56-1.42 | 111-1.45 | 93-1.75 | 54-1.72 |
| Space group | P422 | P4 | P422 | I41 2 2 |
| Unit cell a/b/c (Å) | 93.3 93.3 124.0 | 110.7 110.7 110.2 | 93.3 93.3 124.0 | 170.9 170.9 85.3 |
| Unit cell α/β/γ (°) | 90 90 90 | 90 90 90 | 90 90 90 | 90 90 90 |
| Total reflections | 1580788 (109208) | 2355312 (194722) | 888616 (81508) | 1095551 (107578) |
| Unique reflections | 103308 (10058) | 233668 (23187) | 55784 (5443) | 66925 (6617) |
| Multiplicity | 15.3 (10.9) | 10.1 (8.4) | 15.9 (15.0) | 16.4 (16.3) |
| Completeness (%) | 99.84 (98.78) | 99.91 (99.40) | 99.75 (99.07) | 99.92 (99.43) |
| I/sigma(I) | 30.58 (1.91) | 21.06 (1.86) | 26.42 (2.02) | 22.49 (3.04) |
| Wilson B-factor | 18.16 | 17.29 | 24.89 | 21.79 |
| CC1/2 | 1 (0.782) | 1 (0.702) | 0.999 (0.758) | 0.999 (0.878) |
| ***Refinement*** | | | | |
| Reflections (R-free) | 2000 (195) | 2016 (194) | 1998 (195) | 2000 (198) |
| R-work | 16.53 | 15.27 | 16.59 | 15.51 |
| R-free | 18.10 | 16.64 | 18.00 | 18.38 |
| Non-hydrogen atoms | 4019 | 8041 | 3823 | 3991 |
| macromolecules | 3303 | 6553 | 3253 | 3352 |
| ligands | 15 | 125 | 9 | 30 |
| solvent | 701 | 1363 | 561 | 609 |
| Protein residues | 410 | 815 | 407 | 410 |
| Rms (bonds) | 0.004 | 0.006 | 0.006 | 0.006 |
| Rms (angles) | 0.72 | 0.86 | 0.77 | 0.81 |
| Ramachandran |  |  |  |  |
| favored (%) | 99.26 | 98.88 | 99.25 | 99.00 |
| allowed (%) | 0.74 | 1.12 | 0.75 | 1.00 |
| outliers (%) | 0 | 0 | 0 | 0 |
| Rotamer outliers (%) | 0.55 | 0.42 | 0.28 | 0 |
| Clashscore | 1.21 | 1.72 | 0.77 | 1.34 |
| Average B-factor | 25.26 | 23.93 | 31.19 | 26.69 |
| macromolecules | 22.57 | 20.96 | 29.22 | 24.39 |
| ligands | 69.90 | 39.38 | 47.55 | 51.50 |
| solvent | 36.95 | 36.79 | 42.37 | 37.88 |
| PDB ID | 7MPR | 7MPS | 7MPT | 7MPU |
| ^a^Statistics for the highest-resolution shell are shown in parentheses. | | | | |
