# Supplementary material for: Structural characterization of NrnC identifies unifying features of dinucleases
Source: eLife. 2021 Sep 17;10:e70146. doi: 10.7554/eLife.70146 (PMC8492067; doi:10.7554/eLife.70146)
Supplement: Supplementary file 2. [file elife-70146-supp2.docx]

| **Supplementary File 2. Cryo-EM model validation statistics** | | | | |
| --- | --- | --- | --- | --- |
| Model | **NrnC*_Bh_*** | **NrnC*_Bh_*** | **NrnC*_Bh_*** | **NrnC*_Bh_*** |
| Ligand | **pGG** | **pAGG** | **pAAAGG** | **pAAAGG/Ca^2+^** |
| Symmetry | D4 | D4 | D4 | D4 |
| Composition (#) |  |  |  |  |
| Protein Chains | 8 | 8 | 8 | 8 |
| Nucleotide Chains | 8 | 8 | 8 | 8 |
| Atoms | 12760 | 11968 | 12296 | 12744 |
| Protein Residues | 1640 | 1592 | 1608 | 1640 |
| Nucleotide | 8 pGG | 8 pGG | 8 pGG | 8 pGG |
| Bonds (RMSD) |  |  |  |  |
| Length (Å) (# > 4𝜎) | 0.007 (0) | 0.003 (0) | 0.006 (0) | 0.007 (0) |
| Angles (°) (# > 4𝜎) | 0.721 (0) | 0.619 (0) | 0.717 (0) | 0.810 (0) |
| MolProbity score | 1.03 | 0.97 | 0.8 | 1.01 |
| Clash score | 2.48 | 1.98 | 1.01 | 2.29 |
| Ramachandran plot (%) |  |  |  |  |
| Outliers | 0.00 | 0.00 | 0.00 | 0.00 |
| Allowed | 0.49 | 1.03 | 0.51 | 0.99 |
| Favored | 99.51 | 98.97 | 99.49 | 99.01 |
| Rama-Z: Ramachandran plot Z-score (RMSD) | | | | |
| whole (N = 1754) | 1.28 (0.20) | 1.04 (0.21) | 1.24 (0.21) | 0.95 (0.20) |
| helix (N = 842) | 0.73 (0.19) | 0.79 (0.19) | 0.74 (0.19) | 0.78 (0.19) |
| sheet (N = 231) | 0.72 (0.23) | 0.50 (0.22) | 0.56 (0.23) | 0.48 (0.21) |
| loop (N = 681) | 1.33 (0.26) | 0.94 (0.28) | 1.35 (0.28) | 0.78 (0.26) |
| Rotamer outliers (%) | 0.66 | 0 | 0 | 0 |
| Cβ outliers (%) | 0 | 0 | 0 | 0 |
| CaBLAM outliers (%) | 0.5 | 0.52 | 0.52 | 0.5 |
| ADP (B-factors) |  |  |  |  |
| Protein (min/max/mean) | 31.65/64.33/43.25 | 23.08/54.02/35.37 | 22.76/59.16/38.31 | 21.29/58.88/31.57 |
| Nucleotide (min/max/mean) | 35.21/37.37/36.55 | 33.19/35.42/34.40 | 40.11/41.46/40.95 | 34.75/36.69/35.59 |
| Resolution Estimates (Å) |  |  |  |  |
| d FSC (half maps; 0.143) | 3.2 | 3.2 | 2.9 | 3.0 |
| d 99 (full/half1/half2) | 3.3/4.4/4.4 | 3.2/4.0/4.0 | 2.7/2.4/2.5 | 3.0/3.9/3.9 |
| d model | 3.3 | 3.3 | 2.8 | 3.2 |
| d FSC model (0/0.143/0.5) | 3.0/3.1/3.2 | 2.8/3.0/3.2 | 2.5/2.6/2.9 | 2.8/2.8/3.1 |
| Model vs. Data |  |  |  |  |
| CC mask | 0.86 | 0.83 | 0.87 | 0.86 |
| CC box | 0.81 | 0.75 | 0.72 | 0.79 |
| CC peaks | 0.77 | 0.72 | 0.69 | 0.75 |
| CC volume | 0.84 | 0.8 | 0.83 | 0.83 |
| PDB ID | 7MQB, EMD-23941 | 7MQD, EMD-23943 | 7MQF, EMD-23945 | 7MQH, EMD-23947 |

| **Table S2b. Cryo-EM model validation statistics** | | | | |
| --- | --- | --- | --- | --- |
| Model | **NrnC*_Bh_*** | **NrnC*_Bh_*** | **NrnC*_Bh_*** | **NrnC*_Bh_*** |
| Ligand | **pGG** | **pAGG** | **pAAAGG** | **pAAAGG/Ca^2+^** |
| Symmetry | C1 | C1 | C1 | C1 |
| Composition (#) |  |  |  |  |
| Chains | 8 | 8 | 8 | 8 |
| Nucleotide Chains | 8 | 8 | 8 | 8 |
| Atoms | 12556 | 12171 | 12678 | 12704 |
| Protein Residues | 1640 | 1629 | 1640 | 1640 |
| Nucleotide Ligands | 8 pGG | 8 pGG | 8 pGG | 8 pGG |
| Bonds (RMSD) |  |  |  |  |
| Length (Å) (# > 4𝜎) | 0.007 (0) | 0.008 (0) | 0.006 (0) | 0.006 (0) |
| Angles (°) (# > 4𝜎) | 0.806 (0) | 0.842 (0) | 0.781 (0) | 0.793 (0) |
| MolProbity score | 0.86 | 0.9 | 1.08 | 1.06 |
| Clash score | 1.31 | 1.58 | 2.87 | 2.71 |
| Ramachandran plot (%) |  |  |  |  |
| Outliers | 0.00 | 0.00 | 0.00 | 0.00 |
| Allowed | 0.49 | 1.74 | 1.17 | 1.35 |
| Favored | 99.51 | 98.26 | 98.83 | 98.65 |
| Rama-Z: Ramachandran plot Z-score (RMSD) | | | | |
| whole (N = 1754) | 1.03 (0.20) | 0.67 (0.20) | 0.73 (0.20) | 0.80 (0.20) |
| helix (N = 842) | 0.57 (0.18) | 0.34 (0.18) | 0.27 (0.18) | 0.50 (0.19) |
| sheet (N = 231) | 0.44 (0.24) | 0.22 (0.24) | 0.55 (0.22) | 0.59 (0.22) |
| loop (N = 681) | 1.22 (0.26) | 0.98 (0.27) | 1.07 (0.28) | 0.81 (0.26) |
| Rotamer outliers (%) | 0.61 | 0.77 | 0 | 0.59 |
| Cβ outliers (%) | 0 | 0 | 0 | 0 |
| CaBLAM outliers (%) | 0.5 | 0.76 | 0.75 | 0.81 |
| ADP (B-factors) |  |  |  |  |
| Protein (min/max/mean) | 30.00/71.38/49.55 | 30.00/80.81/50.16 | 19.92/70.75/36.57 | 31.09/95.82/52.94 |
| Nucleotide (min/max/mean) | 40.96/46.54/43.68 | 42.36/57.99/49.65 | 31.36/44.78/38.54 | 56.80/69.16/62.23 |
| Resolution Estimates (Å) |  |  |  |  |
| d FSC (half maps; 0.143) | 3.6 | 3.7 | 3.2 | 3.2 |
| d 99 (full/half1/half2) | 3.1/3.0/3.0 | 3.2/2.5/2.5 | 3.0/2.6/2.6 | 3.0/2.5/2.5 |
| d model | 3.4 | 3.5 | 3.1 | 3.2 |
| d FSC model (0/0.143/0.5) | 2.9/3.1/3.6 | 2.9/3.1/3.6 | 2.8/2.9/3.2 | 2.7/2.8/3.2 |
| Model vs. Data |  |  |  |  |
| CC mask | 0.86 | 0.81 | 0.82 | 0.81 |
| CC box | 0.81 | 0.75 | 0.72 | 0.67 |
| CC peaks | 0.77 | 0.69 | 0.67 | 0.62 |
| CC volume | 0.84 | 0.79 | 0.79 | 0.77 |
| PDB ID | 7MQC, EMD-23942 | 7MQE, EMD-23944 | 7MQG, EMD-23946 | 7MQI, EMD-23948 |
